# Environment geometry alters sequential route learning and its integration into cognitive maps

**DOI:** 10.1038/s41467-026-75129-y

**Published:** 2026-07-07

**Authors:** Jaida Long, Estibaliz Herrera, Yiran Li, Felipe Oliveira, Rida Ahmed, Sana Hussain, Camille Rivera, Kristin Liquori, Thackery Brown

**Affiliations:** 1 Georgia Institute of Technology, Atlanta, GA USA; 2 Center for Research and Education in Navigation, Atlanta, GA USA; 3 University of Waterloo, Waterloo, ON Canada; 4 Georgia State University, Atlanta, GA USA; 5 Albizu University, San Juan, Puerto Rico USA

**Keywords:** Human behaviour, Spatial memory

## Abstract

Researchers hypothesize that geometric irregularities can impede spatial memory by distorting neural metrics of space provided by entorhinal grid cells. However, irregularly-shaped geometries could also contribute orientation information that benefits spatial memory. Moreover, serial order effects could counteract or amplify geometry effects during route navigation. Our study directly tests how effects of environment geometry (trapezoid and square) and series in route navigation interact to influence spatial memory in a virtual navigation task. We uncover effects of environment geometry predicted by grid cell models and serial order effects predicted by path integration models. Critically, our results demonstrate that these effects combine in an additive manner, such that location memory toward the end of a route in regions with the largest geometric irregularities (trapezoid) yield the worst spatial memory. Additionally, self-report measures of navigational ability are associated with increased sensitivity to geometric irregularities, emphasizing the essential role of individual differences in navigation.

## Abstract (web summary)

Theories disagree whether environments with irregular layouts impair spatial memory or help us stay oriented. The authors show that spatial memory can be enhanced or disrupted depending on where we are along a route and relative to misaligned walls.

## Introduction

Spatial navigation is often complex in the real world. The spaces in which we navigate contain boundaries and affordances with a variety of arrangements that influence and inform our behavior^1–9^. In rodents, misalignment between boundaries disrupts neural activity of a theorized biological basis of internal maps, grid cells^1,10,11^. Identical boundary distortions also impede human spatial memory for object locations in a manner commensurate with and predicted by rodent grid distortions^12^. Furthermore, individual navigators are diverse in their ability for spatial processing, such as responding to environmental boundaries and affordances, and encoding a series of spatial information^13–17^. Yet, it has not been characterized how these factors of boundaries, series, and individual differences interact to influence spatial cognition and subsequent behavior.

The influence of boundaries on spatial memory is, critically, variable. In some paradigms, distortions in boundaries disrupt spatial memory^10,12^. For example, Bellmund et al. 2020 report that spatial memory for single object locations is worse in trapezoids compared to squares in humans^12^. Grid cell recordings from rodents provide a potential mechanism, demonstrating distorted firing fields in trapezoids, particularly the narrow end^10^. Furthermore, a recent study not only replicated the distortion of rodent grid cell firing fields in trapezoid environments, but also observed significant correlations between these distortions and spatial memory deficits in those same rodents^18^. On the other hand, other literature suggests learning in trapezoid environments could, theoretically, benefit from geometric distinctiveness^19,20^. In certain designs, the symmetry of squares contributes to more orientation uncertainty compared to trapezoids, which predicts worse spatial memory in square environments^21^. Paradigms with low uncertainty benefit from improved updating of cognitive maps and navigational performance^19,20^. Thus, one could have different predictions for how the geometry of environments would influence the accuracy of spatial memory. One perspective is that the distortion of grid cell firing fields in asymmetrical environments (trapezoids) would overpower any benefit from geometric distinctiveness. In the competing perspective, geometric distinctiveness could counteract or even reverse the effects of distorted grid cell firing fields; spatial memory accuracy could be lower for a square environment due to higher ambiguity in geometric cues compared to trapezoids. The present study tested these alternatives.

In addition to traversing boundaries, navigation involves successive locations that must be learned together. Collectively, a series of locations together constitute a “route”. Previous research has typically looked at individual object location memory or path integration, but not the intersection between the two, which is commonplace in real-world navigation^12,22^. Spatial memory in route navigation is critically different from these paradigms due to serial order effects, which can manifest in several ways. One hypothesis emphasizes primacy and recency effects: in a variety of sequential learning tasks, including navigation, primacy and recency often result in bowed serial position functions where memory is worse at the middle of a series^23–29^. Alternatively, statistical learning/schema processes could continuously improve learning and retrieval along the route as more cues become available^30,31^. Finally, a progressive accumulation of path integration error during route navigation may overwhelm any benefit of recency effects or statistical learning^22,32^. Furthermore, each of these manifestations of serial order effects could combine with the geometry effects of square and trapezoid environments. For example, primacy and recency effects in grid cell models would predict the worst spatial memory at the middle of a route, and this would be exacerbated in trapezoids (where other disruptive mechanisms are at play), while path integration and grid cell models would predict the worst spatial memory at the end of a route in trapezoids. To this end, we tested the competing perspectives of serial order effects in navigation and how they interact with environment geometry.

Navigation research has shown that the influence of boundaries and routes on spatial cognition varies between individuals^14,15^. An individual’s aptitude for performing wayfinding and knowing one’s location, an ability termed sense of direction (SOD)^33^, is variable. Only some navigators are able to use landmarks and cues flexibly and integrate them into internal maps of their environment^14^. For these “good” navigators, this ability is linked to significantly improved performance in navigation. The Santa Barbara Sense of Direction (SBSOD) scale^34^ is a metacognitive measure of SOD that predicts performance in naturalistic wayfinding, virtual, and vicarious navigation^16,17,35^. Critically, we have employed the SBSOD to consistently show that SOD significantly moderates relationships between a) spatial cue visibility and route learning processes, and b) memory load, interference, and configural learning outcomes of navigational experiences^13,16,17^. Following this, SOD is strongly hypothesized to moderate the interaction of boundaries and routes in spatial memory. High SOD individuals may be more sensitive to geometric cues from irregular boundaries, owing to their increased ability to integrate these cues^13,17^. Accordingly, high SOD should benefit learning in the presence of distorted boundaries. However, a competing perspective is that increased sensitivity to visual cues may be detrimental: boundary distortions could cause disproportionately larger distortions in high SOD individuals’ cognitive maps. Therefore, the interaction between individual differences and geometry in spatial memory remains unclear and is an important avenue for elaboration on recent literature^12^.

**Fig. 1:**
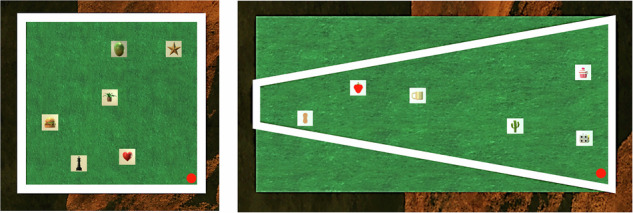
Overhead view of the task. Examples of a square and trapezoid environment are displayed, along with icons indicating the starting point (red dot), and the identities and locations of the objects that were present in each respective environment (details in “Supplementary Discussion”). During the task, participants navigated first-person between the six object locations within environments (two squares and two trapezoids) along a fixed route to learn the objects’ locations and sequence. Then, participants’ spatial memory for object locations was assessed from first-person and overhead perspectives.

Here, we directly test these competing perspectives by investigating how environmental boundaries, serial order effects, and individual differences interact with one another to influence and predict spatial memory in a navigation paradigm. Participants complete a virtual navigation task (Fig. 1) where they learn routes of six object locations in square environments and in environments where the parallel structure is warped in the trapezoids. Importantly, square environments do not distort grid cell firing fields as much as trapezoids, but they contain more uncertainty in geometric cues for orientation estimation. Memory of the object locations is then assessed through both first-person and overhead tests. We show significant serial order effects on individual object location memories: in both first-person and overhead tests, spatial memory worsens along the route for both shapes. Additionally, consistent with grid cell-based predictions^10,12^, spatial memory for objects in the narrow end of the trapezoid is worse than in the wide end and in the square. We show that these factors combine in an additive manner: objects toward the end of the route in the narrow end of the trapezoid yield the worst spatial memory. Finally, we demonstrate that high SOD individuals’ spatial memory has increased sensitivity to the distorted boundary cues of the trapezoid, particularly in the overhead test.

## Results

Participants completed a virtual desktop navigation task where they learned the locations and sequence of six objects in four open-field environments (Fig. 1). Each environment’s set of six objects was distinct from the other three environments (details in “Supplementary Discussion”). Object positions were organized into two triplets per environment, one in each half of the environment. Participants were familiarized with navigation in a fifth practice open-field environment before the main task. In the main task, each participant navigated two trapezoid environments and two square environments. The square and trapezoid environments were of equal surface area, and the wall colors and textures were a uniformly smooth gray. Object positions in each environment had matched relative angles and inter-object distances (see “Methods”). In the background, mountains, clouds, and the sun served as distal orientation cues; there were no orientation cues within the environment other than the walls, resulting in a square environment with ambiguous orientation information. Within each environment, participants completed three trials of route learning and route testing. Route learning and testing were assessed with a serial object-placement task in first-person. Afterward, object-location testing was performed once more from an overhead perspective. Participants were not able to view the complete configuration of the boundaries in the overhead test, emphasizing the learned relational knowledge of relative object locations and their configuration along the route. Performance in all tests was assessed by calculating the Euclidean distance between participants’ remembered locations of the objects and their true locations, termed spatial memory error (SME). Spatial memory error was measured on a different scale for the first-person and overhead tests, denoted SME_FP_ and SME_O,_ respectively, in the figures below.

### Simple effects of environment geometry

From the seminal grid cell work in rodents and Bellmund et al.’s 2020 behavioral study^10,12^, we would expect to observe higher spatial memory error in trapezoid environments than square ones. However, other work from cognitive psychology indicates that orientation ambiguity due to, e.g., the symmetrical nature of square environments, can impair learning^19–21^. Therefore, the greater geometric distinctiveness of different locations in a trapezoidal space might facilitate learning. We employed mixed-effects modeling to test these competing frameworks, testing for an environment geometry effect in our different design/task context. In this model, mean spatial memory error across a trial for the first-person test (averaging spatial memory error for all six objects) was predicted by the fixed factors: environment shape (square or trapezoid), trial (1–3), and environment presentation order (1–4); subject ID was a random factor. Collapsing across locations in the environment, first-person spatial memory error was not significantly different in the trapezoid vs. the square environments (*F*(1,796.12) = 0.453, *p* = 0.501) (Supplementary Fig. 1), inconsistent with both the grid cell-based distortion and geometric distinctiveness framework. The same model produced a similar result in the overhead test (*F*(1, 210.41) = 0.200, *p* = 0.655) (Supplementary Fig. 1). Taken together, our results collapsing across locations in a route suggest that environment geometry effects were not present—on average—when extended to a serial object-location (route) learning task.

### Simple effects of serial order

**Fig. 2:**
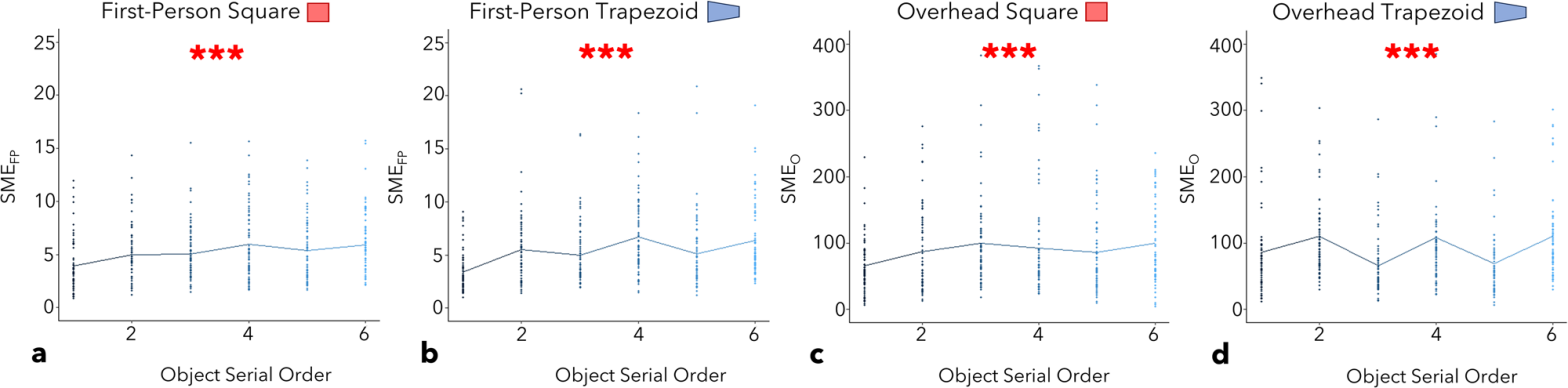
Spatial memory error by object serial order. Spatial memory error (SME) is plotted by the object order within the route for each participant (*n* = 75) in first-person (FP) and overhead (O) tests. Mixed effects models revealed a significant effect of object order (indicated by asterisks; *p* < 0.001 for all, uncorrected for multiple comparisons) for: **a**, **b** first-person test square and trapezoid environments, and **c**, **d** overhead test square and trapezoid environments. Analyses are computed within-subjects; there is no control group. For visualization, each participant’s data was averaged to one data point per object, and some outliers were excluded.

Leveraging our design, we unpacked the serial order effects in individual object-location memories and tested the complementary theoretical perspectives highlighted in the introduction: (1) Spatial memory error could demonstrate a quadratic effect, mirroring bowed serial position functions with error being greatest towards the middle^23–29^, (2) Spatial memory error could increase progressively as participants complete the route due to path integration error accumulation^22,32^, or (3) Spatial memory error could decrease as participants progress along the route. Schema and statistical-learning mechanisms could allow location memory of later objects to benefit from recalling locations for previous objects as the route progresses^30,31^. To test these alternative hypotheses, we constructed a mixed-effects model predicting individual object spatial memory error using the object serial order within a route (1–6) as an additional fixed factor. This model was otherwise identical to the “Environment Geometry” model used above (details in Supplementary Tables 3 and 4). Importantly, there was a significant effect of serial order on spatial memory error in both the first-person test (*F*(5, 5140.17) = 33.630, *p* < 0.001) and the overhead test (*F*(5, 1583.59) = 10.964, *p* < 0.001), demonstrating that object order significantly modulated spatial memory error (Supplementary Fig. 2). Since spatial memory error was progressively worse towards the end of the route (Fig. 2), simple effects of serial order are best explained by theories of path integration error accumulation. Furthermore, while serial order effects were marked in both tests, we note that the effect of serial order on spatial memory error was numerically stronger in the first-person test than in the overhead test, consistent with active navigation and path-integration demands influencing self/object localization outcomes.

### Environment geometry by serial order

Critically for our study, these serial order effects were not independent of the geometry manipulation. The mixed-effects models demonstrated that there was a significant interaction between environment geometry and serial order in both the first-person test (*F*(5, 5140.17) = 2.420, *p* = 0.034) and the overhead test (*F*(5, 1583.60) = 8.600, *p* < 0.001) (details in Supplementary Tables 3 and 4). To unpack this interaction, we next investigated the impact of serial order on spatial memory error in separate mixed-effects models for each environment shape. We observed strong effects of the object order factor on spatial memory error within both shapes in the first-person test (Fig. 2a Square: *F*(5, 2536.03) = 12.175, *p* < 0.001; Fig. 2b Trapezoid: *F*(5, 2530.33) = 23.888, *p* < 0.001), with the relative *F*-values revealing that the object order by environment geometry interaction in the preceding omnibus model was driven by a larger effect in the trapezoids. These patterns of object order on spatial memory error held consistent across the shapes in the overhead test as well (Fig. 2c Square: *F*(5, 729.60) = 6.412, *p* < 0.001; Fig. 2d Trapezoid: *F*(5, 778.14) = 15.443, *p* < 0.001). Qualitatively, the pattern was more clearly linear across the serial order in the square than the trapezoid—a pattern explained by our hypothesis testing below.

### Trapezoid subregion

**Fig. 3:**
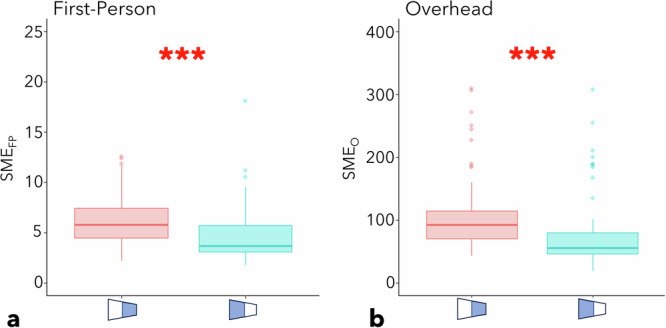
Spatial memory error by trapezoid subregion. Spatial memory error (SME) is plotted by the narrow and wide halves of the trapezoid for each participant (*n* = 75) in **a** the first-person (FP) test (narrow: minima = 2.20, maxima = 12.57, center = 5.81, box bounds = [Q1 4.48, Q3 7.43], whisker bounds = [2.20, 11.71], IQR = 2.95) (wide: minima = 1.83, maxima = 18.09, center = 3.72, box bounds =  [Q1 3.13, Q3 5.72], whisker bounds = [1.83, 9.59], IQR = 2.59), and **b** the overhead (O) test (narrow: minima = 44.05, maxima = 309.72, center = 93.08, box bounds = [Q1 70.54, Q3 115.25], whisker bounds = [44.05, 159.91], IQR = 44.71) (wide: minima = 19.64, maxima = 307.32, center = 55.91, box bounds = [Q1 46.25, Q3 79.90], whisker bounds = [19.64, 100.91], IQR = 33.65). Mixed-effects models revealed a significant effect of trapezoid subregion as indicated by the asterisks (*p* < 0.001 for both uncorrected for multiple comparisons). Analyses are computed within-subjects; there is no control group. For visualization, each participant’s data was averaged to one point per trapezoid subregion, and some outliers were excluded.

Altogether, these results indicate that geometry and serial position combine to influence object-location error in learning along complex trajectories. Geometric theories from grid cell literature would predict this interaction to be driven by the trapezoid, which yields higher spatial memory error for objects located in its narrow half, compared to the wider half^12^. This prediction is grounded in the observation that grid cell firing fields distort as animals enter the more narrow end of such trapezoid environments^10^. To test this, we constructed a mixed-effects model for the trapezoid data in which each object was coded as either being in the narrow or wide half (additional fixed factors: environment presentation order and trial; random factor: subject ID). As predicted, narrow vs. wide significantly modulated spatial memory error in both the first-person test (Fig. 3a: *F*(1, 2578.33) = 76.056, *p* < 0.001) and overhead test (Fig. 3b: *F*(1, 794.15) = 87.255, *p* < 0.001).

### Trapezoid subregion versus square

We observed that the narrow-wide aspect of the trapezoid underlied the shape-by-serial-order interaction in our above results. In the trapezoid, the routes broadly alternated between narrow and wide ends; objects of even serial orders (2,4,6) were in the narrow end, while objects of odd serial orders (1,3,5) were in the wide end; this explained the visual sawtooth pattern in Fig. 2’s trapezoid data. Although environment geometry effects were not present when collapsing across trapezoid subregions, we hypothesized that comparing each trapezoid subregion (narrow or wide) separately to the square would uncover greater environment geometry effects. Specifically, grid cell-based theoretical frameworks would predict that (1) objects in the narrow end of the trapezoid would have higher spatial memory errors relative to those in the square, and (2) the effect would be less pronounced when comparing objects in the wide end of the trapezoid to those in the square. We constructed two mixed-effects models to investigate this prediction (fixed factors: Shape, environment presentation order, trial; random factor: subject ID). One model compared the narrow end to the square; the other model compared the wide end to the square. Additionally, each model controlled for object serial order.

**Fig. 4:**
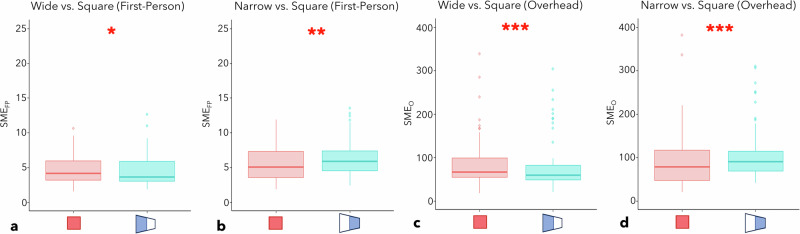
Trapezoid subregion vs. square. Participant (*n* = 75) spatial memory error (SME) is plotted by Shape (trapezoid subregion vs. square). Mixed-effects models revealed a significant effect of Shape for **a** wide vs. square in the first-person (FP) test (*p* = 0.049 uncorrected for multiple comparisons) (wide: minima = 1.83, maxima =  12.64, center = 3.63, box bounds = [Q1 3.06, Q3 5.92], whisker bounds = [1.83, 9.22], IQR = 2.86) (square: minima =  1.56, maxima = 10.60, center = 4.16, box bounds = [Q1 3.22, Q3 5.95], whisker bounds = [1.56, 9.55], IQR = 2.74), **b** narrow vs. square in the first-person test (*p* =  0.002 uncorrected for multiple comparisons) (narrow: minima =  2.36, maxima = 13.52, center = 5.90, box bounds = [Q1 4.57, Q3 7.43], whisker bounds = [2.36, 11.48], IQR = 2.86) (square: minima = 1.89, maxima = 11.92, center = 5.11, box bounds = [Q1 3.56, Q3 7.31], whisker bounds = [1.89, 11.92], IQR = 3.75), **c** wide vs. square in the overhead (O) test (*p* = 0.001 uncorrected for multiple comparisons) (wide: minima = 19.64, maxima = 305.19, center = 58.12, box bounds = [Q1 43.96, Q3 80.50], whisker bounds = [19.64, 104.44], IQR = 36.54) (square: minima = 17.83, maxima = 338.98, center =  67.19, box bounds = [Q1 55.31, Q3 99.47], whisker bounds =  [17.83, 157.84], IQR = 44.16), and **d** narrow vs. square in the overhead test (*p* < 0.001 uncorrected for multiple comparisons) (narrow: minima = 43.39, maxima = 309.72, center = 93.08, box bounds = [Q1 71.84, Q3 117.33], whisker bounds = [43.39, 179.29], IQR = 45.49) (square: minima =  20.22, maxima = 382.31, center = 78.41, box bounds = [Q1 47.40, Q3 116.98], whisker bounds = [20.22, 219.75], IQR = 69.58). Analyses are computed within-subjects; there is no control group. For visualization, each participant’s data was averaged to one point per Shape category per plot, and some outliers were excluded.

As predicted, we observed a significant Shape effect when comparing the narrow end to the square in both the first-person and overhead test (Fig. 4b first-person: *F*(1, 2471.59) = 9.661, *p* = 0.002; Fig. 4d overhead: *F*(1, 715.26) = 12.424, *p* < 0.001), wherein spatial memory error was significantly greater in the narrow end. When comparing the wide end to the square, there was once again a significant difference in the first-person and overhead tests (Fig. 4a first-person: *F*(1, 2471.01) = 3.885, *p* = 0.049; Fig. 4c overhead: *F*(1, 757.26) = 10.201, *p* = 0.001), but in the opposite direction: spatial memory error was higher in the square than the wide end of the trapezoid. Unlike the narrow end, this was consistent with the alternative, facilitative trapezoid effect from a geometric distinctiveness framework, and could indicate that when there is only a moderate level of uncertainty from local influences on metric information (e.g., grid cell firing being relatively spared in the wide end), navigators are in a “sweet spot” in which orientation can benefit from additional landmark information from both the environment polarity and the distal cues^36^.

### Individual differences

Not all navigators are equally able to integrate landmarks and environmental cues into cognitive maps^14^, and we and others have consistently shown that sense of direction (SOD) significantly moderates relationships between (a) spatial cue visibility and route learning processes and (b) memory load, interference, and configural learning outcomes of navigational experiences^13–17^. Whether or not participants could accurately leverage cues from boundary configurations or become disoriented by them in their cognitive maps was central to our task. Therefore, we predicted different behavioral memory effects between individuals of high and low scores on the Santa Barbara Sense of Direction Scale (SBSOD)^34^. Typically, higher SOD is associated with increased performance in spatial tasks, owing to an increased ability to integrate cues. This would suggest that high SOD individuals would perform better in the trapezoid than low SOD individuals. However, the grid cell-based distortion framework leads to an intriguing alternative prediction: high SOD individuals’ increased sensitivity to boundaries and cues in the environment^13,17^ may yield disproportionately larger distortions in their cognitive maps if those cues distort the sense of space (as they do grid cell firing) and subsequent behavioral memory effects. This would result in high SOD individuals performing worse in the trapezoid compared to low SOD individuals. We employed mixed-effects modeling to test these competing perspectives.

First, at the broadest levels of our task, we found higher SOD predicts increased performance (lower spatial memory error) when collapsing across other factors in the first-person test (*F*(1, 73.35) = 6.237, *p* = 0.015) (not statistically significant in the overhead test; Supplementary Fig. 3; *F*(1, 72.35) = 3.092, *p* = 0.083) (mixed-effects model fixed factors: Shape, environment presentation order, trial, SOD; random factor: subject ID). Turning to the critical questions of our study, we hypothesized that the influence of Shape on spatial memory would be stronger in high SOD individuals than in low SOD individuals. This could manifest in two ways: higher SOD could (1) yield better performance in the trapezoid because of an increased ability to integrate environment cues, or (2) yield worse performance because of larger distortions in cognitive maps due to increased cue sensitivity. To test this, we focused on the Shape*SOD interaction term in the same mixed-effects models. The Shape*SOD interaction was not significant in the first-person test (*F*(1, 773.28) = 0.258, *p* = 0.611), but was clear in the overhead test (*F*(1, 204.48) = 6.766, *p* = 0.010) (details in Supplementary Tables 5 and 6).

**Fig. 5:**
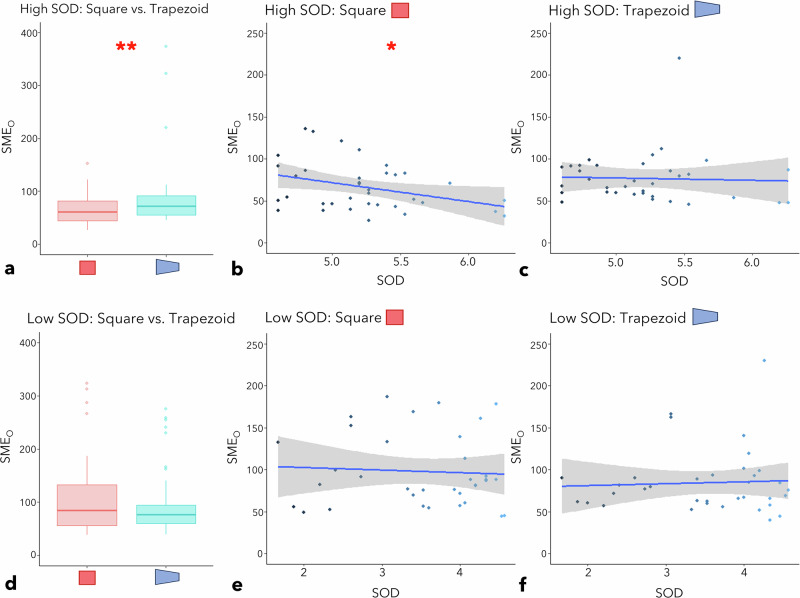
Overhead test spatial memory for high and low SOD. Spatial memory error (SME) in the overhead test (O) is plotted by Shape for **a** high sense of direction (SOD) individuals (*n* = 38) (square: minima = 26.60, maxima =  152.66, center = 61.10, box bounds = [Q1 44.49, Q3 81.36], whisker bounds = [26.60, 121.73], IQR = 36.87) (trapezoid: minima = 45.58, maxima = 374.23, center = 72.13, box bounds = [Q1 55.10, Q3 91.29], whisker bounds = [45.58, 112.13], IQR = 36.19), and **d** low SOD individuals (*n* = 37) (square: minima = 38.27, maxima = 323.78, center = 84.34, box bounds = [Q1 56.44, Q3 132.63], whisker bounds =  [38.27, 186.86], IQR = 76.19) (trapezoid: minima = 39.67, maxima 275.95, center = 76.61, box bounds = [Q1 60.34, Q3 94.70], whisker bounds = [39.67, 140.69], IQR = 34.36). SME is plotted by SOD for high SOD individuals in **b** the square, **c** and the trapezoid (blue line: model prediction; gray shading: error bounds of model prediction). Mixed-effects models revealed that for high SOD individuals, the effect of Shape was significant (**a**: *p* = 0.010 uncorrected for multiple comparisons), and the effect of SOD was significant in the square (**b**: *p* = 0.011 uncorrected for multiple comparisons), as indicated by the asterisks. SME is plotted by SOD for low SOD individuals in **e** the square, and **f** the trapezoid (blue line: model prediction; gray shading: error bounds of model prediction). Analyses are computed within-subjects; there is no control group. For visualization, each participant’s data was averaged to either one point total (**b**,** c**,** e**, and **f**) or one point per shape (**a**,** d**), and some outliers were excluded.

To formally test if this interaction was driven by increasing sensitivity to Shape in high SOD individuals, we median-split high (SOD ≥ 4.6) and low SOD (SOD < 4.6) individuals’ overhead test data and re-computed the parameters. Low SOD individuals demonstrated null results for all effects (Fig. 5d–f). By contrast, high SOD individuals showed a significant Shape effect (Fig. 5a; *F*(1, 99.01) = 6.890, *p* = 0.010), and retained the significant SOD*Shape interaction (Fig. 5b, c: *F*(1, 99.17) = 7.958, *p* = 0.006). To adjudicate whether this indicates higher SOD benefits or impairs performance in the presence of irregular geometric cues, we tested these relationships with spatial memory error separately in each environment shape (fixed factors: environment presentation order, trial, SOD; random factor: subject ID). Consistent with the broader literature, higher SOD showed significantly lower spatial memory errors in the square (Fig. 5b; *F*(1, 37.17) = 7.218, *p* = 0.011). However, the relationship was null in the trapezoid (Fig. 5c; *F*(1, 38.05) = 0.004, *p* = 0.952), showing that irregular boundaries reduce the benefit of increasing SOD on spatial memory error. It should be noted that we did not correct these analyses for multiple comparisons because they were targeted post hoc tests specifically to identify which relationships drove the significant result in the omnibus model.

### Procrustes analysis

Our primary analyses measured spatial memory performance by the Euclidean distance between true object locations and participants’ responses. To precisely account for initial or overall reference errors (e.g., scaling, translation, rotation, reflection), possibly clouding the underlying structure of spatial memory error, we performed a secondary set of analyses to determine the patterns of spatial memory error accounting for such overall referencing errors. To do this, the raw data for these analyses were Procrustes-transformed in MATLAB, thereby controlling for the effects of scaling, translation, rotation, and reflection on the overall response profile of our measured Euclidean errors. The analyses of Procrustes-transformed data revealed effects overall that were consistent with those from the raw data. Qualitatively, which effects were statistically significant or non-significant remained the same across our error measures of interest with the Procrustes transforms (details in “Supplementary Discussion”).

### Manipulations of cue availability

Several types of environmental cues were available to participants during the task, including distal cues (mountains, clouds, and sun) and proximal or local cues (environment walls with the same color and texture). Since environmental cues and their proximity can significantly influence spatial memory, we ran a third set of analyses to determine the generalizability of our results to environments with different cue availability (i.e., local instead of distal landmark information). We collected a new cohort of healthy young adults (*N* = 82, 52 females indicated by self-report gender, age = 20.049 ± 2.233) from Georgia Tech and the surrounding Atlanta area, called the Non-Global cohort, who performed the task identically to the previous cohort, called the Global cohort. Information regarding the selection of sample size, study protocol approval, obtaining informed consent, compensation, blinding, and gender-based analyses was identical to the Global cohort (details in “Methods”). We presented similar square and trapezoid environments again, with the same 6-object routes and the same pseudorandomized, counterbalanced presentation of environments. The only difference for this new cohort was the environmental cues themselves: distal cues were removed, and the environmental walls were changed to have distinct colors and textures (local cues) in both environments, thus replacing distal with proximal landmark information (details in “Supplementary Discussion”). The same analyses were replicated for this Non-Global condition (details in “Supplementary Discussion”). Four participants were excluded from the Non-Global condition analysis due to invalid data. Importantly, in qualitative terms, the majority of the results we report from the Global condition maintained the same pattern of statistical significance in this Non-Global condition.

## Discussion

Spatial memory in real-world navigation is influenced by numerous factors, including the presence of boundaries, route demand characteristics, and individual differences in navigation abilities^2–6,12–17^. It remains unclear how these factors interact. Grid cell-based theories predict that distorting boundaries worsens spatial memory^10,12^, while some cognitive psychology theories articulate how distorted boundaries could instead decrease orientation/position uncertainty and thus enhance spatial memory^19–21^. There are similar competing perspectives for how sequential learning processes for routes influence location memory: primacy-recency effects would predict worse memory at the middle of routes^23–29^, statistical learning/schema processes predict worse memory at the beginning^30,31^, and path integration error accumulation predicts worse memory at the end (absent any correction mechanism)^22,32^. Furthermore, the influence of boundaries, cue uncertainty, and route navigation demands all depend on individual spatial abilities and propensities for relying on and integrating the available cues in memory^13–17^.

Our study directly tested these competing perspectives and quantified how these three factors of environment geometry, serial order effects, and individual differences in SOD interact in navigation. Environment geometry and serial order effects combined in an additive manner consistent with grid-cell and path integration theories: spatial memory was the worst at the end of an environment’s route and was exacerbated with more distorted geometry (the trapezoid). Individuals with high subjective ratings of spatial abilities (SOD) performed better overall, consistent with past literature; however, in overhead tests of spatial memory, these individuals also demonstrated increased sensitivity in spatial memory relative to environment shape (square vs. trapezoid) at encoding, supporting our growing body of literature that higher SOD may not inherently benefit spatial task performance. Rather, it measures a tendency to rely on and integrate spatial cues^13,17^; here we show that when boundaries distort the sense of space, this can be to the memory’s detriment.

Our first analyses focused on simple effects of environment geometry. Many studies have linked the integrity of grid cells to environment boundaries, especially those with varying geometry^1,11,37–40^. In highly polarized enclosures, such as trapezoids, distortions to grid cell firing fields are more severe^10,12^. Computational models demonstrate significantly impaired positional decoding in such enclosures^12^, and trapezoids distort grid patterns in rodents and the accuracy of spatial memories in humans, respectively^10,12^. Both effects have been shown to be more pronounced in the narrow end, the most polarized region of the trapezoid.

However, geometric distinctiveness can also aid navigation by providing unambiguous orientation cues (i.e., the geometry alone helps distinguish locations in the North from the South of an enclosure)^19–21^. Trapezoidal boundaries can provide such enriched cueing^21^. Contrasting the grid cell-based “disruption” framework, some studies have shown that low geometric distinctiveness, including increasing the symmetry of environments, impairs spatial reorientation and navigation^19–21^. In fact, disrupting rodent head direction signals (as can occur with ambiguous heading cues) impairs grid cell signaling, providing a mechanism for how geometric distinctiveness could influence spatial memory^41^. Direction-related memory in trapezoid-shaped environments has been found in some studies to benefit from geometric distinctiveness, especially in the absence of other orientation cues^42–46^. It is noteworthy that in our study, both the square and the trapezoid were trained with distal background cues, essential for reorientation in the square environment, but which might be detrimental in a trapezoid, especially in the most informative portion of the environment (i.e., the narrow end) as they provide redundant information^3,4,7^. Given the prior literature^10,12^, our theoretical focus was on grid cell-based perspectives of our environment manipulation, but having multiple cues in this may have also increased the cognitive load, leading to competition, and thus, contribute to the worse performance^3–5,7–9,36,47–49^. For example, the reliable orientation information provided by distal cues (which is constant regardless of location within the local environment) may have been in competition/conflict with the distorted depth and orientation information provided by the proximal environment boundaries in the trapezoids (which depends on the navigator’s current perspective). Importantly, these disruptive and facilitative mechanisms can co-exist: benefits to orientation of a navigator can offset/be offset by disruption in distance/positional estimation.

Our study sheds light on these competing perspectives. In our design, we demonstrate a clear effect of significantly worse spatial memory in the narrow end of the trapezoid compared to the square. This is predicted by existing grid cell studies that suggest that trapezoidal boundaries distort grid cell firing fields and, subsequently, spatial memory. However, these studies also predict that spatial memory in the wide end of the trapezoid is worse than in the square. Not only was this effect absent in our results, but we also demonstrate evidence for the opposite: that spatial memory was worse in the square than the wide end of the trapezoid. We posit that other factors, such as geometric distinctiveness, may interact with grid cell-based mechanisms for the ultimate outcomes of geometry on spatial memory: namely, geometric distinctiveness may benefit spatial memory (as others have shown), but the benefits can be offset by metric distortions in memory from grid cell disruptions. With this interaction in place, we theorize that when grid cell firing patterns are minimally distorted, as we know is the case in the wide end of a trapezoid, impairments may be minimal or facilitation effects may even be observed. It is plausible that if we assessed self-orientation relative to the objects (as other literatures have done^21^) rather than the object locations themselves, we would observe better performance in the trapezoid—but our data importantly emphasize that distorting environment boundaries (trapezoid) correspondingly distorts object location memories, rather than improving them. Supporting this, our supplemental Procrustes analysis of this result revealed that the benefit in spatial memory in the wide end of the trapezoid was accounted for by rotational and translational referencing but not scaling (other factors the analysis tested for).

An important advantage in our study that extends the past studies on geometry^12^ is our examination of object-location memory as a function of encoding sequential “routes”, as is the case for many real-world situations where configural knowledge, the knowledge of spatial relationships between objects and their features, is encoded. Serial order effects are ubiquitous in serial memory across a wide range of behavioral tasks and can influence performance in several ways^22–32^. We viewed our study as an opportunity to test which of these frameworks better fit our observed data and how serial order effects interact with the influences of geometry.

First, for serial order memory in non-navigation settings, primacy and recency effects often combine to produce a bowed serial function, where memory is worse towards the middle of a series^23–29^. Given that mapping the trajectory of object-location pairings in our task is a serial order problem that can rely to varying degrees on working memory^17^, a bowed serial memory function with worse spatial memory accuracy at the middle of the route was a plausible outcome—perhaps exacerbated in the trapezoid if metric distortions interact with these factors. Evidence for such curves in the navigation domain is less common, but some studies have shown recency and primacy effects in recalling directions^25^ or landmarks^26,29,50^. Second, path integration processes might give rise to an alternative pattern by which serial order effects can modulate spatial memory^22,32^. Researchers have proposed that path integration is foundational for building first-person spatial knowledge^22^. This process is vulnerable to random measure errors, and errors can accumulate in location+orientation memory as routes progress^51^. Neurally, grid cells are hypothesized to provide a mechanism of path integration^52,53^, and models have shown that noise in grid cells can contribute to error accumulation^54–56^. While the link between grid cells and path integration processes is not definitive, we noted there is a theorized mechanistic overlap between the path integrator-based prediction for potential serial order effects and geometry distortion effects (discussed above). Finally, we also tested for a pattern consistent with a third class of theoretical frameworks, which could yield another distinct pattern of serial order effects: schema and statistical-learning mechanisms may continuously improve performance as the route progresses^30,31^. This is because objects within the environment can serve as mnemonic landmarks from which participants can improve orientation and distance information when learning about new subsequent spatial associations^57,58^. Under these theoretical umbrellas, subsequent object placements may benefit from the increasingly detailed configural space.

Our analyses showed that spatial memory error was significantly worse at the end of the route, an effect that was particularly clear in square environments where boundary distortion was not an added factor. This pattern is better explained by theories of path integration error accumulation, which partially attribute a continuous worsening of spatial memory error over the course of the trajectory to noise in grid cell activity^22,32^. While our present study does not measure grid cell firing in humans, it is notable that—like geometry distortion effects discussed above —the serial order effects that we observed were more consistent with predictions grounded in path integration research, where grid cells are also believed to play a critical role.

The interface between serial order effects and the geometry manipulation in our design was particularly interesting from a theoretical perspective. Overall, we established that these effects combined additively, such that spatial memory error was worse at the end of a route for objects at the narrow end of the trapezoid. However, some views suggest that the presence of boundaries in our task should reduce the effects of path integration error accumulation. Setting geometric distortions aside, locations close to boundaries are remembered better^59^, and environment boundaries have even been demonstrated to offset grid cell-based path integration error accumulation^60^. Despite this, we found that increasing error from start to end in the square, as expected by a path integration account, is overshadowed by a (disruptive) influence of the trapezoid boundaries. This effect was strongest at the narrow end, even though its boundaries were the most informative and its area of possible error was the smallest. This is an intriguing psychological dimension of our data. We speculate that because they are not symmetrical, the trapezoid boundaries’ appearance of offering greater information for self-localization (true to an extent because of the polarity) may draw participants to rely more on the boundaries in the trapezoid than they would in the square (where the symmetry dramatically reduces how informative the walls are about current heading or location). However, from both the grid-cell-based mechanistic framework and a cue-competition perspective, such reliance on both cue sources in the trapezoid instead of predominantly the distal ones would be to their detriment, resulting in larger and more geometrically aligned memory errors. Here, cue competition especially arises from the grid-cell-based framework: the precision/signaling of heading and location does not fully agree between cue sources if one source [geometry] distorts spatial perception. In fact, our SOD data suggest the degree of influence of this source of error on navigation may be an important individual difference.

We have consistently shown that SOD may not inherently predict better learning outcomes—rather, as a metacognitive measure that tracks confidence and individual encoding strategies in route learning. In so doing, it significantly moderates relationships between (a) spatial cue visibility and route learning processes and (b) memory load, interference, and configural learning outcomes of navigational experiences^13–17^. People also differ in the number of cues needed to succeed in spatial tasks, particularly when cues are ambiguous^61^. SOD may be a factor predicting whether configural cues can be detrimental or beneficial^13^. In general, high SOD is associated with greater cue integration tendencies, especially in environments with limited cues such as squares. Yet, it remains unknown how cues that have been shown to distort spatial memory and grid cell firing fields, such as trapezoidal boundaries, may relate to individual differences in SOD. Integrating distorting cues into cognitive maps may impair memory by disproportionately distorting neural coding of spatial metrics.

We observed that SOD had a simple relationship with better performance in the square where cue integration is more ambiguous, aligning with our predictions of SOD and performance in low cue availability. However, our trapezoid effects deviated from traditional understandings of SOD. High SOD individuals performed worse, on average, in the trapezoid overhead test, while low SOD individuals’ spatial memory did not differ between the shapes. Thus, we suggest that SOD’s association with performance and cue availability depends on the types of cues available. When cues distort internal neural metrics of space, enhanced integration of these cues into cognitive maps can impair spatial memory. Indeed, our distributions of participant placement errors in the trapezoid suggest that participant memories were increasingly anchored to the trapezoid boundaries, especially in the narrow end.

Collectively, our results show that the precision of spatial memories is influenced by path integration along a route, individual capacities for SOD, and the structural regularity of the environment in which the route memory is situated. In complex real-world environments, the regularity of environment geometry varies dramatically. This variation is highly relevant for human navigation, given that urban and human-made environments are increasingly ubiquitous and complex. From an evolutionary perspective, distinctive geometric patterns and complexities of environmental barriers would have been relatively rare “in the wild” for much of our species’ development. It is plausible that this long-term view of human cognitive development in an ecological/evolutionary context contributes to participants’ inaccuracies in perceiving and remembering depths, distances, and angles in the face of our manipulation.

Our study lays the foundation for future studies to further expand upon. Future research on the influence of environment scales, as well as the influence of varying geometries and geometric distinctiveness (e.g., from major to minor uncertainty levels: circular, rectangular, trapezoidal with varying wall lengths, and kite-shaped environments), will be essential. Additionally, from an ecological perspective, it will be of interest to explore how well-defined and contiguous boundaries influence our memory system (e.g., row of trees vs. brick wall; straight line of road vs. irregular edges of riverbank). Finally, there is a clear opportunity to bridge our work more closely with studies of how upbringings in more rural vs. urban settings influence spatial memory performance in different environments^62^. Taken together, by establishing how key factors of path integration, individual capacities, and environment geometries interact to modulate and predict spatial memory in naturalistic navigation, our findings provide a critical foundation for future navigation research.

## Methods

We recruited 77 adults from Georgia Institute of Technology and the surrounding Atlanta community to participate in this study (38 females indicated by self-report gender, age = 20.540 ± 2.9756). Our sample size was modeled after previous power analyses demonstrating that environment geometry has a modest effect (*d* = 0.4) on spatial memory and necessitates a sample size of at least 52 for a statistical power of 80% (alpha = 0.05, two-tailed test). The study protocol (#H23024), ethics, and consent form were approved by the Georgia Institute of Technology Institutional Review Board (IRB). Participants provided informed consent and signed the approved consent form before completing the task. Additionally, participants completed the SBSOD to assess general navigational abilities. Participants earned course credit or monetary compensation ($15/h) for their participation, and they completed the task in around 1.5 h. The investigators were not blinded to allocation during experiments and outcome assessment. One participant was excluded from the study analysis due to incomplete data on motion sickness, and another participant was excluded due to invalid data. In total, 75 participants entered into the final analysis. No gender based analyses were performed. It is not reasonably expected that our effects of interest are influenced by gender.

### Overview

In this study, we investigated how environment geometry, serial order effects, and individual differences interact to influence and predict spatial navigation. Participants completed a virtual, desktop computer navigation task in which they learned the locations of objects presented in a fixed sequence. The accuracy of their object memories was then assessed through a first-person and overhead perspective, and accuracy was quantified by the Euclidean distance between the true positions of the object and participants’ remembered positions (spatial memory error). Prior to the task, participants familiarized themselves with navigating in a practice environment. All analyses were performed using JASP classical linear mixed models (version: 0.18.3.0) (details in “Supplementary Discussion”). The design of the task and environments is described below.

### Environment design

**Fig. 6:**
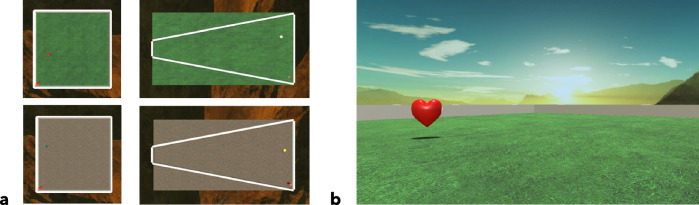
Overhead and first-person views of environment. **a** Overhead views of the shape of the square and trapezoid environments are displayed. Each participant completed the task in two square and two trapezoid environments. One pair of square and trapezoid had a grass ground, while the other pair had a dirt ground. **b** A first-person view of one of the environments is displayed. Mountains, clouds, and the sun were present in the background.

Participants completed an object-position memory task in which they learned the locations and sequence of six objects arranged in a successive route in two trapezoid environments and two square environments (Fig. 6a). The order of these four environments was counterbalanced and pseudorandomized to ensure a relatively equal distribution of environment order permutations. The boundary lengths of the trapezoid environment (36 × 76 × 8  × 76 vm) and square environment (40.27 × 40.27 vm) yielded matched surface areas to a design that was previously shown to be conducive to environment geometry effects in humans and grid cell distortions in rodents^10,12^. To facilitate orientation, distal cues were present in each environment. The distal cues consisted of mountains and clouds that surrounded the perimeter of the environment and a sun that, compared to the starting position, faced from the opposing end of the environment. The walls of the environment were of a uniform light gray color and smooth texture. For each participant, one pair of trapezoid and square environments had a green, grass floor, while the other pair of trapezoid and square environments had a brown, dirt floor; this controlled for a potential confound of ground color. While participants only completed the task in four environments (two trapezoids and two squares), there were eight environments in total used in the task. These eight environments consisted of two sets of four environments, each with two trapezoids and two squares with the properties described above. The two sets of four environments were identical, except for the locations of the objects in the routes. Each participant completed all four environments in one of these two sets. This feature of our design was intentional to control for the possibility of route difficulties that deviated significantly from normal. Approximately one-half of the participants completed the task in set one of four environments, Path Version A, while the remaining participants completed the task in the other set of four environments, Path Version B.

### Route design

Participants learned the locations of six objects within each environment; these objects were constructed by combining two sets of object triplets. One set of triplets appeared in each half of the environment. Environment halves were defined as the midpoint of the trapezoid’s long axis and the top and bottom halves of the square. By placing the object triplets in each half of the environment, we were able to directly compare spatial memory error between environment sub-compartments, particularly, the narrow and wide ends of the trapezoid. A computer algorithm with these parameters generated many possible arrangements of the six object locations for the task, from which we selected eight arrangements (four trapezoid and four square) for use. Previous research has demonstrated that these parameters are conducive to environment geometry effects^12^. The object positions for each triplet followed several constraints: (1) A minimum distance of 11 vm was between all object positions. (2) A minimum distance of at least 3 vm was between every object and the nearest boundary. (3) The distance between one pair of objects within the triplet was parallel to one of the bases of the trapezoid or one of the walls of the square. (4) The third object, which was not used in constraint three, was placed at an angle ranging from 90 to 120 degrees relative to the first two. (5) The connections between the objects formed an isosceles triangle.

We broadly constructed the routes in each environment to alternate objects between the two triplets of objects. In the square, the first object was selected from the triplet in the bottom half. In the trapezoid, the first object was selected from the triplet in the left half, the wide end. Trapezoid routes typically followed the pattern of odd-numbered objects being at the wide end and even-numbered objects at the narrow end. However, strictly alternating between routes would confound our ability to disentangle environmental geometry and serial-order effects. So, a select few connections in our routes contained two objects from the same triplet (details in Supplementary Figures).

### Practice environment

**Fig. 7:**
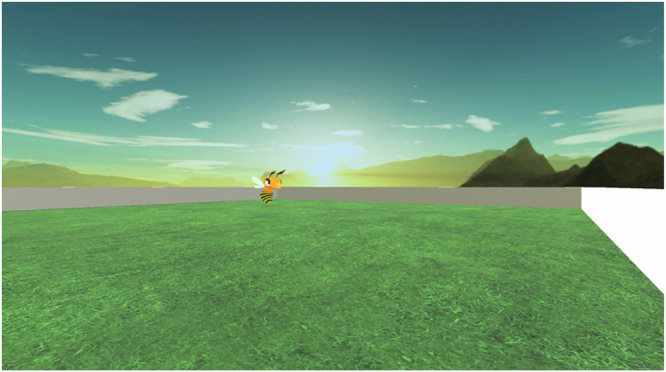
Practice environment. First-person view of the practice environment. Participants practiced navigating between objects in a square environment with a grassy ground. Mountains, clouds, and the sun were present in the background.

Participants practiced training in a virtual, desktop environment before completing the main task. The practice environment consisted of a square environment with the same characteristics as the main environments with respect to the internal and external cues (mountains, clouds, sun, and walls) (Fig. 7). The object identities and object positions used in the practice environment were unique to the practice environment. In total, participants practiced collecting objects along a route of six objects, similar to the main task. After the sixth object was collected, the practice environment was completed. Participants who needed further practice were able to repeat the practice environment as many times as needed.

### Main task

**Fig. 8:**
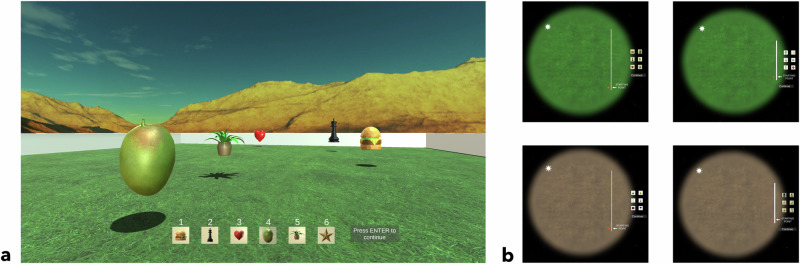
First-person and overhead tests. **a** An example image of the first-person test is displayed, demonstrating what it may look like once a participant has finished. **b** Example images of the overhead test are displayed. Participants completed the overhead test once for each environment. They did not see the shape of the environment in this task.

In the learning phase of the object-position memory task, participants began in the starting location of each environment (Fig. 6b). The first object in the route was immediately visible upon entering the environment. Participants were instructed to look for the object, walk up to it, and memorize its position and sequence in the route. Once participants walked to the object location, it would disappear, and the next object would appear. Participants were once again instructed to look for the object, walk up to it, and memorize its position and sequence. This procedure was repeated until the last (sixth) object of the environment was collected. After all objects were collected, participants were then tested on their memory for the six objects’ positions and sequence in a first-person test. Participants navigated the environment from a first-person perspective once again, where, importantly, all boundaries and distal cues were still present. They were instructed to place the same six objects that they had just encountered in the locations and sequence in which they remembered them. Each object had an icon with a number next to it displayed at the bottom of the screen, which indicated to the participant which number to press to drop the respective object. (Fig. 8a) The object icons were not ordered in the sequence in which participants encountered them. Participants had the opportunity to adjust their responses for an object at any time in the first-person test by moving to a new location and pressing a number key again. Objects remained visible in the environment once placed. In total, the learning of the six-object route and the subsequent first-person test of the objects concluded one trial of the object-position memory task. Participants completed three trials of the object-position memory task in each environment.

### Overhead test

The last task participants completed for an environment was an overhead test (Fig. 8b). The overhead test was an object placement task from a bird’s-eye view of the environment. Participants were given a map with the sun, the starting point, and the wall to the right of their starting position. Participants dragged object icons on the screen to where they believed they encountered them. Participants did not see the shape of the environment in this task. Instead, the overhead test was performed on a large, circular field with diffused edges. Only one boundary was present in the overhead test because we did not want to reveal the shape of the environments to participants while they completed the study, and we wanted to potentially constrain inter-object placement in a way that could mask how accurate/inaccurate the relative angles and distances between the objects were mapped in memory.

### Reporting summary

Further information on research design is available in the Nature Portfolio Reporting Summary linked to this article.

## Supplementary information

Supplementary Information

Reporting Summary

Transparent Peer Review file

## Data Availability

All display items presented in the main manuscript and supplementary information can be reproduced from material shared on GitHub (10.5281/zenodo.20045218^63^): github.com/MAPLabgroup/CURCI-G. The material in the repository includes all data, including processed and raw data, in addition to any programming scripts used.

## Code availability

This study used code for data organization, data analysis, and figure creation. All code and programming scripts that support the findings of this study are available on GitHub (10.5281/zenodo.20045218^63^): github.com/MAPLabgroup/CURCI-G.
