# Supplementary material for: Environment geometry alters sequential route learning and its integration into cognitive maps
Source: Nat Commun. 2026 Jul 7;17:8400. doi: 10.1038/s41467-026-75129-y (PMC13473551; doi:10.1038/s41467-026-75129-y)
Supplement: Supplementary file 1 [file 41467_2026_75129_MOESM1_ESM.pdf]

SUPPLEMENTARY DISCUSSION 2
Procrustes Analysis 2
Non-Global Methods 2
Non-Global Analysis 3
Environment Objects 4
Mixed-Effects Models 4
SUPPLEMENTARY FIGURES 5
Primary Dataset 5
Procrustes Analysis 10
Non-Global Methods 13
Non-Global Analysis 15
SUPPLEMENTARY TABLES 20
Primary Dataset 20
Procrustes Analysis 26
Non-Global Analysis 31

SUPPLEMENTARY DISCUSSION

Procrustes Analysis

Qualitatively, which effects were statistically significant or non-significant remained the same across our error measures of interest with the Procrustes transforms, including: effects of trapezoid subregion on spatial memory error, object serial order, object serial order * Shape, SOD, SOD*Shape, and matched-serial order objects in the square vs. the narrow end of the trapezoid. Exceptions to note were: 1. main effect of Shape across all participants (raw data: non-significant; vs. Procrustes data: marginally significant), suggesting that accounting for the referencing errors marginally increased sensitivity to an overall difference between squares and trapezoids, and 2. SOD*Shape interactions for individuals with high SOD in the overhead test (raw data: significant; vs. Procrustes data: marginally significant). Finally, when comparing matched-serial order objects in the square vs. the wide end of the trapezoid, the difference was non-significant for the Procrustes data, vs. in the raw data, spatial memory error in the wide end of the trapezoid error was significantly lower than the square. Given that most of our effects remained qualitatively the same, these analyses collectively suggest that our observed results are, largely, due to more focal errors in object-location memory and not due to overall referencing errors (see below for detailed statistics of the Procrustes control analyses).

With the Procrustes transform as a control, the interaction between object serial order and Shape remained significant (first-person: $F(5, 5140.14) = 3.265$, $p = .006$; overhead: $F(5, 1584.56) = 5.899$, $p < .001$) as well as the overall effect of object serial order (first-person: $F(5, 5140.14) = 15.065$, $p < .001$; overhead: $F(5, 1584.54) = 5.550$, $p < .001$) (Fig. S8). Spatial memory error was still significantly higher in the narrow end of the trapezoid than the wide end (first-person: $F(1, 2578.17) = 28.149$, $p < .001$; overhead: $F(1, 794.49) = 29.388$, $p < .001$) (Fig. S10). Comparing objects with matched object serial orders, spatial memory error was still significantly higher for objects in the narrow end of the trapezoid than the square (first-person: $F(1, 2470.22) = 18.023$, $p < .001$; overhead: $F(1, 722.12) = 27.947$, $p < .001$) (Fig. S11).

The overall influence of Shape (collapsing across the route), while not significant for the raw data, was marginally significant for the Procrustes-transformed data (first-person: $F(1, 796.12) = 3.752$, $p = .053$; overhead: $F(1, 213.29) = 3.855$, $p = .051$) (Fig. S7). Moreover, when comparing matched-serial-order objects in the square to those in the wide end of the trapezoid, the Procrustes-transformed data yielded a non-significant effect (first-person: $F(1, 2469.86) = .223$, $p = .637$; overhead: $F(1,761.79) = .022$, $p = .882$), while the raw data indicated significantly lower spatial memory error in the wide end of the trapezoid (Fig. S11). Analysis of this same effect on Procrustes-transformed data that does *not* permit scaling revealed a non-significant effect as well (first-person: $F(1, 2470.54) = 2.516$, $p = .113$; overhead: $F(1, 762.22) = .145$, $p = .703$) indicating that the difference was not due to scaling reference errors.

Finally, the main effect of SOD remained significant (first-person: $F(1, 73.38) = 5.779$, $p = .019$; overhead: $F(1, 73.94) = 4.721$, $p = .033$), as well as the SOD*Shape interaction in the overhead test ($F(1, 207.87) = 4.621$, $p = .033$) (Fig. S9). The SOD*Shape interaction in the first-person test remained non-significant ($F(1, 773.23) = .932$, $p = .335$). With the raw data, the SOD*Shape interaction in the overhead test was significant for high SOD individuals. This effect was marginal for the Procrustes-transformed data ($F(1, 103.08) = 3.766$, $p = .055$). For low SOD individuals, the SOD*Shape interaction remained null (see below).

Non-Global Methods

Our sample size was modeled after previous power analyses demonstrating that environment geometry has a modest effect ($d = .4$) on spatial memory and necessitates a sample size of at least 52 for a statistical power of 80% (alpha = 0.05, two-tailed test). 82 adults were recruited for the Non-Global condition (52 females indicated by self-report gender, age = 20.049 ± 2.233). Four participants were excluded from the Non-Global condition analysis due to invalid data. Here, I report the differences in methodology between the Global and Non-Global conditions. For any details not included below, they are the same between the Global and Non-Global conditions (see main text). Object identities, sequences, and locations were the same between conditions. The study protocol (#H23024), ethics, and consent form was approved by the Georgia Institute of Technology Institutional Review Board (IRB). Participants provided informed consent and signed the approved consent form before completing the task. No

gender based analyses were performed. It is not reasonably expected that our effects of interest are influenced by gender. Participants earned course credit or monetary compensation ($15/hr) for their participation, and they completed the task in around 1.5 hours. The study protocol (#H23024), ethics, and consent form was approved by the Georgia Institute of Technology Institutional Review Board (IRB) Participants provided informed consent and signed the approved consent form before completing the task.

Environment Design

In the Global condition, distal cues were present in each environment to facilitate orientation. The distal cues consisted of mountains and clouds that surrounded the perimeter of the environment and a sun that, compared to the starting position, faced from the opposing end of the environment (Fig. S13). The walls of the environments were of a uniform light gray color and smooth texture. In the Non-Global condition, no distal cues were present. Instead, each wall of the environment had a distinct color and texture, which facilitated orientation (Fig. S12). A practice environment consisted of a square environment with the same characteristics as the main square environment with respect to the condition of cue availability (Global vs. Non-Global) (Fig. S14) For the first-person tests, the only differences between Global & Non-Global conditions were the distal/local cues described before (Fig. S15).

Overhead Test

In each condition, participants saw the start point and the wall to the right of their start position. In the Global condition, participants also saw the relative location and direction of the sun. In the Non-Global condition, participants also saw icons indicating the wall colors of 1) the wall to the right of their start position, and 2) the wall directly opposite to the wall to the right of their start position (Fig. S15). Participants dragged object icons on the screen to where they remembered they encountered them. Participants did not see the complete shape of the environment in this task. Instead, the overhead test was performed on a large circular field with diffused edges.

Non-Global Analysis

Importantly, in qualitative terms, the majority of the results we report from the Global condition maintained the same pattern of statistical significance in this Non-Global condition for the first-person tests: Shape, trapezoid subregion, object serial order, object serial order * Shape, SOD, and comparing matched-serial order objects in the square vs. the narrow end of the trapezoid. Only one effect changed in significance: matched-serial order objects in the square vs. the wide end of the trapezoid, which showed significantly better performance in the trapezoid in our Global condition, did not differ between environment geometries in the Non-Global. This means that a facilitative effect of the wide end of the trapezoid compared to the square was present in the Global condition but not in the Non-Global condition (see below for details). This would be consistent with our environmental distinctiveness framework; since the square walls had different textures, they no longer offered impoverished orientation information compared to the wide end of the trapezoid. Taken together, our first-person navigation results generalized to conditions where all distal cues were removed and local environment boundary cues were modified to provide orientation information.

Similarly, for the overhead test, the pattern of statistical significance of the following effects remained the same as in our Global condition: trapezoid subregion, object serial order, object serial order * Shape, SOD, and comparing matched-serial order objects in the square vs. the wide end of the trapezoid. However: 1) The effect of Shape became significant in the Non-Global condition (reflecting better spatial memory overall in the trapezoid). 2) Shape*SOD was no longer significant in the Non-Global condition, and 3) There was no significant difference between the narrow end of the trapezoid and the square when comparing matched-serial order objects. Given the broad agreement between cohorts and environment designs for the first person test, but these different significance patterns in the overhead tests, we suggest the nature of the memory targeted in the overhead probes may be more sensitive to cue availability than active navigation / first person probes. Overall, this preliminary data strongly motivates future studies designed to test the difference between the probe reference frame and the sensitivity of memory distortions to environment cues more deeply.

Statistics Summary

For the following effects, the pattern of statistical significance remained the same in the Non-Global condition, for both first-person and overhead tests. The interaction between object serial order and Shape remained significant in the Non-Global condition (first-person: $F(5, 5323.08) = 4.640$, $p < .001$; overhead: $F(5, 1720.13) = 13.232$, $p < .001$), as well as the overall effect of object serial order (first-person: $F(5, 5323.08) = 30.851$, $p < .001$; overhead: $F(5, 1720.13) = 16.740$, $p < .001$) (Fig. S17). Spatial memory error was still significantly higher in the narrow end of the trapezoid than the wide end (first-person: $F(1, 2653.67) = 46.648$, $p < .001$; overhead: $F(1, 832.28) = 145.173$, $p < .001$) (Fig. S19).

In the first-person test, the significance of the following additional effects remained the same in the Non-Global condition: Shape ($F(1, 824.52) = .248$, $p = .618$) (Fig. S16), SOD ($F(1, 75.05) = 15.210$, $p < .001$) (Fig. S18), and comparing matched-serial order objects in the square vs. the narrow end of the trapezoid ($F(1, 2559.91) = 5.059$, $p = .025$) (Fig. S20). However, when comparing matched-serial order objects in the square vs. the wide end of the trapezoid, the result was nonsignificant in the Non-Global condition ($F(1, 2560.09) = 2.398$, $p = .122$), while it was significant in the Global condition (Fig. S20).

In the overhead test, Shape was significant in the Non-Global condition ($F(1, 224.13) = 11.256$, $p < .001$), while it was not in the Global condition (Fig. S16). Furthermore, SOD*Shape was no longer significant in the Non-Global condition ($F(1, 217.28) = .204$, $p = .652$), nor was the difference between the narrow end of the trapezoid and the square when comparing objects with matched serial orders ($F(1, 800.23) = .084$, $p = .771$).

Environment Objects

Square variant 1 (green floor): heart, star, burger, chesspiece, plant, mango

Square variant 2 (brown floor): plantpot, pear, tree, cherries, pencil, rubixcube

Trapezoid variant 1 (green floor): cupcake, peanut, dice, cup cactus, strawberry

Trapezoid variant 2 (brown floor): orange, flower, arcade, lemon, snowman, boat

Objects are listed in the serial order of the route. Note that the order of the environment variants was counterbalanced and pseudorandomized across participants. This information applies to both the Global & Non-Global task.

Mixed-Effects Models

All models: tested with Satterthwaite test method and type III sum of squares. Classical linear mixed models in JASP version 0.18.3.0 was used. The sole random effect is subject ID and the sole random component is the intercept.

SUPPLEMENTARY FIGURES

Primary Dataset

S1. Spatial Memory Error in Square vs. Trapezoid

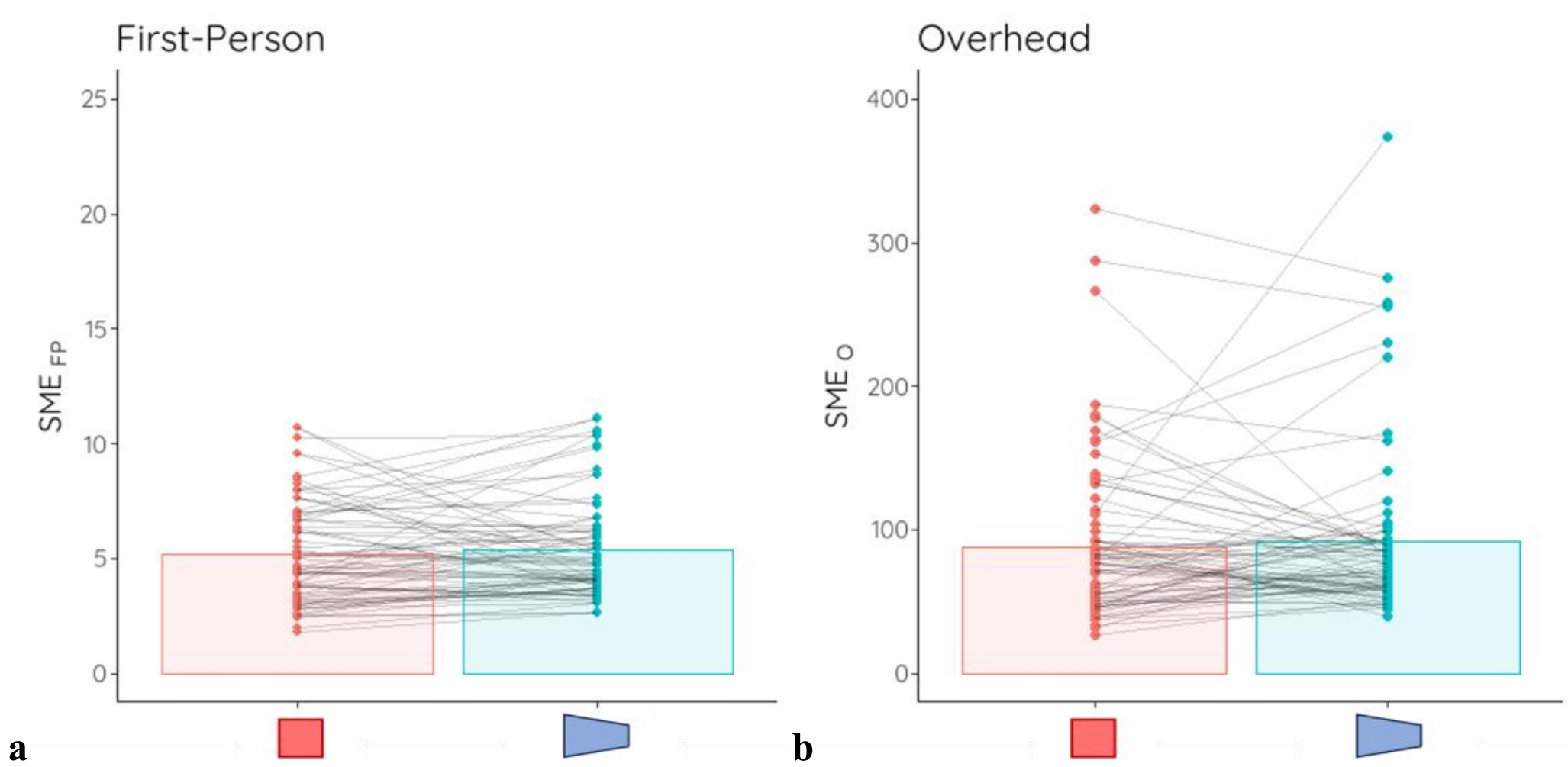

Supplementary Figure 1. **Spatial Memory Error in Square vs. Trapezoid.** Spatial memory error (SME) is plotted by Shape for each participant (n = 75) in **a** the first-person (FP) test, and **b** the overhead (O) test. Mixed-effects models revealed that Shape did not significantly modulate spatial memory error in either test. For visualization, each participant's data was averaged to one point per shape, and some outliers are excluded.

S2. Object Serial Order vs. Spatial Memory Error

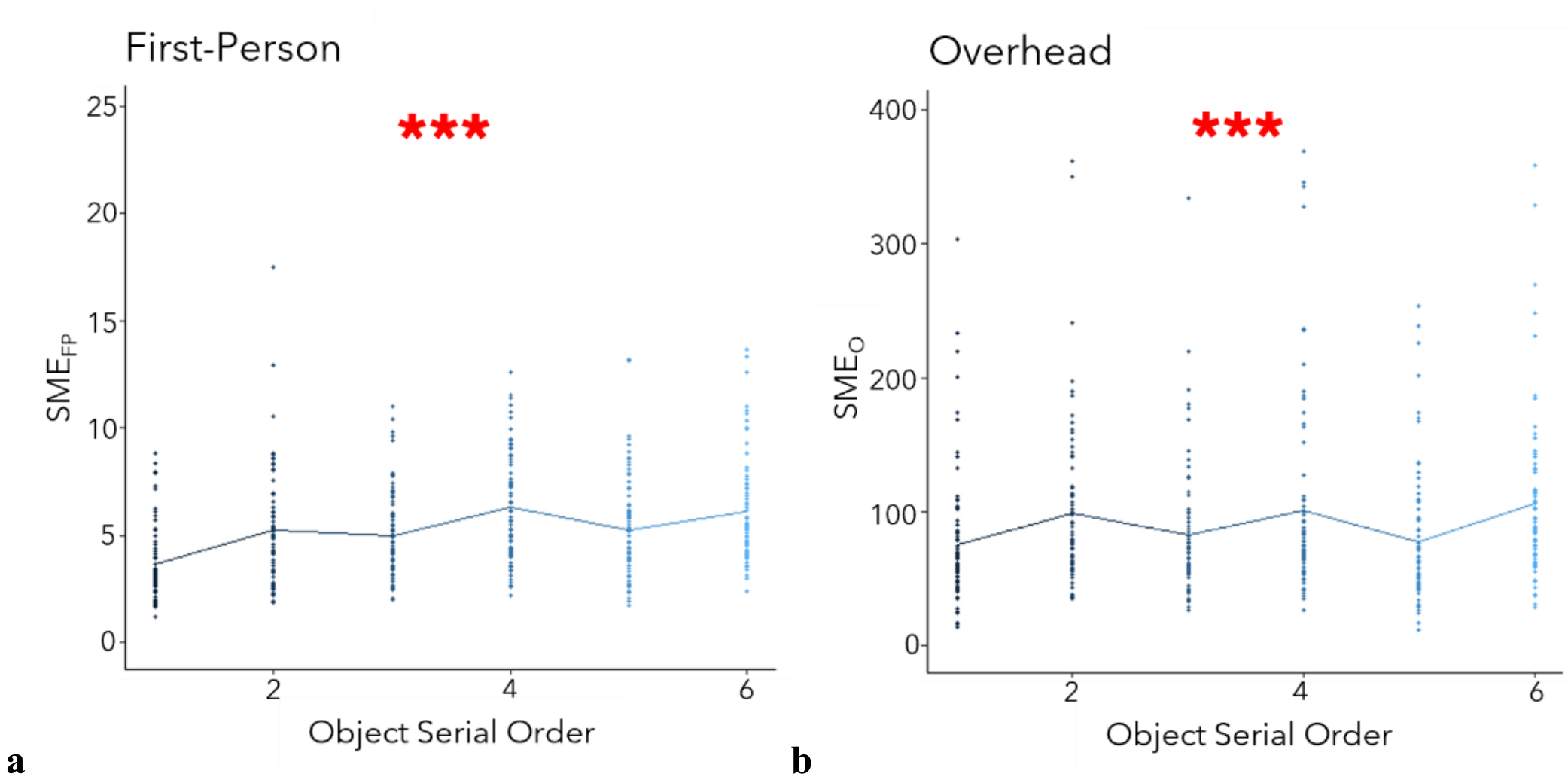

Supplementary Figure 2. **Object Serial Order vs. Spatial Memory Error.** Spatial memory error (SME) is plotted by the object order within the route for each participant (n = 75) in **a** the first-person (FP) test, and **b** the overhead test (O). Mixed effects models revealed a significant effect of object order (indicated by asterisks; $p < .001$ for both). For visualization, each participant's data was averaged to one data point per object, and some outliers were excluded.

S3. Sense of Direction vs. Spatial Memory Error.

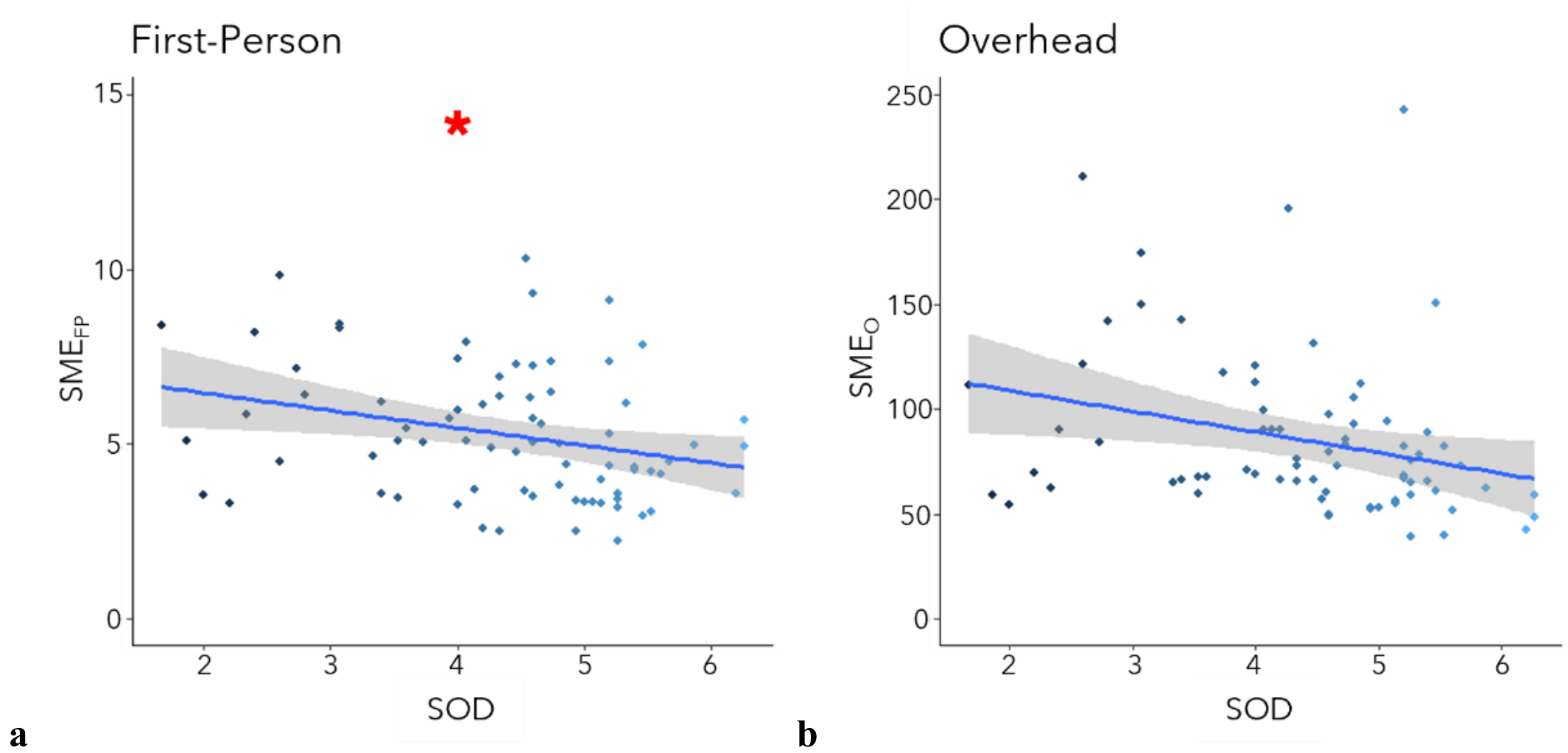

Supplementary Figure 3. **Sense of Direction vs. Spatial Memory Error.** Average spatial memory error (SME) is plotted by sense of direction (SOD) for each participant (n = 75) in **a** the first-person (FP) test, and **b** the overhead (O) test. Mixed-effects models revealed a significant SOD effect in the first-person test (p = .015), indicated by the asterisk. For visualization each participant is represented by a single point and some outliers are excluded. A trend line is displayed to visualize the model's fit of a linear relationship (gray shading represents error bounds of trend line).

S4. Environment Routes

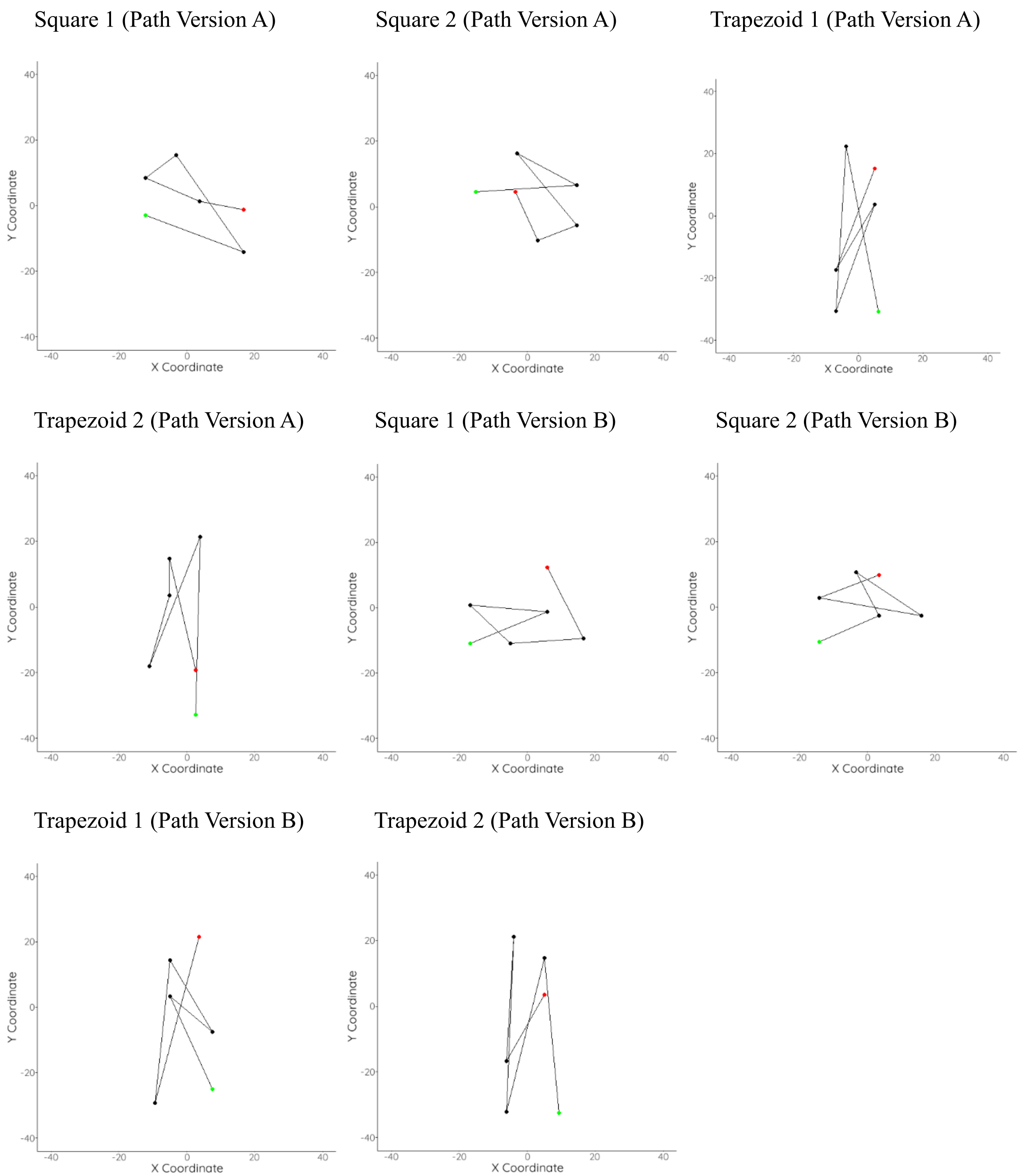

Supplementary Figure 4. **Environment Routes.** Each environment's routes are visualized above. Object locations are represented by dots, and the lines that connect them indicate the sequence of the object locations in the route. The first object is represented by the green dot, and the last object is represented by the red dot.

S5. First-Person Test Spatial Distribution Plots

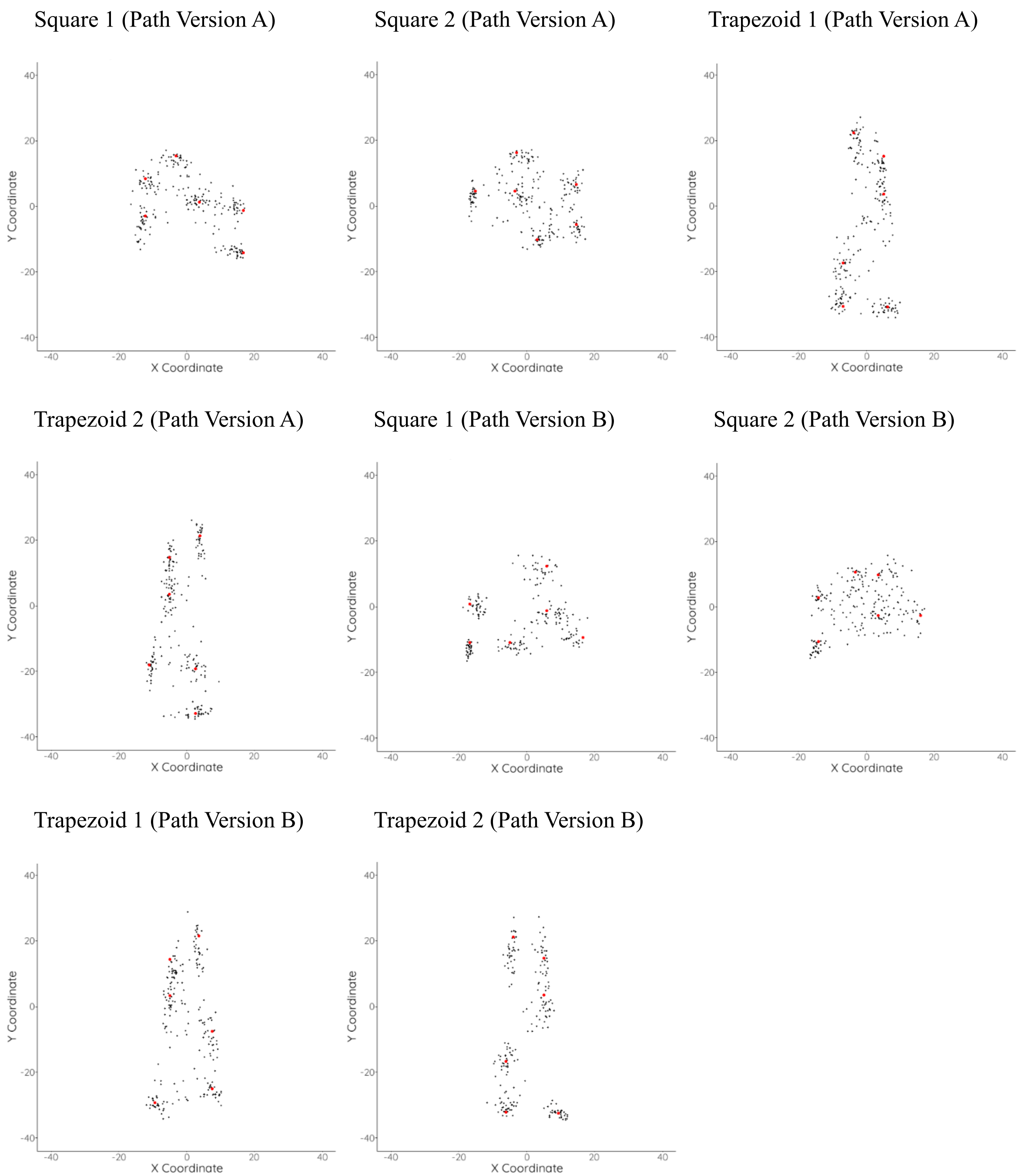

Supplementary Figure 5. **First-Person Test Spatial Distribution Plots.** Spatial distribution plots of participant responses are displayed for each environment above (n = 75 participants). Correct object locations are represented by red dots. Participant responses are represented by black dots. Within an environment, each participant's responses were averaged across trials to yield one dot per object (six total).

S6. Overhead Test Spatial Distribution Plots

Square 1 (Path Version A)

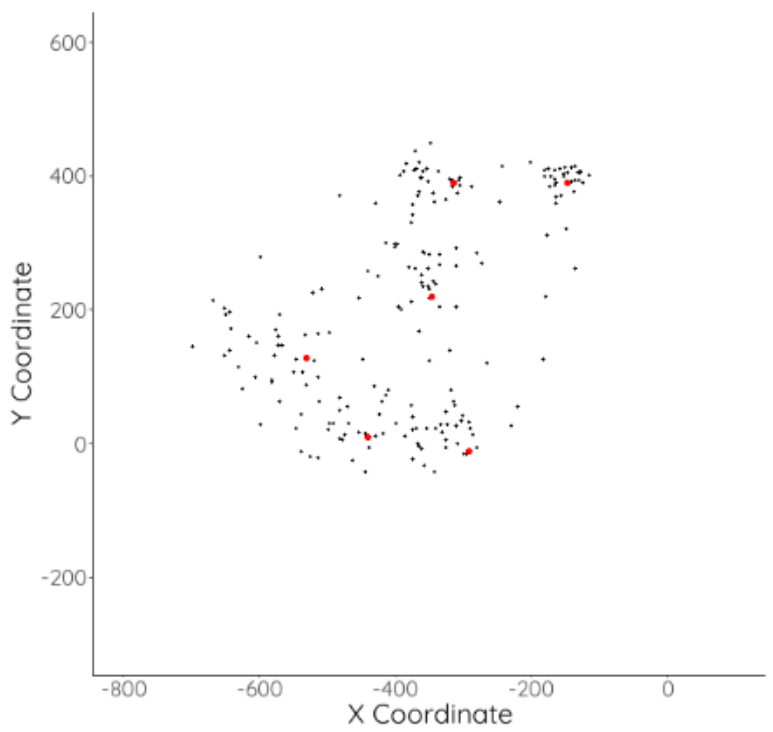

Square 2 (Path Version A)

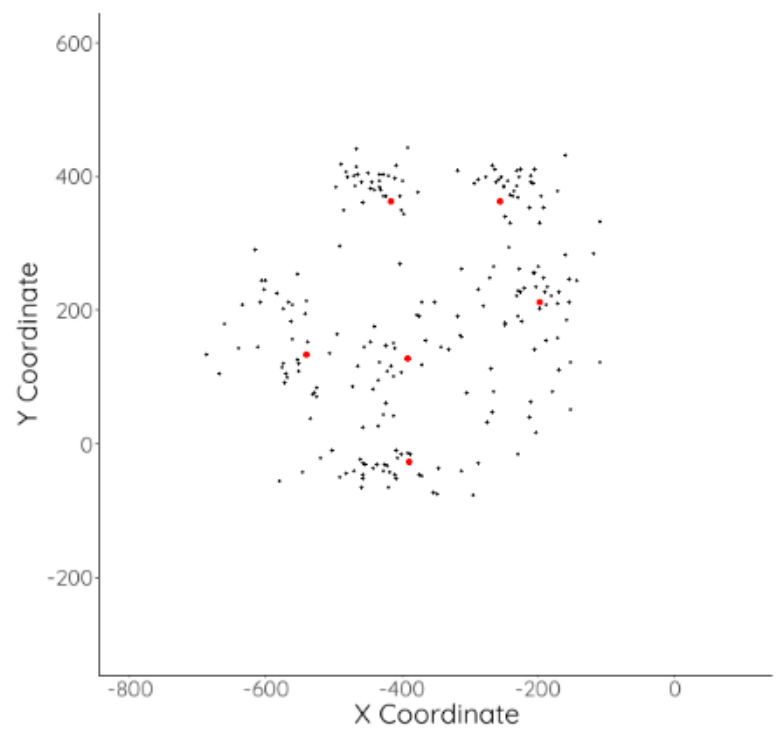

Trapezoid 1 (Path Version A)

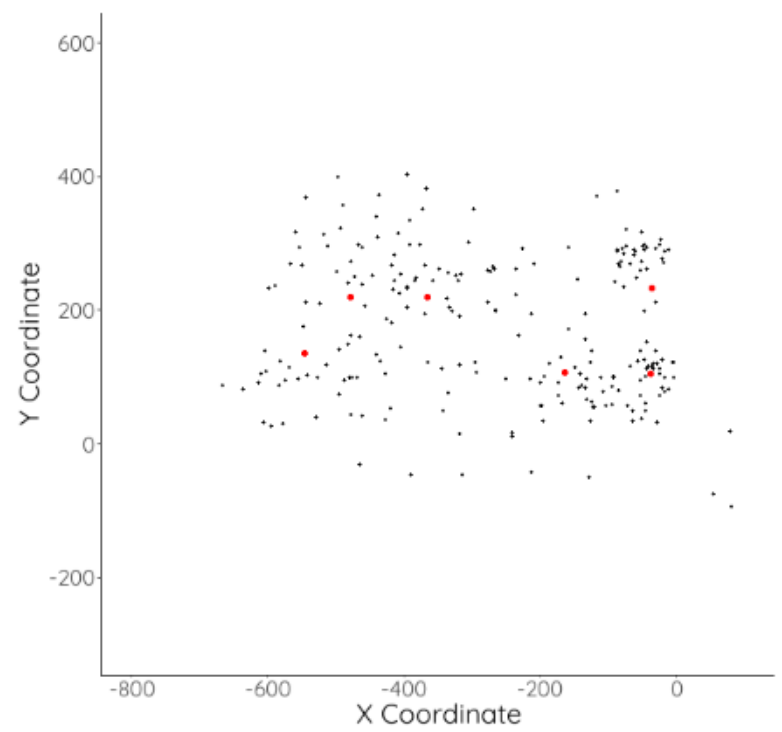

Trapezoid 2 (Path Version A)

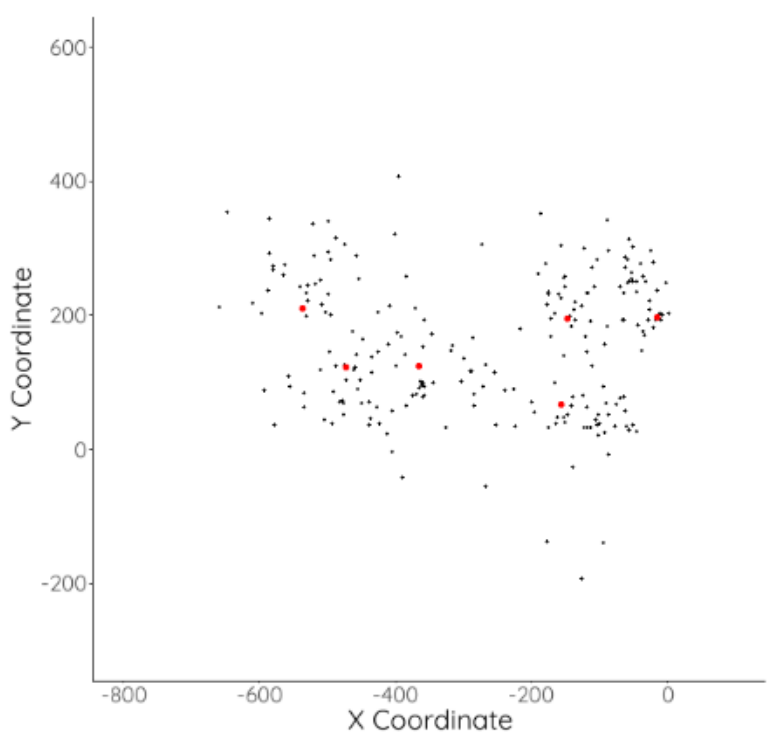

Square 1 (Path Version B)

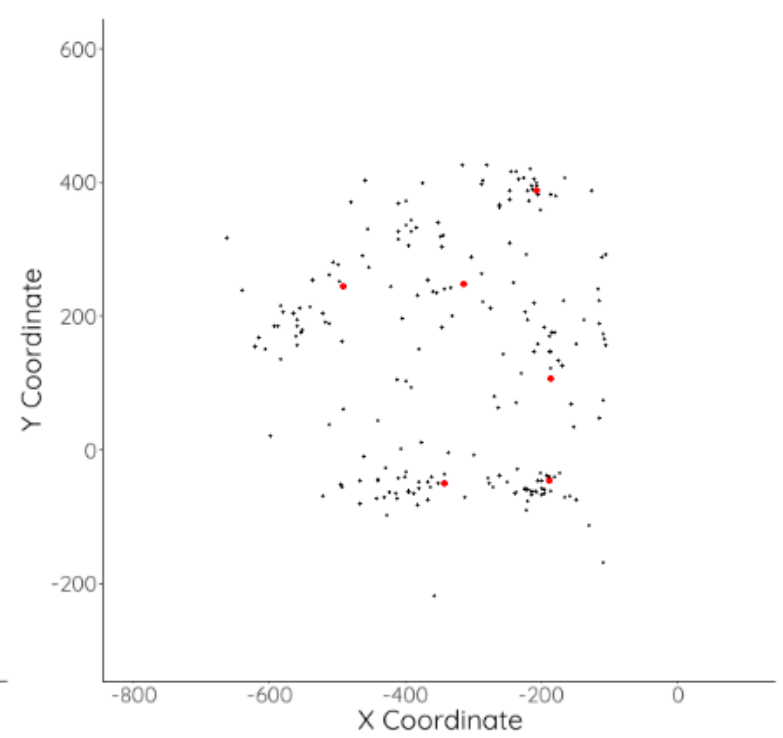

Square 2 (Path Version B)

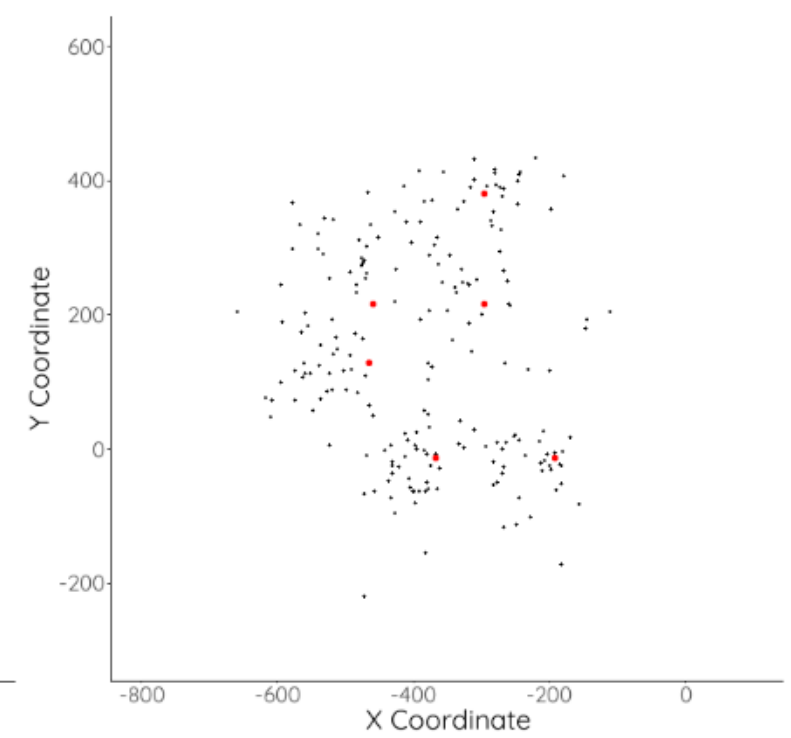

Trapezoid 1 (Path Version B)

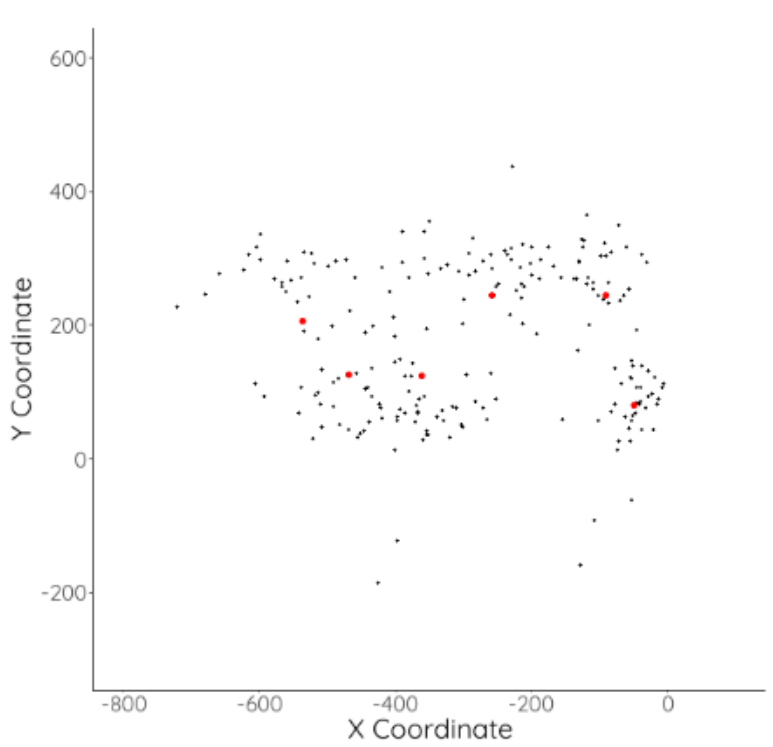

Trapezoid 2 (Path Version B)

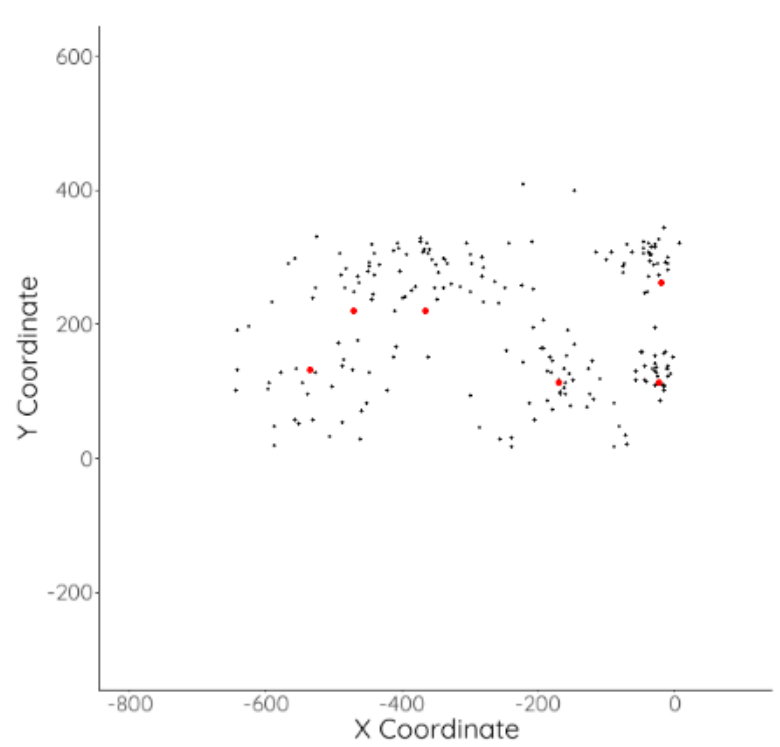

Supplementary Figure 6. **Overhead Test Spatial Distribution Plots.** Spatial distribution plots of participant responses are displayed for each environment above (n = 75 participants). Correct object locations are represented by red dots. Participant responses are represented by black dots (one point per each object in environment, six total).

Procrustes Analysis

S7. Spatial Memory Error in Square vs. Trapezoid - Procrustes

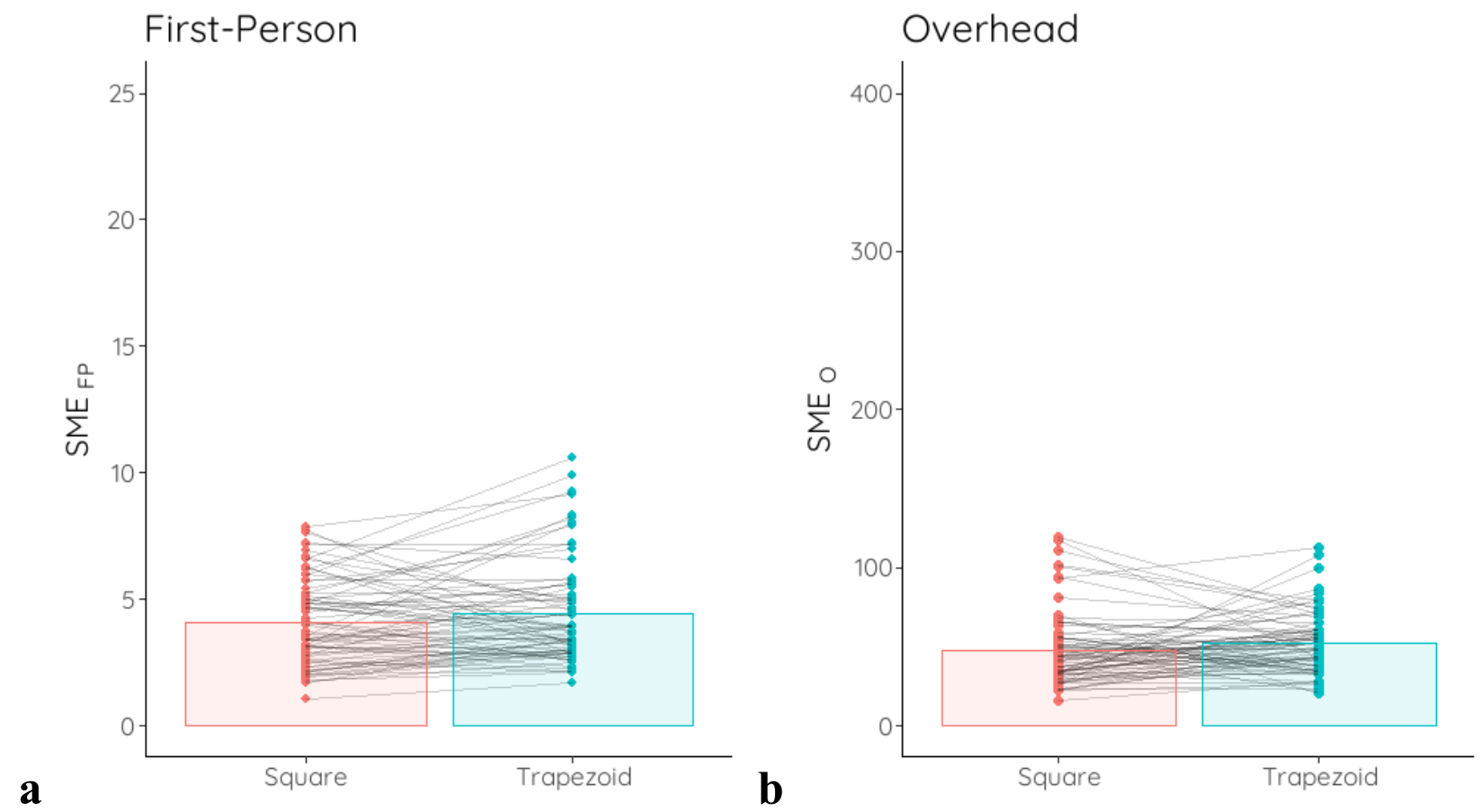

Supplementary Figure 7. **Spatial Memory Error in Square vs. Trapezoid - Procrustes.** Spatial memory error (SME) is plotted by Shape for each participant (n = 75) in **a** the first-person (FP) test, and **b** the overhead (O) test. Mixed-effects models revealed that Shape did not significantly modulate spatial memory error in either test. For visualization, each participant's data was averaged to one point per shape, and some outliers are excluded.

S8. Spatial Memory Error by Object Serial Order – Procrustes

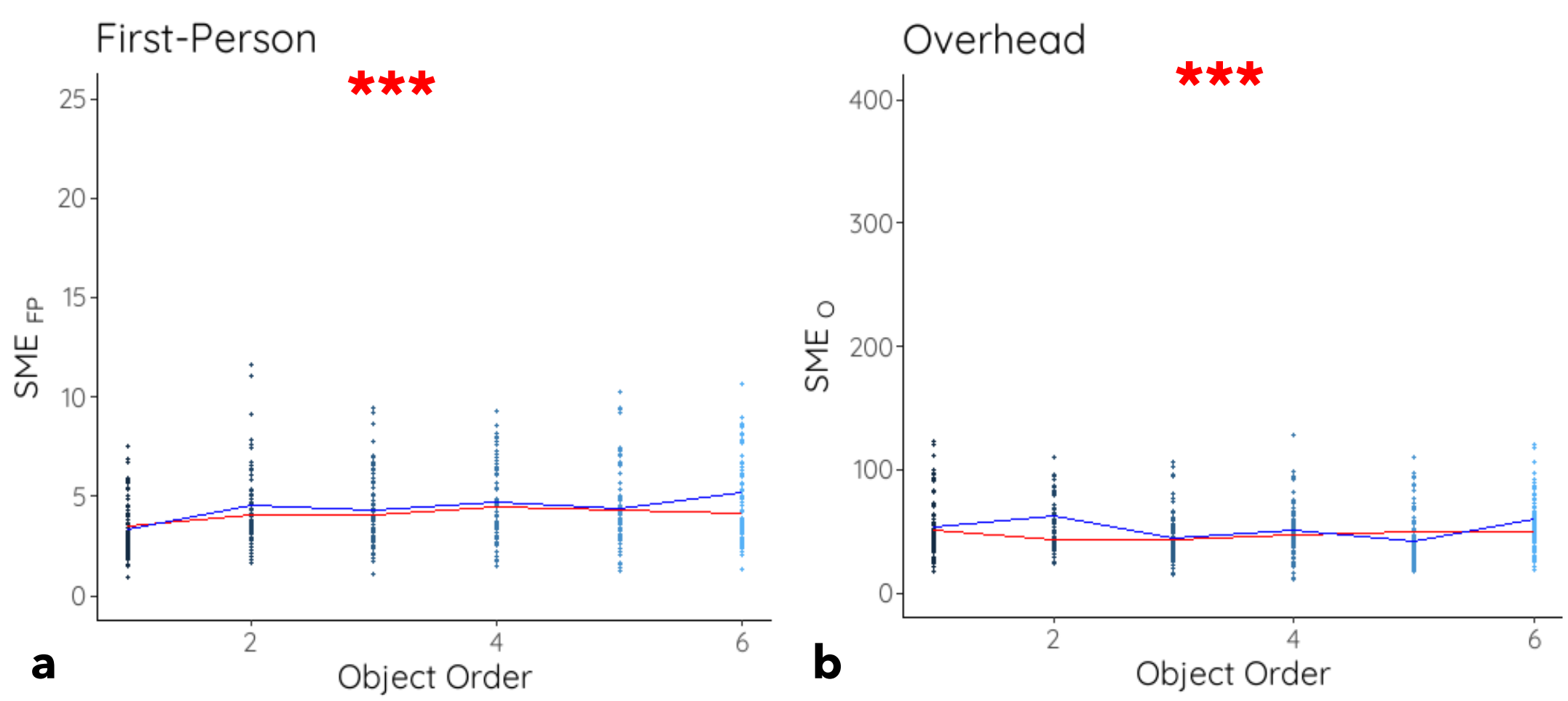

Supplementary Figure 8. **Spatial Memory Error by Object Serial Order - Procrustes.** Spatial memory error (SME) is plotted by the object order within the route for each participant (n = 75) in **a** the first-person (FP) test square (red line) and trapezoid (blue line), and **b** the overhead test (O) square (red line) and trapezoid (blue line). Mixed effects models revealed a significant effect of object order (indicated by asterisks; $p < .001$ for both). For visualization, each participant's data was averaged to one data point per object, and some outliers were excluded.

S9. Spatial Memory Error by Sense of Direction – Procrustes

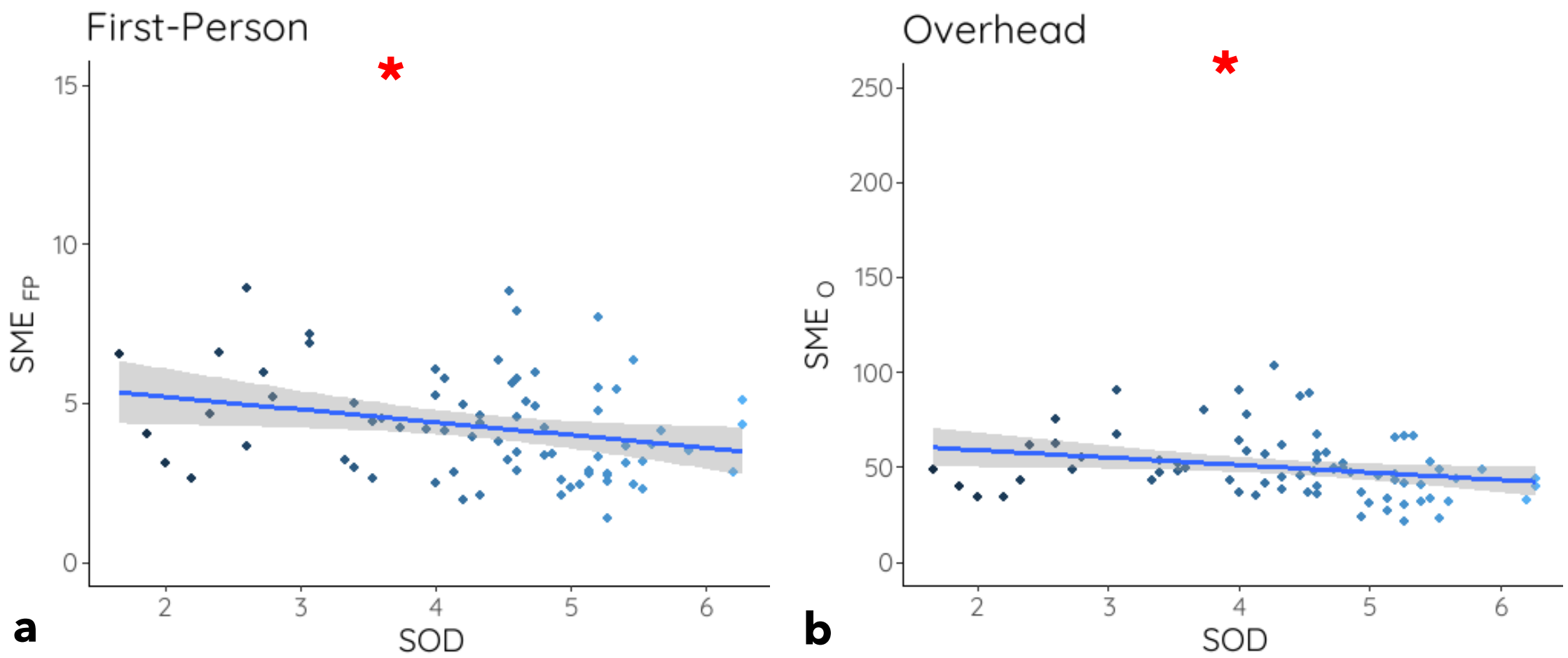

Supplementary Figure 9. **Sense of Direction vs. Spatial Memory Error - Procrustes.** Average spatial memory error (SME) is plotted by sense of direction (SOD) for each participant (n = 75) in **a** the first-person (FP) test, and **b** the overhead (O) test. Mixed-effects models revealed a significant SOD effect in the first-person test (p = .019) and overhead test (p = .033), indicated by the asterisks. For visualization each participant is represented by a single point and some outliers are excluded. A trend line is displayed to visualize the model's fit of a linear relationship (gray shading represents error bounds of trend line).

S10. Spatial Memory Error by Trapezoid Subregion- Procrustes

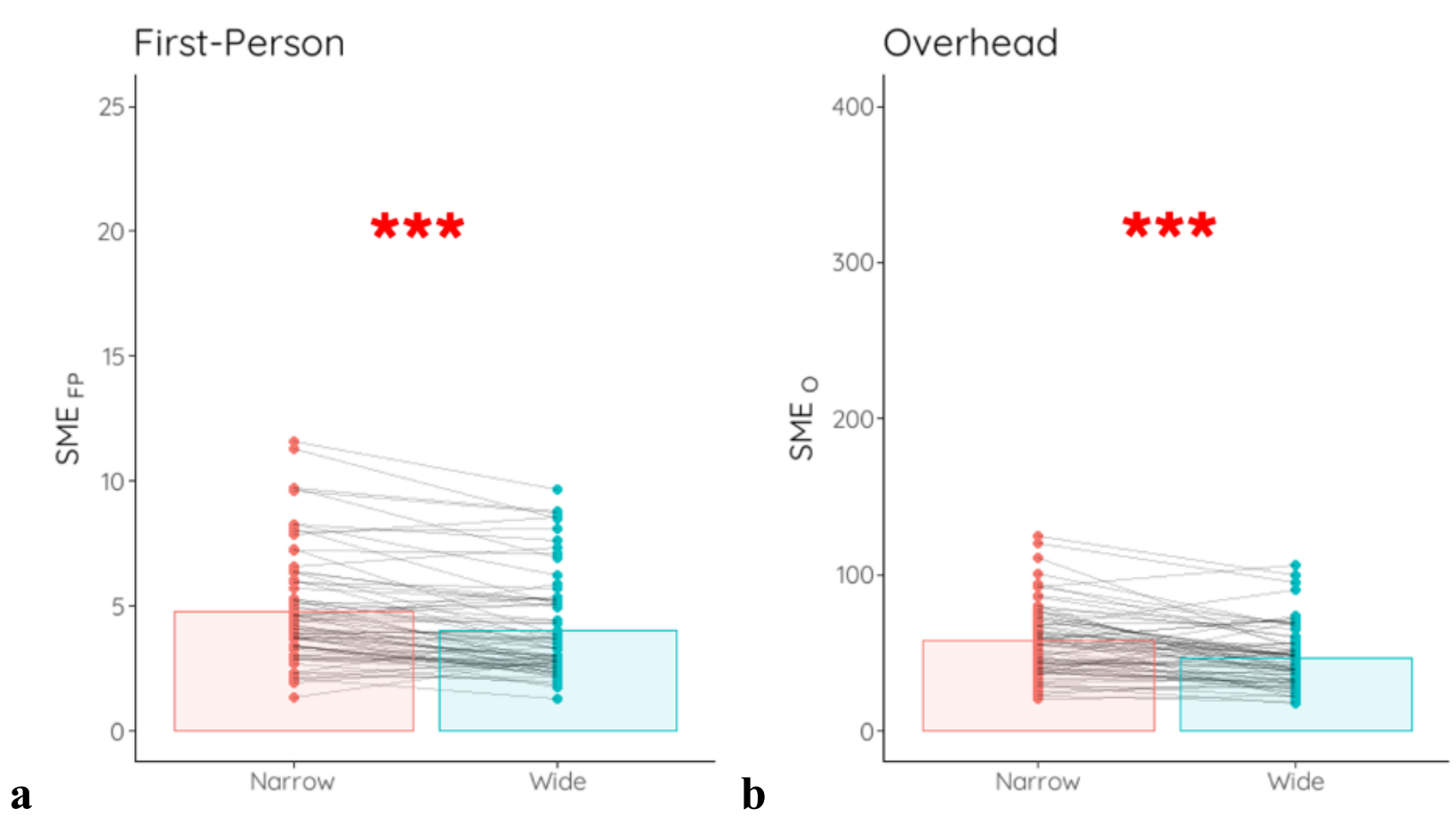

Supplementary Figure 10. **Spatial Memory Error by Trapezoid Subregion - Procrustes.** Spatial memory error (SME) is plotted by the narrow and wide halves of the trapezoid for each participant (n = 75) in **a** the first-person (FP) test, and **b** the overhead (O) test. Mixed-effects models revealed a significant effect of trapezoid subregion as indicated by the asterisks (p < .001 for both). For visualization, each participant's data was averaged to one point per trapezoid subregion, and some outliers were excluded.

S11. Trapezoid Subregion vs. Square – Procrustes

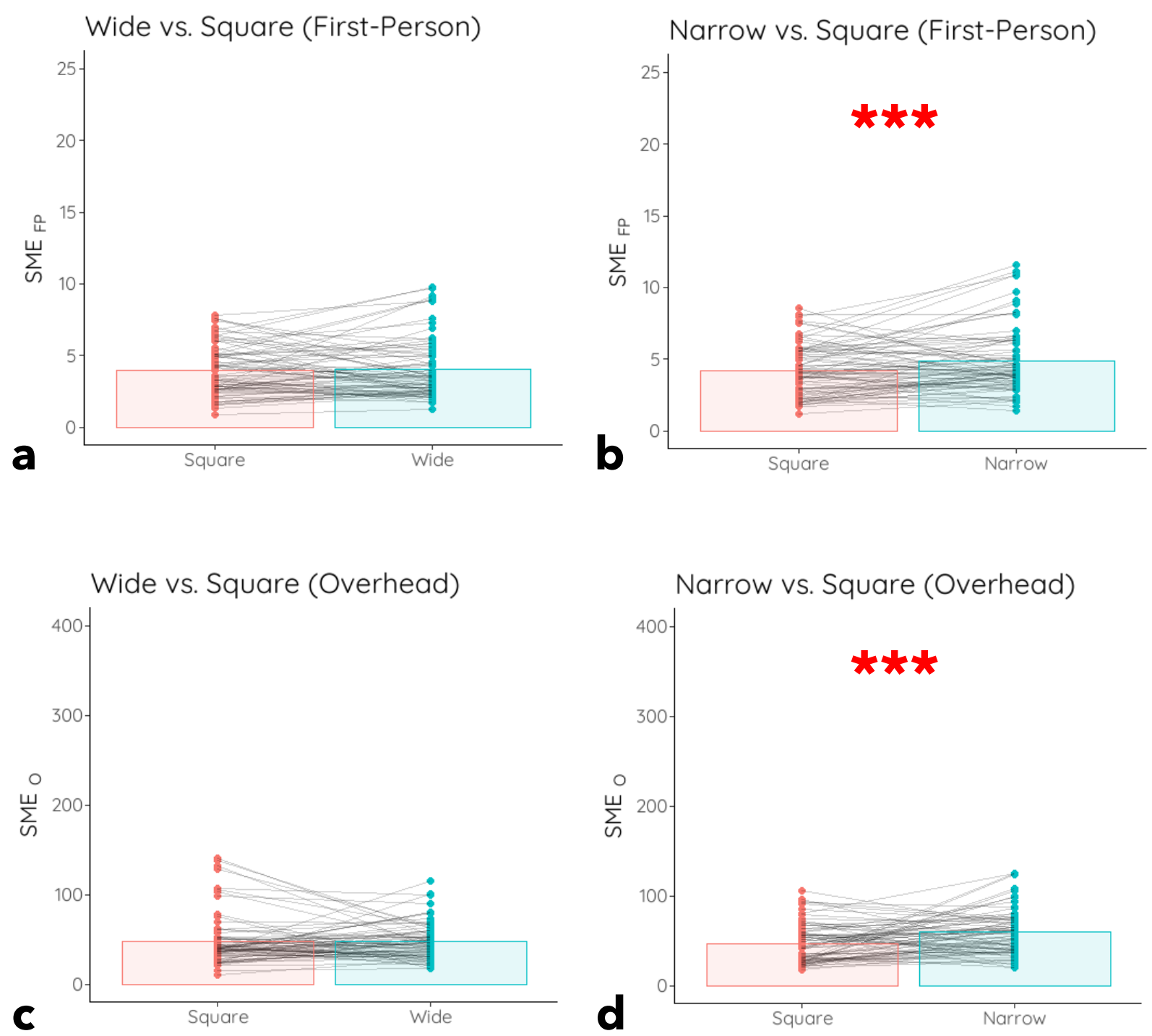

Supplementary Figure 11. **Trapezoid Subregion vs. Square - Procrustes.** Spatial memory error (SME) is plotted by Shape for **a** wide vs. square in the first-person (FP) test **b** narrow vs. square in the first-person test **c** wide vs. square in the overhead (O) test and **d** narrow vs. square in the overhead test (n = 75 participants). Mixed-effects models revealed the effect of Shape (trapezoid subregion vs. square) for **a** & **d** was significant as indicated by the asterisks ($p < .001$ for both). For visualization, each participant's data was averaged to one point per shape/subregion in each plot, and some outliers were excluded.

Non-Global Methods

S12. Overhead Views of Global & Non-Global Environments

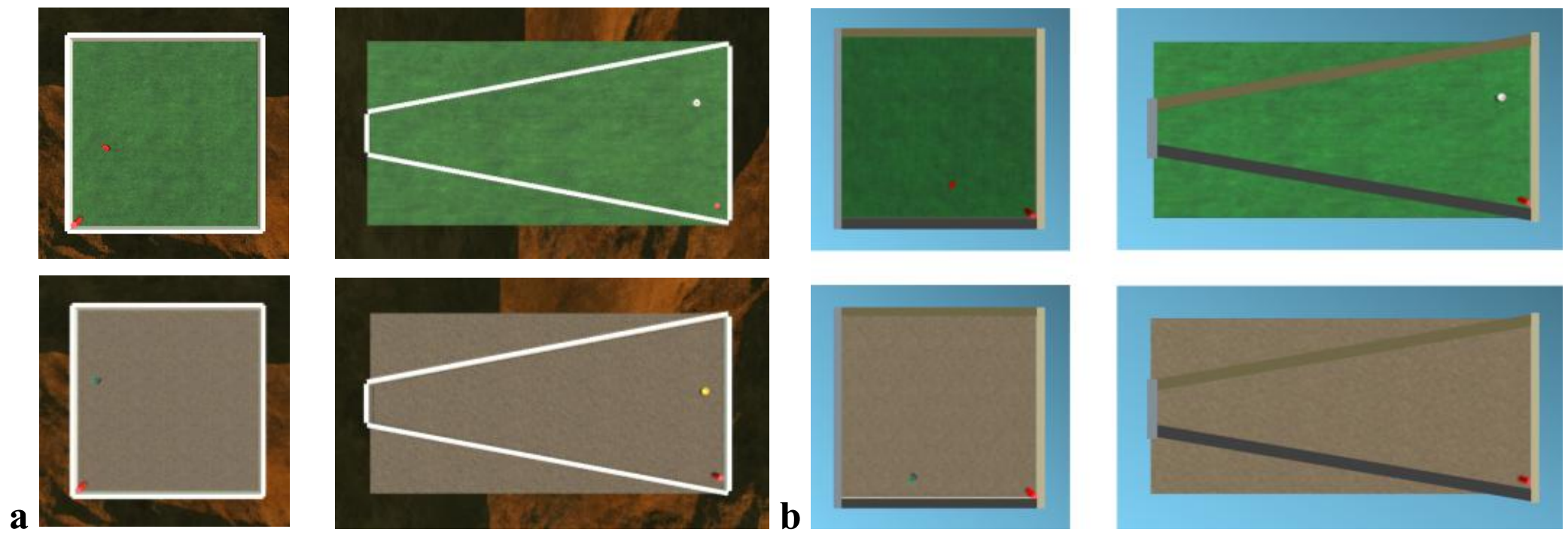

Supplementary Figure 12. **Overhead Views of Global & Non-Global Environments.** Overhead views of the square and trapezoid environments are displayed for **a** Global & **b** Non-Global. Each participant completed the task in two square and two trapezoid environments. Starting locations are marked by a red dot in the lower right corner. One pair of square and trapezoid had a grass ground, while the other pair had a dirt ground.

S13. First-Person View of Main Task Global & Non-Global Environments

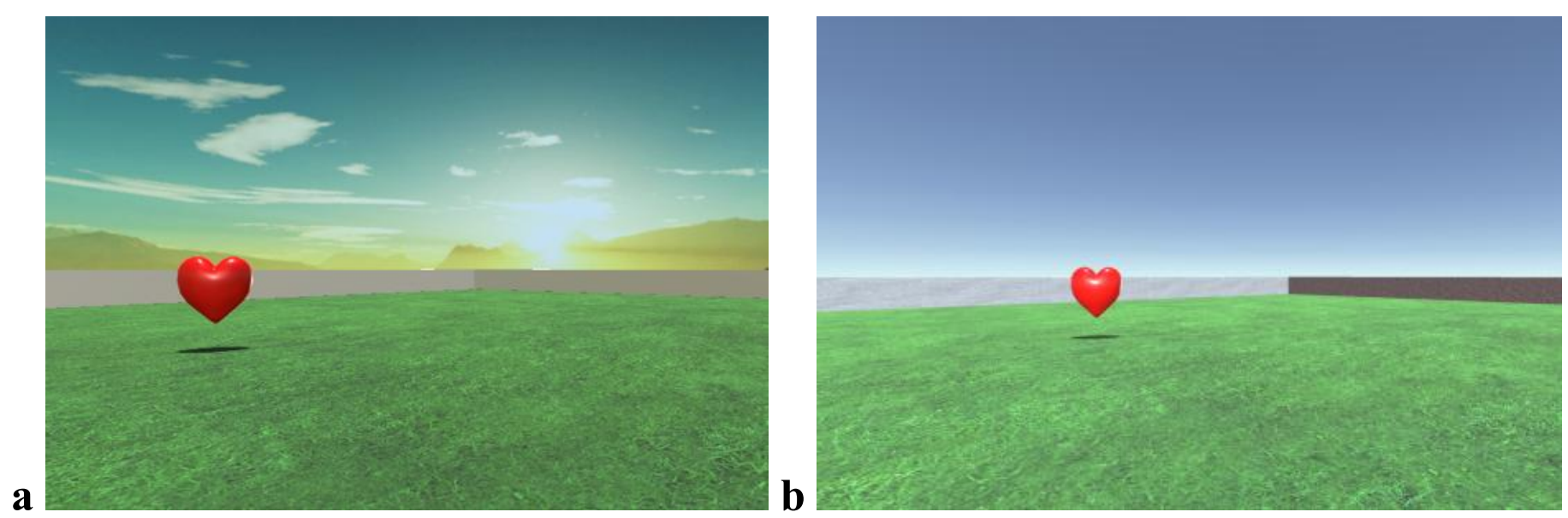

Supplementary Figure 13. **First-Person View of a Main Task Global & Non-Global Environment.** One of the environments is displayed for **a** Global & **b** Non-Global, demonstrating what it may look like upon first entering an environment.

S14. Practice Global & Non-Global Environments

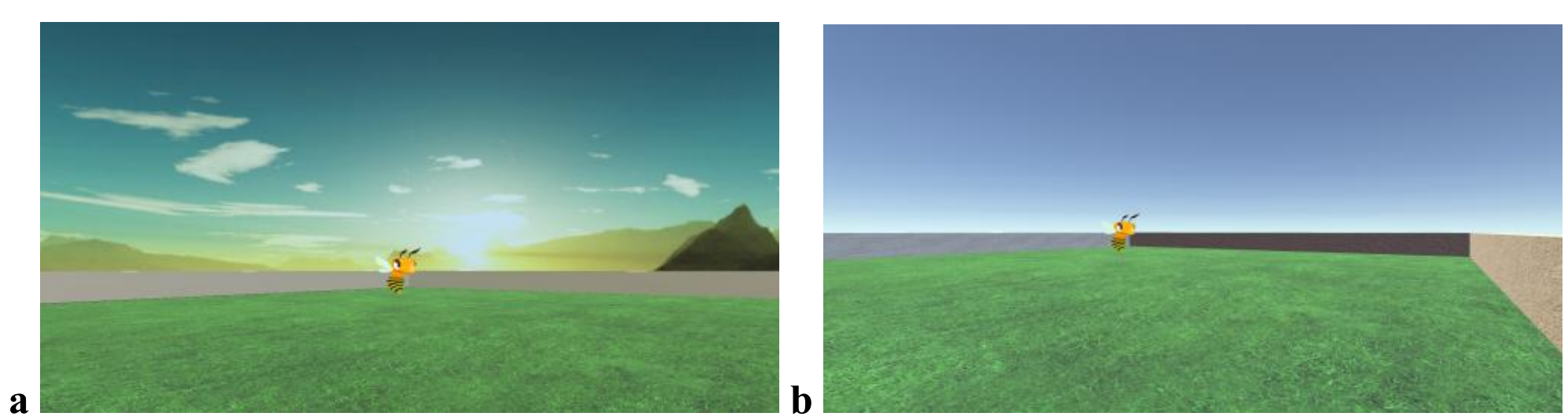

Supplementary Figure 14. **Practice Global & Non-Global Environments.** First-person view of the practice environment for a) Global & b) Non-Global. Participants practiced navigating between objects in a square environment with a grass ground.

S15. Example First-Person and Overhead Tests by Condition (Global & Non-Global)

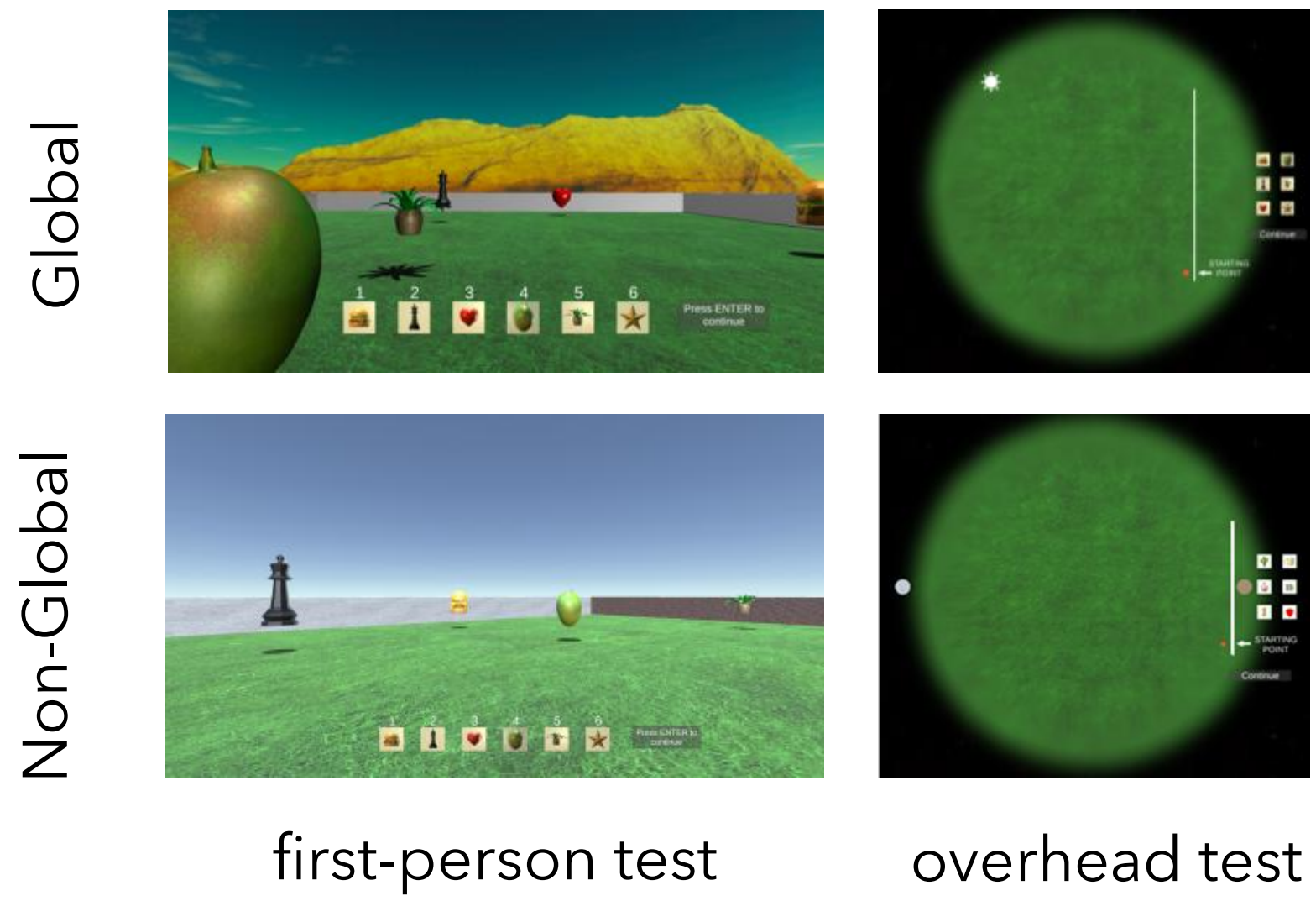

Supplementary Figure 15. **Example First-Person and Overhead Tests by Condition (Global & Non-Global).** Example images are displayed of the first-person test and overhead test for each condition of cue availability (Global & Non-Global).

Non-Global Analysis

S16. Spatial Memory Error in Square vs. Trapezoid – Non-Global Data

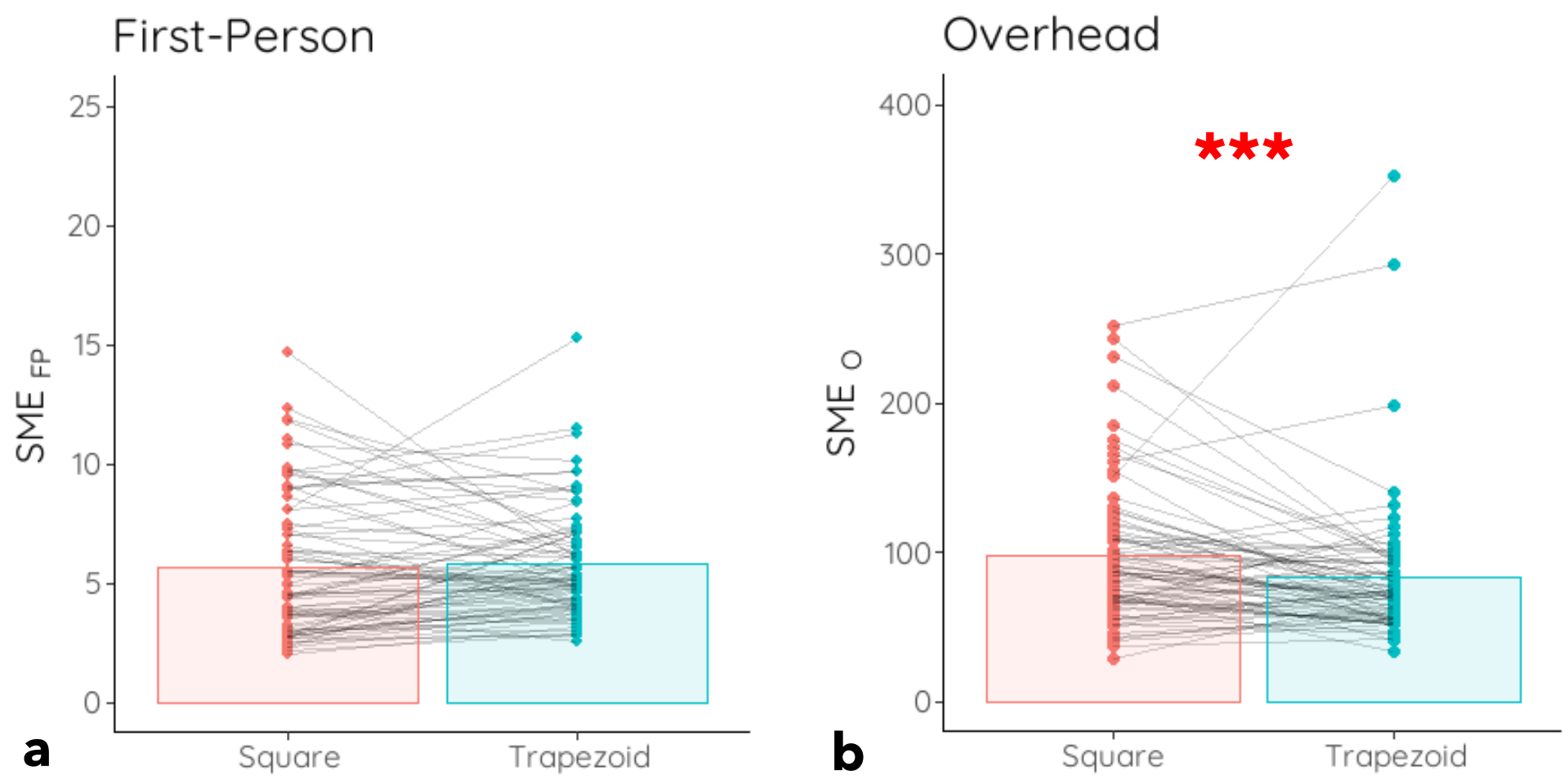

Supplementary Figure 16. **Spatial Memory Error in Square vs. Trapezoid – Non-Global Data.** Spatial memory error (SME) is plotted by Shape for each participant (n = 78) in **a** the first-person (FP) test, and **b** the overhead (O) test. Mixed-effects models revealed Shape significantly modulated spatial memory error in the overhead test ($p < .001$). For visualization, each participant's data was averaged to one point per shape, and some outliers are excluded.

S17. Spatial Memory Error by Object Serial Order – Non-Global Data

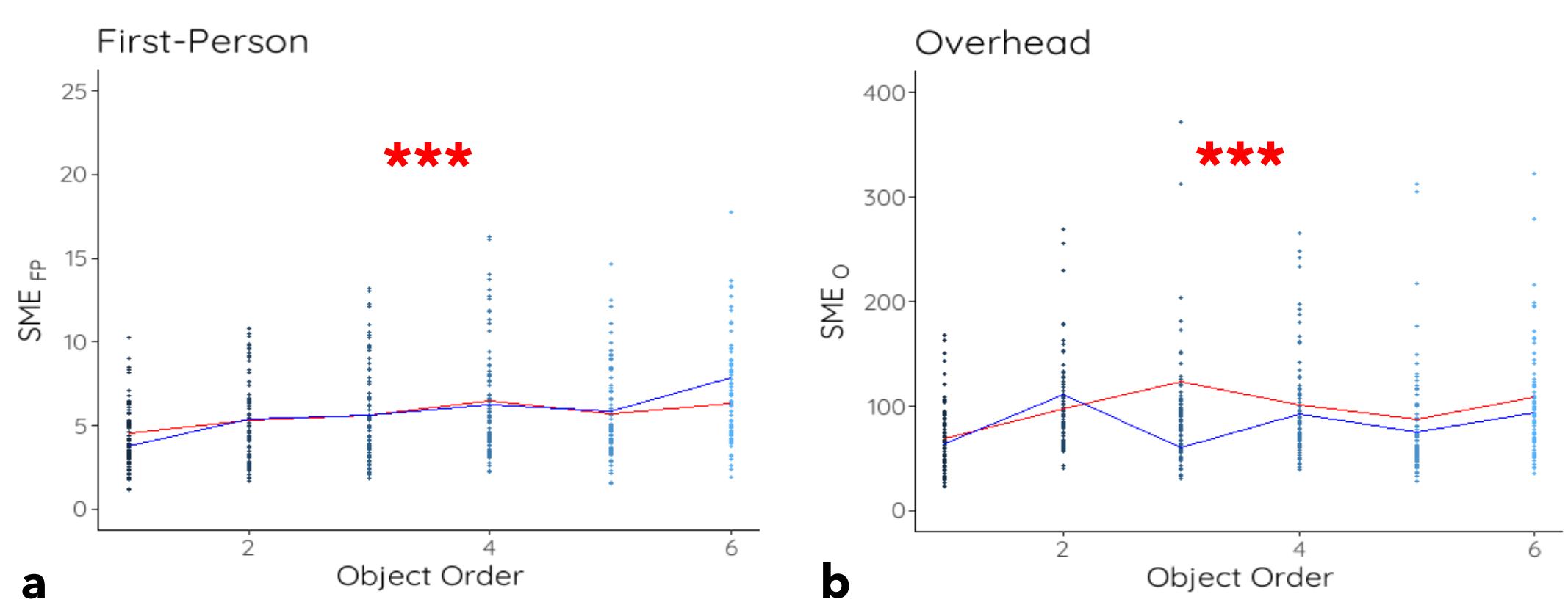

Supplementary Figure 17. **Spatial Memory Error by Object Serial Order – Non-Global Data.** Spatial memory error (SME) is plotted by the object order within the route for each participant (n = 78) in **a** the first-person (FP) test square (red line) and trapezoid (blue line), and **b** the overhead test (O) square (red line) and trapezoid (blue line). Mixed effects models revealed a significant effect of object order (indicated by asterisks; $p < .001$ for both). For visualization, each participant's data was averaged to one data point per object, and some outliers were excluded.

S18. Spatial Memory Error by Sense of Direction – Non-Global Data

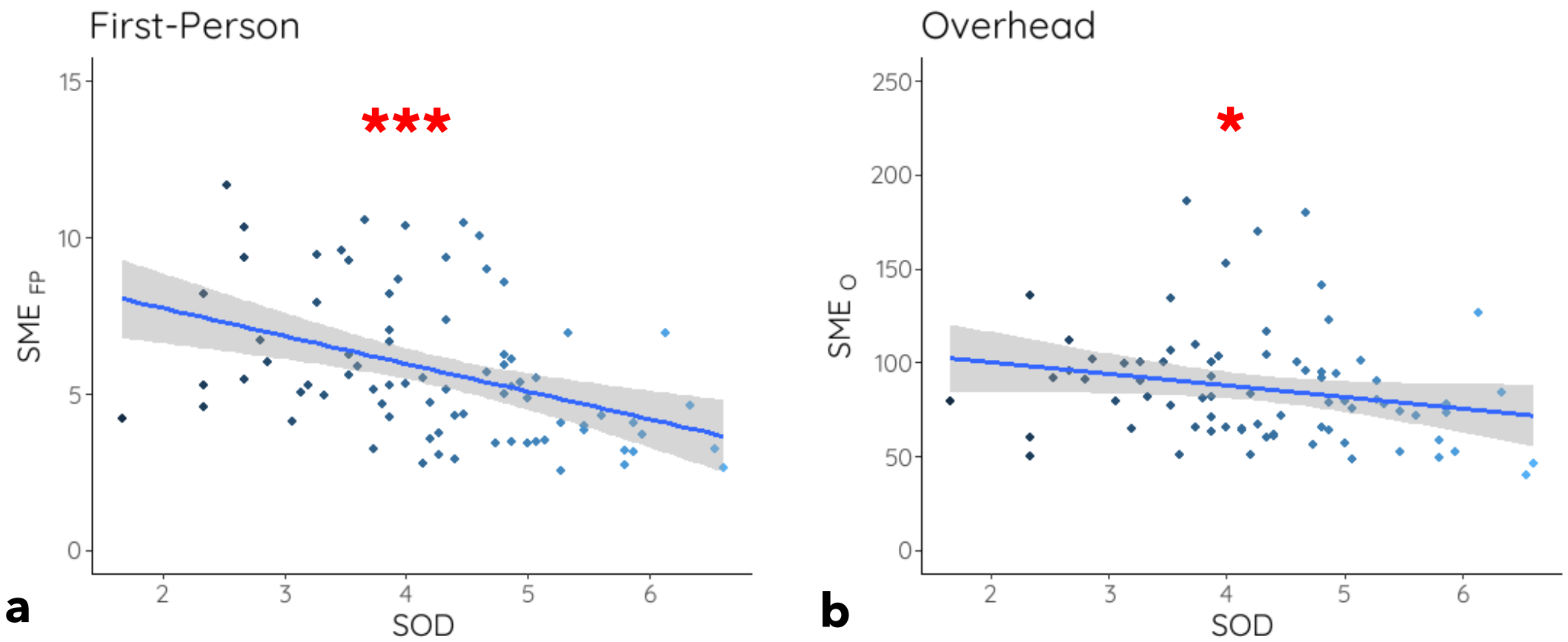

Supplementary Figure 18. **Sense of Direction vs. Spatial Memory Error – Non-Global Data.** Average spatial memory error (SME) is plotted by sense of direction (SOD) for each participant (n = 78) in **a** the first-person (FP) test, and **b** the overhead (O) test. Mixed-effects models revealed a significant SOD effect in the first-person test ($p < .001$) and overhead test ($p = .047$), indicated by the asterisks. For visualization each participant is represented by a single point and some outliers are excluded. A trend line is displayed to visualize the model's fit of a linear relationship (gray shading represents error bounds of trend line).

S19. Spatial Memory Error by Trapezoid Subregion – Non-Global Data

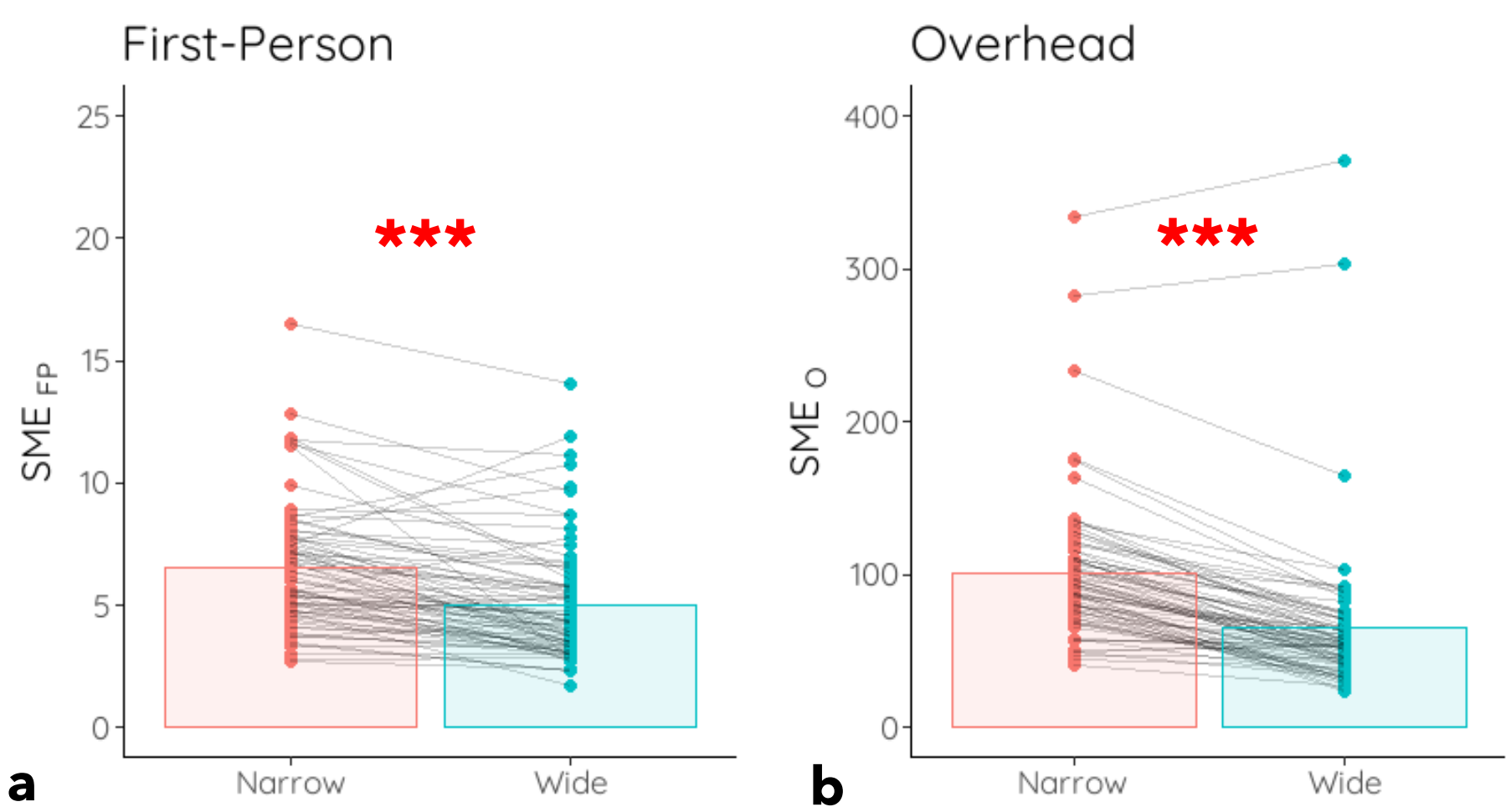

Supplementary Figure 19. **Spatial Memory Error by Trapezoid Subregion – Non-Global Data.** Spatial memory error (SME) is plotted by the narrow and wide halves of the trapezoid for each participant (n = 78) in **a** the first-person (FP) test, and **b** the overhead (O) test. Mixed-effects models revealed a significant effect of trapezoid subregion as indicated by the asterisks ($p < .001$ for both). For visualization, each participant's data was averaged to one point per trapezoid subregion, and some outliers were excluded.

S20. Trapezoid Subregion vs. Square – Non-Global Data

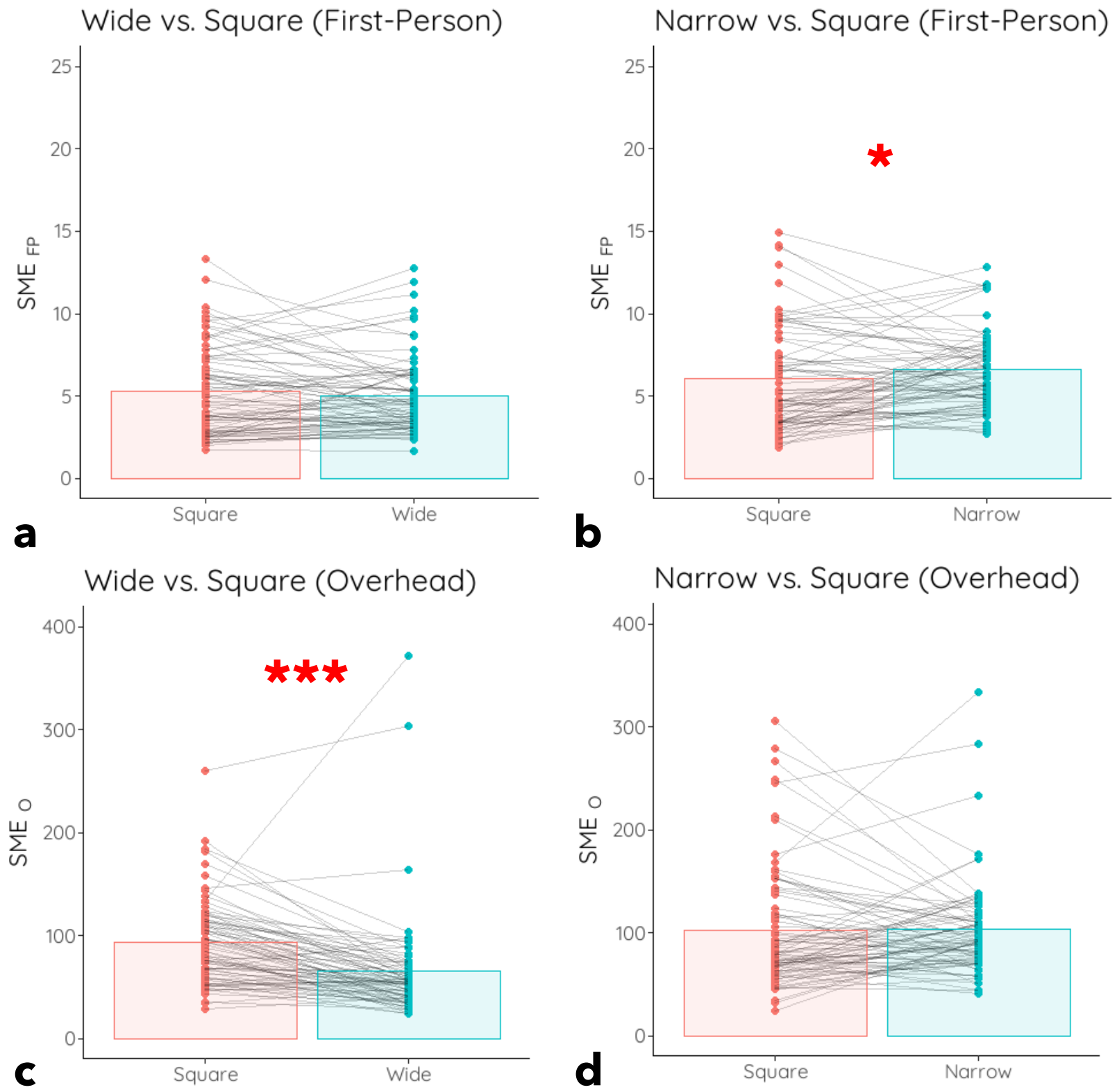

Supplementary Figure 20. **Trapezoid Subregion vs. Square – Non-Global Data.** Spatial memory error (SME) is plotted by Shape for **a** wide vs. square in the first-person (FP) test **b** narrow vs. square in the first-person test **c** wide vs. square in the overhead (O) test and **d** narrow vs. square in the overhead test (n = 78 participants). Mixed effects models revealed the effect of Shape (trapezoid subregion vs. square) for was significant for **b** (p = .025) and **c** (p < .001) as indicated by the asterisks. For visualization, each participant's data was averaged to one point per shape/subregion in each plot, and some outliers were excluded.

S21. First-Person Test Spatial Distribution Plots – Non-Global

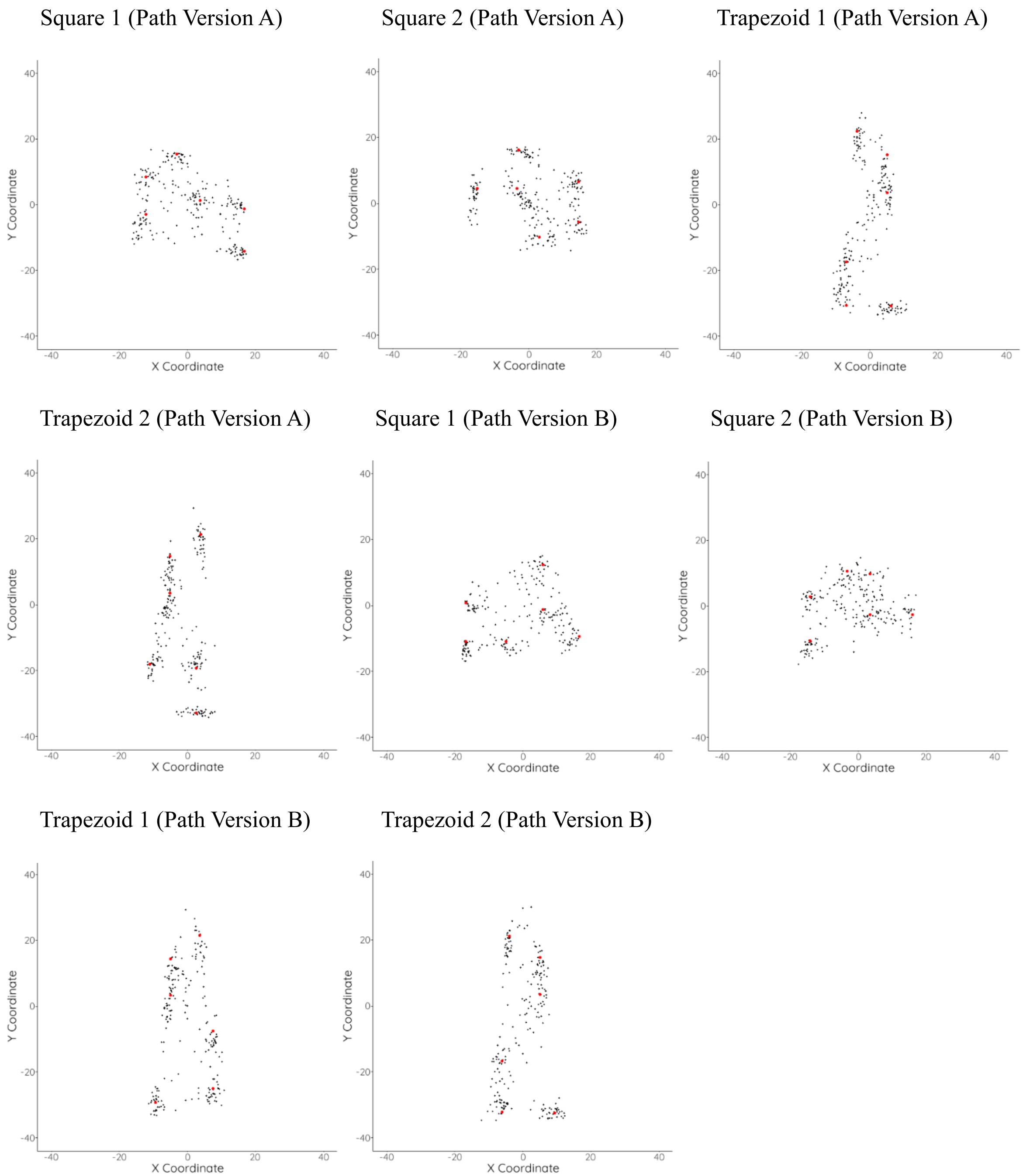

Supplementary Figure 21. **First-Person Test Spatial Distribution Plots – Non-Global.** Spatial distribution plots of participant responses are displayed for each environment above (n = 78 participants). Correct object locations are represented by red dots. Participant responses are represented by black dots. Within an environment, each participant's responses were averaged across trials to yield one dot per object (six total).

S22. Overhead Test Spatial Distribution Plots – Non-Global

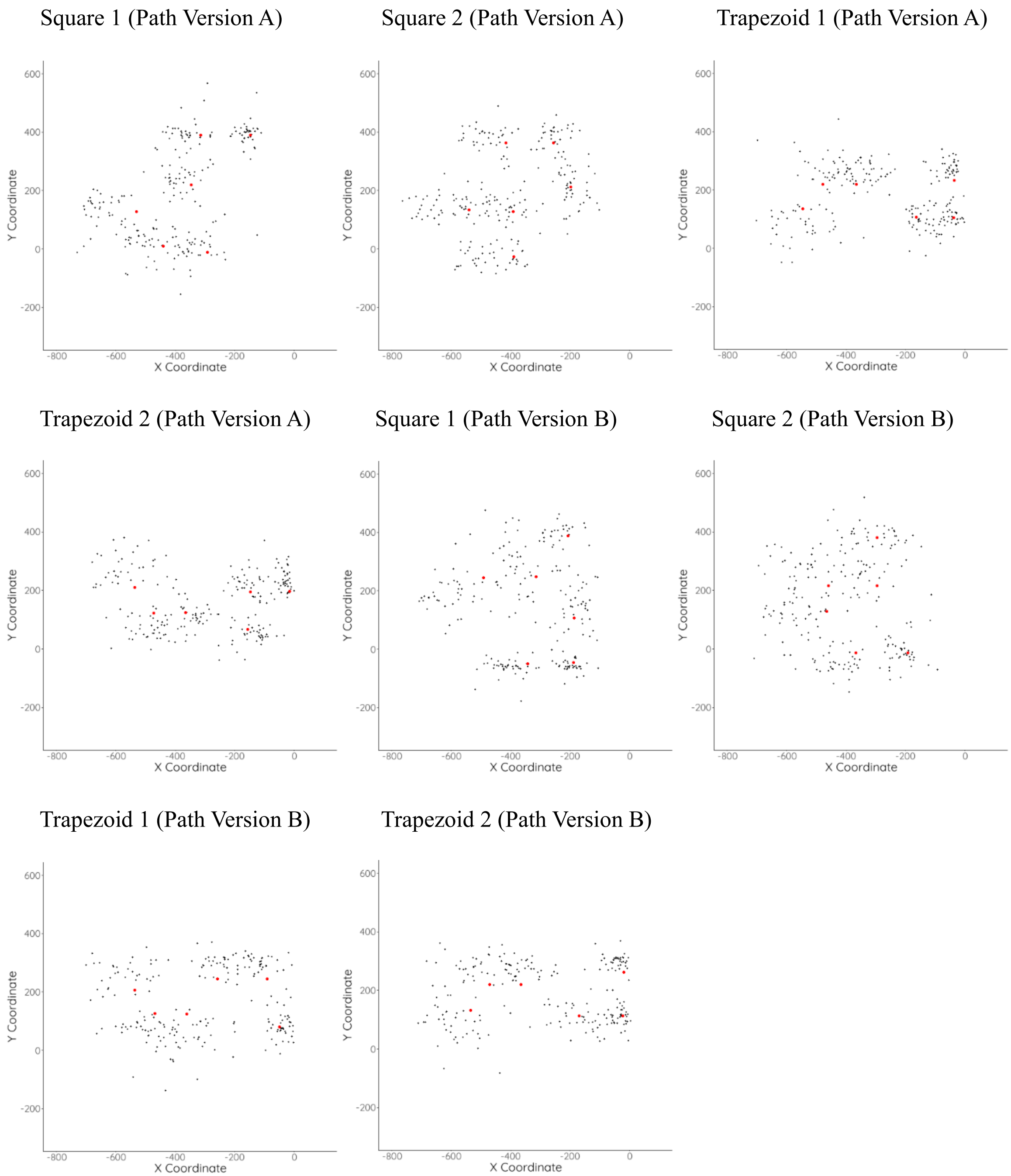

Supplementary Figure 22. **Overhead Test Spatial Distribution Plots – Non-Global.** Spatial distribution plots of participant responses are displayed for each environment above (n = 78 participants). Correct object locations are represented by red dots. Participant responses are represented by black dots (one point per each object in environment, six total).

SUPPLEMENTARY TABLES

Primary Dataset

Supplementary Table 1: Effect of Shape in First-Person Test

Effect	df	F	p
Environment Order	3, 796.53	10.883	< .001
Shape	1, 796.12	0.453	0.501
Trial	2, 795.36	105.041	< .001
Environment Order ✻ Shape	3, 864.75	0.314	0.815
Environment Order ✻ Trial	6, 795.36	0.807	0.565
Shape ✻ Trial	2, 795.36	1.560	0.211
Environment Order ✻ Shape ✻ Trial	6, 795.36	0.244	0.962

ANOVA summary for a mixed-effects model of Shape in the first-person test (outcome variable: mean spatial memory error within a trial; fixed effects: environment order, Shape, trial; random effect: subject ID).

Supplementary Table 2: Effect of Shape in Overhead Test

Effect	df	F	p
Environment Order	3, 210.00	1.790	0.150
Shape	1, 210.41	0.200	0.655
Environment Order ✻ Shape	3, 248.67	1.908	0.129

ANOVA summary for a mixed-effects model of Shape in the overhead test (outcome variable: mean spatial memory error within a trial; fixed effects: environment order, Shape; random effect: subject ID).

Supplementary Table 3: Effect of Object Serial Order in First-Person Test

Effect	df	F	p
Object Order	5, 5140.17	33.630	< .001
Environment Order	3, 5144.36	21.381	< .001
Shape	1, 5142.88	0.882	0.348
Trial	2, 5140.26	206.170	< .001
Object Order ✻ Environment Order	15, 5140.17	1.167	0.290
Object Order ✻ Shape	5, 5140.17	2.420	0.034
Environment Order ✻ Shape	3, 4823.91	0.818	0.483
Object Order ✻ Trial	10, 5140.17	2.831	0.002
Environment Order ✻ Trial	6, 5140.26	1.586	0.147
Shape ✻ Trial	2, 5140.26	3.049	0.047
Object Order ✻ Environment Order ✻ Shape	15, 5140.17	0.920	0.541
Object Order ✻ Environment Order ✻ Trial	30, 5140.17	0.962	0.525
Object Order ✻ Shape ✻ Trial	10, 5140.17	0.882	0.549
Environment Order ✻ Shape ✻ Trial	6, 5140.26	0.475	0.828
Object Order ✻ Environment Order ✻ Shape ✻ Trial	30, 5140.17	0.680	0.905

ANOVA summary for a mixed-effects model of object serial order in the first-person test (outcome variable: individual object spatial memory error; fixed effects: object order, environment order, Shape, trial; random effect: subject ID).

Supplementary Table 4: Effect of Object Serial Order in Overhead Test

Effect	df	F	p
Object Order	5, 1583.59	10.964	< .001
Environment Order	3, 1587.02	5.026	0.002
Shape	1, 1589.01	0.089	0.766
Object Order ✻ Environment Order	15, 1583.75	1.278	0.208
Object Order ✻ Shape	5, 1583.60	8.600	< .001
Environment Order ✻ Shape	3, 1653.33	3.731	0.011
Object Order ✻ Environment Order ✻ Shape	15, 1583.75	0.734	0.751

ANOVA summary for a mixed-effects model of object serial order in the overhead test (outcome variable: individual object spatial memory error; fixed effects: object order, environment order, Shape; random effect: subject ID).

Supplementary Table 5: Effect of Sense of Direction in First-Person Test

Effect	df	F	p
Environment Order	3, 777.69	1.772	0.151
Shape	1, 773.55	0.483	0.487
Trial	2, 772.92	12.712	< .001
SOD	1, 73.35	6.237	0.015
Environment Order ✻ Shape	3, 806.53	0.593	0.620
Environment Order ✻ Trial	6, 772.92	1.075	0.376
Shape ✻ Trial	2, 772.92	0.206	0.814
Environment Order ✻ SOD	3, 777.13	1.390	0.245
Shape ✻ SOD	1, 773.28	0.258	0.611
Trial ✻ SOD	2, 772.92	1.392	0.249
Environment Order ✻ Shape ✻ Trial	6, 772.92	0.179	0.983
Environment Order ✻ Shape ✻ SOD	3, 817.01	0.514	0.673
Environment Order ✻ Trial ✻ SOD	6, 772.92	0.742	0.616
Shape ✻ Trial ✻ SOD	2, 772.92	0.577	0.562
Environment Order ✻ Shape ✻ Trial ✻ SOD	6, 772.92	0.219	0.971

ANOVA summary for a mixed-effects model of sense of direction (SOD) in the first-person test (outcome variable: mean spatial memory error within a trial; fixed effects: environment order, Shape, trial, SOD; random effect: subject ID).

Supplementary Table 6: Effect of Sense of Direction in Overhead Test

Effect	df	F	p
Environment Order	3, 205.04	0.655	0.581
Shape	1, 204.14	5.775	0.017
SOD	1, 72.35	3.092	0.083
Environment Order ✻ Shape	3, 247.05	0.904	0.440
Environment Order ✻ SOD	3, 205.02	1.147	0.331
Shape ✻ SOD	1, 204.48	6.766	0.010
Environment Order ✻ Shape ✻ SOD	3, 247.19	0.883	0.451

ANOVA summary for a mixed-effects model of sense of direction (SOD) in the overhead test (outcome variable: mean spatial memory error within a trial; fixed effects: environment order, Shape, trial, SOD; random effect: subject ID).

Supplementary Table 7: Effect of Object Serial Order in First-Person Square Test

Effect	df	F	p
Object Order	5, 2536.03	12.175	< .001
Environment Order	3, 2509.97	9.129	< .001
Trial	2, 2536.03	152.229	< .001
Object Order ✻ Environment Order	15, 2536.03	1.368	0.154
Object Order ✻ Trial	10, 2536.03	2.554	0.005
Environment Order ✻ Trial	6, 2536.03	1.625	0.136
Object Order ✻ Environment Order ✻ Trial	30, 2536.03	0.797	0.775

ANOVA summary for a mixed-effects model of object serial order in the first-person test for square environments only (outcome variable: individual object spatial memory error; fixed effects: object order, environment order, trial; random effect: subject ID).

Supplementary Table 8: Effect of Object Serial Order in First-Person Trapezoid Test

Effect	df	F	p
Object Order	5, 2530.33	23.888	< .001
Environment Order	3, 2399.51	12.771	< .001
Trial	2, 2530.69	73.576	< .001
Object Order ✻ Environment Order	15, 2530.33	0.868	0.601
Object Order ✻ Trial	10, 2530.33	1.430	0.161
Environment Order ✻ Trial	6, 2530.69	0.676	0.669
Object Order ✻ Environment Order ✻ Trial	30, 2530.33	0.900	0.623

ANOVA summary for a mixed-effects model of object serial order in the first-person test for trapezoid environments only (outcome variable: individual object spatial memory error; fixed effects: object order, environment order, trial; random effect: subject ID).

Supplementary Table 9: Effect of Object Serial Order in Overhead Square Test

Effect	df	F	p
Object Order	5, 729.60	6.412	< .001
Environment Order	3, 794.61	0.270	0.847
Object Order ✻ Environment Order	15, 730.06	1.141	0.315

ANOVA summary for a mixed-effects model of object serial order in the overhead test for square environments only (outcome variable: individual object spatial memory error; fixed effects: object order, environment order; random effect: subject ID).

Supplementary Table 10: Effect of Object Serial Order in Overhead Trapezoid Test

Effect	df	F	p
Object Order	5, 778.14	15.443	< .001
Environment Order	3, 837.63	4.515	0.004
Object Order ✻ Environment Order	15, 778.14	1.250	0.228

ANOVA summary for a mixed-effects model of object serial order in the overhead test for trapezoid environments only (outcome variable: individual object spatial memory error; fixed effects: object order, environment order; random effect: subject ID).

Supplementary Table 11: Effect of Trapezoid Subregion in First-Person Test

Effect	df	F	p
Environment Order	3, 2428.15	12.575	< .001
Trial	2, 2578.70	72.488	< .001
Narrow vs. Wide	1, 2578.33	76.056	< .001
Environment Order ✻ Trial	6, 2578.71	0.666	0.677
Environment Order ✻ Narrow vs. Wide	3, 2578.33	0.269	0.848
Trial ✻ Narrow vs. Wide	2, 2578.33	1.296	0.274
Environment Order ✻ Trial ✻ Narrow vs. Wide	6, 2578.33	0.941	0.464

ANOVA summary for a mixed-effects model of trapezoid subregion in the first-person test (outcome variable: individual object spatial memory error; fixed effects: environment order, trial, narrow vs. wide; random effect: subject ID).

Supplementary Table 12: Effect of Trapezoid Subregion in Overhead Test

Effect	df	F	p
Environment Order	3, 853.83	4.574	0.003
Narrow vs. Wide	1, 794.15	87.255	< .001
Environment Order ✻ Narrow vs. Wide	3, 794.15	1.088	0.353

ANOVA summary for a mixed-effects model of trapezoid subregion in the overhead test (outcome variable: individual object spatial memory error; fixed effects: environment order, trial, narrow vs. wide; random effect: subject ID).

Supplementary Table 13: Effect of Wide Trapezoid Subregion vs. Square in First-Person Test

Effect	df	F	p
Environment Order	3, 2473.13	11.609	< .001
Trial	2, 2467.01	91.711	< .001
Wide vs. Square	1, 2471.01	3.885	0.049
Environment Order ✻ Trial	6, 2467.01	1.065	0.381
Environment Order ✻ Wide vs. Square	3, 2291.00	0.556	0.644
Trial ✻ Wide vs. Square	2, 2467.01	1.551	0.212
Environment Order ✻ Trial ✻ Wide vs. Square	6, 2467.01	0.435	0.856

ANOVA summary for a mixed-effects model of wide trapezoid subregion vs. square in the first-person test (outcome variable: individual object spatial memory error; fixed effects: environment order, trial, wide vs. square; random effect: subject ID). This model only includes data for objects in the wide end of the trapezoid and in the square that share the same object serial orders (1, 3, and 5).

Supplementary Table 14: Effect of Narrow Trapezoid Subregion vs. Square in First-Person Test

Effect	df	F	p
Environment Order	3, 2473.89	8.237	< .001
Trial	2, 2467.21	100.309	< .001
Narrow vs. Square	1, 2471.59	9.661	0.002
Environment Order ✻ Trial	6, 2467.21	0.899	0.495
Environment Order ✻ Narrow vs. Square	3, 2226.29	0.256	0.857
Trial ✻ Narrow vs. Square	2, 2467.21	1.731	0.177
Environment Order ✻ Trial ✻ Narrow vs. Square	6, 2467.21	0.437	0.854

ANOVA summary for a mixed-effects model of narrow trapezoid subregion vs. square in the first-person test (outcome variable: individual object spatial memory error; fixed effects: environment order, trial, narrow vs. square; random effect: subject ID). This model only includes data for objects in the narrow end of the trapezoid and in the square that share the same object serial orders (2, 4, and 6).

Supplementary Table 15: Effect of Wide Trapezoid Subregion vs. Square in Overhead Test

Effect	df	F	p
Environment Order	3, 756.70	1.879	0.132
Wide vs. Square	1, 757.26	10.201	0.001
Environment Order ✻ Wide vs. Square	3, 824.91	2.754	0.042

ANOVA summary for a mixed-effects model of wide trapezoid subregion vs. square in the overhead test (outcome variable: individual object spatial memory error; fixed effects: environment order, wide vs. square; random effect: subject ID). This model only includes data for objects in the wide end of the trapezoid and in the square that share the same object serial orders (1, 3, and 5).

Supplementary Table 16: Effect of Narrow Trapezoid Subregion vs. Square in Overhead Test

Effect	df	F	p
Environment Order	3, 713.75	3.276	0.021
Narrow vs. Square	1, 715.26	12.424	< .001
Environment Order ✻ Narrow vs. Square	3, 775.75	1.535	0.204

ANOVA summary for a mixed-effects model of narrow trapezoid subregion vs. square in the overhead test (outcome variable: individual object spatial memory error; fixed effects: environment order, narrow vs. square; random effect: subject ID). This model only includes data for objects in the narrow end of the trapezoid and in the square that share the same object serial orders (2, 4, and 6).

Supplementary Table 17: Effect of High Sense of Direction in Overhead Test

Effect	df	F	p
Environment Order	3, 99.83	1.581	0.199
Shape	1, 99.01	6.890	0.010
SOD	1, 38.70	1.486	0.230
Environment Order ✻ Shape	3, 115.46	1.066	0.367
Environment Order ✻ SOD	3, 99.99	1.893	0.136
Shape ✻ SOD	1, 99.17	7.958	0.006
Environment Order ✻ Shape ✻ SOD	3, 115.50	1.262	0.291

ANOVA summary for a mixed-effects model of subjects with sense of direction (SOD) scores greater than or equal to 4.6 in the overhead test (outcome variable: mean spatial memory error within a trial; fixed effects: environment order, Shape, SOD; random effect: subject ID).

Supplementary Table 18: Effect of High Sense of Direction in Overhead Square Test

Effect	df	F	p
Environment Order	3, 35.90	1.205	0.322
SOD	1, 37.17	7.218	0.011
Environment Order ✻ SOD	3, 35.98	1.087	0.367

ANOVA summary for a mixed-effects model of subjects with sense of direction (SOD) scores greater than or equal to 4.6 in the overhead test (outcome variable: mean spatial memory error within a trial; fixed effects: environment order, SOD; random effect: subject ID). This model only includes data from square environments.

Supplementary Table 19: Effect of High Sense of Direction in Overhead Trapezoid Test

Effect	df	F	p
Environment Order	3, 37.79	1.244	0.307
SOD	1, 38.05	0.004	0.952
Environment Order ✻ SOD	3, 37.67	1.464	0.240

ANOVA summary for a mixed-effects model of subjects with sense of direction (SOD) scores greater than or equal to 4.6 in the overhead test (outcome variable: mean spatial memory error within a trial; fixed effects: environment order, SOD; random effect: subject ID). This model only includes data from trapezoid environments.

Supplementary Table 20: Effect of Low Sense of Direction Overhead Test

Effect	df	F	p
Environment Order	3, 91.82	0.990	0.401
SOD	1, 32.62	0.027	0.870
Shape	1, 91.36	0.247	0.621
Environment Order ✻ SOD	3, 91.86	1.095	0.355
Environment Order ✻ Shape	3, 114.36	1.202	0.312
SOD ✻ Shape	1, 91.37	0.034	0.854
Environment Order ✻ SOD ✻ Shape	3, 115.21	1.304	0.277

ANOVA summary for a mixed-effects model of subjects with sense of direction (SOD) scores less than 4.6 in the overhead test (outcome variable: mean spatial memory error within a trial; fixed effects: environment order, Shape, SOD; random effect: subject ID).

Supplementary Table 21: Effect of Low Sense of Direction Overhead Square Test

Effect	df	F	p
Environment Order	3, 39.08	2.875	0.048
SOD	1, 27.72	0.011	0.917
Environment Order ✻ SOD	3, 39.85	3.319	0.029

ANOVA summary for a mixed-effects model of subjects with sense of direction (SOD) scores less than 4.6 in the overhead test (outcome variable: mean spatial memory error within a trial; fixed effects: environment order, SOD; random effect: subject ID). This model only includes data from square environments.

Supplementary Table 22: Effect of Low Sense of Direction Overhead Trapezoid Test

Effect	df	F	p
Environment Order	3, 40.16	0.667	0.577
SOD	1, 33.30	0.019	0.892
Environment Order ✻ SOD	3, 40.40	0.382	0.766

ANOVA summary for a mixed-effects model of subjects with sense of direction (SOD) scores less than 4.6 in the overhead test (outcome variable: mean spatial memory error within a trial; fixed effects: environment order, SOD; random effect: subject ID). This model only includes data from trapezoid environments.

Procrustes Analysis

Supplementary Table 23: Effect of Shape in First-Person Test

Effect	df	F	p
Environment Order	3, 796.52	8.758	< .001
Shape	1, 796.12	3.752	0.053
Trial	2, 795.40	124.965	< .001
Environment Order ✻ Shape	3, 864.99	1.455	0.226
Environment Order ✻ Trial	6, 795.40	0.945	0.462
Shape ✻ Trial	2, 795.40	0.579	0.561
Environment Order ✻ Shape ✻ Trial	6, 795.40	0.194	0.978

ANOVA summary for a mixed-effects model of Shape in the first-person test (outcome variable: mean spatial memory error within a trial; fixed effects: environment order, Shape, trial; random effect: subject ID).

Supplementary Table 24: Effect of Shape in Overhead Test

Effect	df	F	p
Environment Order	3, 212.68	2.271	0.081
Shape	1, 213.29	3.855	0.051
Environment Order ✻ Shape	3, 273.96	2.876	0.037

ANOVA summary for a mixed-effects model of Shape in the overhead test (outcome variable: mean spatial memory error within a trial; fixed effects: environment order, Shape; random effect: subject ID).

Supplementary Table 25: Effect of Object Serial Order in First-Person Test

Effect	df	F	p
Environment Order	3, 5143.13	23.751	< .001
Trial	2, 5140.21	339.126	< .001
Object Order	5, 5140.14	15.065	< .001
Shape	1, 5142.05	10.109	0.001
Environment Order ✻ Trial	6, 5140.21	2.562	0.018
Environment Order ✻ Object Order	15, 5140.14	0.898	0.566
Trial ✻ Object Order	10, 5140.14	1.985	0.031
Environment Order ✻ Shape	3, 5074.63	4.517	0.004
Trial ✻ Shape	2, 5140.21	1.566	0.209
Object Order ✻ Shape	5, 5140.14	3.265	0.006
Environment Order ✻ Trial ✻ Object Order	30, 5140.14	0.696	0.891
Environment Order ✻ Trial ✻ Shape	6, 5140.21	0.529	0.787
Environment Order ✻ Object Order ✻ Shape	15, 5140.14	0.881	0.586
Trial ✻ Object Order ✻ Shape	10, 5140.14	1.403	0.172
Environment Order ✻ Trial ✻ Object Order ✻ Shape	30, 5140.14	0.756	0.828

ANOVA summary for a mixed-effects model of object serial order in the first-person test (outcome variable: individual object spatial memory error; fixed effects: object order, environment order, Shape, trial; random effect: subject ID).

Supplementary Table 26: Effect of Object Serial Order in Overhead Test

Effect	df	F	p
Object Order	5, 1584.54	5.550	< .001
Environment Order	3, 1590.55	7.438	< .001
Shape	1, 1593.93	9.564	0.002
Object Order * Environment Order	15, 1584.85	0.762	0.721
Object Order * Shape	5, 1584.56	5.899	< .001
Environment Order * Shape	3, 1614.45	7.857	< .001
Object Order * Environment Order * Shape	15, 1584.84	0.507	0.938

ANOVA summary for a mixed-effects model of object serial order in the overhead test (outcome variable: individual object spatial memory error; fixed effects: object order, environment order, Shape; random effect: subject ID).

Supplementary Table 27: Effect of Sense of Direction in First-Person Test

Effect	df	F	p
Environment Order	3, 777.44	1.616	0.184
Shape	1, 773.48	2.057	0.152
Trial	2, 772.90	16.781	< .001
SOD	1, 73.38	5.779	0.019
Environment Order * Shape	3, 812.56	2.525	0.056
Environment Order * Trial	6, 772.90	1.288	0.260
Shape * Trial	2, 772.90	0.801	0.449
Environment Order * SOD	3, 776.91	0.947	0.418
Shape * SOD	1, 773.23	0.932	0.335
Trial * SOD	2, 772.89	2.350	0.096
Environment Order * Shape * Trial	6, 772.90	0.398	0.881
Environment Order * Shape * SOD	3, 821.97	1.850	0.137
Environment Order * Trial * SOD	6, 772.89	0.904	0.491
Shape * Trial * SOD	2, 772.89	1.161	0.314
Environment Order * Shape * Trial * SOD	6, 772.89	0.316	0.929

ANOVA summary for a mixed-effects model of sense of direction (SOD) in the first-person test (outcome variable: mean spatial memory error within a trial; fixed effects: environment order, Shape, trial, SOD; random effect: subject ID).

Supplementary Table 28: Effect of Sense of Direction in Overhead Test

Effect	df	F	p
Environment Order	3, 208.60	0.201	0.896
Shape	1, 207.39	2.559	0.111
SOD	1, 73.94	4.721	0.033
Environment Order * Shape	3, 262.90	1.090	0.354
Environment Order * SOD	3, 208.58	0.594	0.620
Shape * SOD	1, 207.87	4.621	0.033
Environment Order * Shape * SOD	3, 265.24	0.704	0.550

ANOVA summary for a mixed-effects model of sense of direction (SOD) in the overhead test (outcome variable: mean spatial memory error within a trial; fixed effects: environment order, Shape, trial, SOD; random effect: subject ID).

Supplementary Table 29: Effect of Trapezoid Subregion in First-Person Test

Effect	df	F	p
Environment Order	3, 2602.02	22.607	< .001
Trial	2, 2578.40	127.957	< .001
Narrow vs. Wide	1, 2578.17	28.149	< .001
Environment Order ✻ Trial	6, 2578.41	1.528	0.165
Environment Order ✻ Narrow vs. Wide	3, 2578.17	0.729	0.535
Trial ✻ Narrow vs. Wide	2, 2578.17	0.943	0.390
Environment Order ✻ Trial ✻ Narrow vs. Wide	6, 2578.17	0.529	0.787

ANOVA summary for a mixed-effects model of trapezoid subregion in the first-person test (outcome variable: individual object spatial memory error; fixed effects: environment order, trial, narrow vs. wide; random effect: subject ID).

Supplementary Table 30: Effect of Trapezoid Subregion in Overhead Test

Effect	df	F	p
Environment Order	3, 857.66	13.637	< .001
Narrow vs. Wide	1, 794.49	29.388	< .001
Environment Order ✻ Narrow vs. Wide	3, 794.49	0.907	0.437

ANOVA summary for a mixed-effects model of trapezoid subregion in the overhead test (outcome variable: individual object spatial memory error; fixed effects: environment order, trial, narrow vs. wide; random effect: subject ID).

Supplementary Table 31: Effect of Wide Trapezoid Subregion vs. Square in First-Person Test

Effect	df	F	p
Environment Order	3, 2471.41	12.135	< .001
Trial	2, 2467.03	171.975	< .001
Wide vs. Square	1, 2469.86	0.223	0.637
Environment Order ✻ Trial	6, 2467.03	1.829	0.090
Environment Order ✻ Wide vs. Square	3, 2451.33	4.502	0.004
Trial ✻ Wide vs. Square	2, 2467.03	2.189	0.112
Environment Order ✻ Trial ✻ Wide vs. Square	6, 2467.03	1.108	0.355

ANOVA summary for a mixed-effects model of wide trapezoid subregion vs. square in the first-person test (outcome variable: individual object spatial memory error; fixed effects: environment order, trial, wide vs. square; random effect: subject ID). This model only includes data for objects in the wide end of the trapezoid and in the square that share the same object serial orders (1, 3, and 5).

Supplementary Table 32: Effect of Narrow Trapezoid Subregion vs. Square in First-Person Test

Effect	df	F	p
Environment Order	3, 2471.92	10.630	< .001
Trial	2, 2467.07	152.080	< .001
Narrow vs. Square	1, 2470.22	18.023	< .001
Environment Order ✻ Trial	6, 2467.07	1.545	0.160
Environment Order ✻ Narrow vs. Square	3, 2413.96	0.836	0.474
Trial ✻ Narrow vs. Square	2, 2467.07	0.404	0.668
Environment Order ✻ Trial ✻ Narrow vs. Square	6, 2467.07	0.693	0.656

ANOVA summary for a mixed-effects model of narrow trapezoid subregion vs. square in the first-person test (outcome variable: individual object spatial memory error; fixed effects: environment order, trial, narrow vs. square; random effect: subject ID). This model only includes data for objects in the narrow end of the trapezoid and in the square that share the same object serial orders (2, 4, and 6).

Supplementary Table 33: Effect of Wide Trapezoid Subregion vs. Square in Overhead Test

Effect	df	F	p
Environment Order	3, 761.19	2.148	0.093
Wide vs. Square	1, 761.79	0.022	0.882
Environment Order ✻ Wide vs. Square	3, 819.41	4.148	0.006

ANOVA summary for a mixed-effects model of wide trapezoid subregion vs. square in the overhead test (outcome variable: individual object spatial memory error; fixed effects: environment order, wide vs. square; random effect: subject ID). This model only includes data for objects in the wide end of the trapezoid and in the square that share the same object serial orders (1, 3, and 5).

Supplementary Table 34: Effect of Narrow Trapezoid Subregion vs. Square in Overhead Test

Effect	df	F	p
Environment Order	3, 719.20	5.310	0.001
Narrow vs. Square	1, 722.12	27.947	< .001
Environment Order ✻ Narrow vs. Square	3, 748.96	4.328	0.005

ANOVA summary for a mixed-effects model of narrow trapezoid subregion vs. square in the overhead test (outcome variable: individual object spatial memory error; fixed effects: environment order, narrow vs. square; random effect: subject ID). This model only includes data for objects in the narrow end of the trapezoid and in the square that share the same object serial orders (2, 4, and 6).

Supplementary Table 35: Effect of High Sense of Direction in Overhead Test

Effect	df	F	p
Environment Order	3, 104.71	0.401	0.753
Shape	1, 102.76	2.855	0.094
SOD	1, 42.59	1.012	0.320
Environment Order ✻ Shape	3, 132.71	0.144	0.933
Environment Order ✻ SOD	3, 105.01	0.431	0.731
Shape ✻ SOD	1, 103.08	3.766	0.055
Environment Order ✻ Shape ✻ SOD	3, 132.68	0.182	0.909

ANOVA summary for a mixed-effects model of subjects with sense of direction (SOD) scores greater than or equal to 4.6 in the overhead test (outcome variable: mean spatial memory error within a trial; fixed effects: environment order, Shape, SOD; random effect: subject ID).

Supplementary Table 36: Effect of Low Sense of Direction in Overhead Test

Effect	df	F	p
Environment Order	3, 94.41	0.997	0.398
Shape	1, 93.82	0.260	0.611
SOD	1, 33.72	1.452	0.237
Environment Order ✻ Shape	3, 117.74	0.442	0.724
Environment Order ✻ SOD	3, 94.47	1.289	0.283
Shape ✻ SOD	1, 93.83	0.304	0.583
Environment Order ✻ Shape ✻ SOD	3, 119.11	0.181	0.909

ANOVA summary for a mixed-effects model of subjects with sense of direction (SOD) scores less than 4.6 in the overhead test (outcome variable: mean spatial memory error within a trial; fixed effects: environment order, Shape, SOD; random effect: subject ID).

Procrustes without scaling

Supplementary Table 37: Effect of Wide Trapezoid Subregion vs. Square in Overhead Test

Effect	df	F	p
Environment Order	3, 761.60	4.012	0.008
Wide vs. Square	1, 762.22	0.145	0.703
Environment Order ✻ Wide vs. Square	3, 812.47	2.534	0.056

ANOVA summary for a mixed-effects model of wide trapezoid subregion vs. square in the overhead test (outcome variable: individual object spatial memory error; fixed effects: environment order, wide vs. square; random effect: subject ID). This model only includes data for objects in the wide end of the trapezoid and in the square that share the same object serial orders (1, 3, and 5).

Supplementary Table 38: Effect of Wide Trapezoid Subregion vs. Square in First-Person Test

Effect	df	F	p
Trial	2, 2466.93	122.063	< .001
Environment Order	3, 2472.47	6.915	< .001
Wide vs. Square	1, 2470.54	2.516	0.113
Trial ✻ Environment Order	6, 2466.93	1.098	0.361
Trial ✻ Wide vs. Square	2, 2466.93	1.472	0.230
Environment Order ✻ Wide vs. Square	3, 2351.64	1.488	0.216
Trial ✻ Environment Order ✻ Wide vs. Square	6, 2466.93	0.884	0.506

ANOVA summary for a mixed-effects model of wide trapezoid subregion vs. square in the first-person test (outcome variable: individual object spatial memory error; fixed effects: environment order, trial, wide vs. square; random effect: subject ID). This model only includes data for objects in the wide end of the trapezoid and in the square that share the same object serial orders (1, 3, and 5).

Non-Global Analysis

Supplementary Table 39: Effect of Shape in First-Person Test

Effect	df	F	p
Environment Order	3, 824.82	5.363	0.001
Shape	1, 824.52	0.248	0.618
Trial	2, 823.09	145.413	< .001
Environment Order ✻ Shape	3, 893.61	0.160	0.924
Environment Order ✻ Trial	6, 823.09	0.543	0.775
Shape ✻ Trial	2, 823.09	0.868	0.420
Environment Order ✻ Shape ✻ Trial	6, 823.09	0.366	0.900

ANOVA summary for a mixed-effects model of Shape in the first-person test (outcome variable: mean spatial memory error within a trial; fixed effects: environment order, Shape, trial; random effect: subject ID).

Supplementary Table 40: Effect of Shape in Overhead Test

Effect	df	F	p
Environment Order	3, 224.28	0.351	0.789
Shape	1, 224.13	11.256	< .001
Environment Order ✻ Shape	3, 269.51	0.499	0.683

ANOVA summary for a mixed-effects model of Shape in the overhead test (outcome variable: mean spatial memory error within a trial; fixed effects: environment order, Shape; random effect: subject ID).

Supplementary Table 41: Effect of Object Serial Order in First-Person Test

Effect	df	F	p
Environment Order	3, 5330.47	9.259	< .001
Trial	2, 5323.08	252.061	< .001
Shape	1, 5329.56	0.425	0.515
Object Order	5, 5323.08	30.851	< .001
Environment Order ✻ Trial	6, 5323.08	0.942	0.464
Environment Order ✻ Shape	3, 5180.29	0.332	0.802
Trial ✻ Shape	2, 5323.08	1.505	0.222
Environment Order ✻ Object Order	15, 5323.08	1.271	0.211
Trial ✻ Object Order	10, 5323.08	4.964	< .001
Shape ✻ Object Order	5, 5323.08	4.640	< .001
Environment Order ✻ Trial ✻ Shape	6, 5323.08	0.635	0.702
Environment Order ✻ Trial ✻ Object Order	30, 5323.08	0.833	0.726
Environment Order ✻ Shape ✻ Object Order	15, 5323.08	0.581	0.892
Trial ✻ Shape ✻ Object Order	10, 5323.08	1.385	0.180
Environment Order ✻ Trial ✻ Shape ✻ Object Order	30, 5323.08	0.541	0.980

ANOVA summary for a mixed-effects model of object serial order in the first-person test (outcome variable: individual object spatial memory error; fixed effects: object order, environment order, Shape, trial; random effect: subject ID).

Supplementary Table 42: Effect of Object Serial Order in Overhead Test

Effect	df	F	p
Environment Order	3, 1722.99	0.830	0.477
Shape	1, 1722.57	28.642	< .001
Object Order	5, 1720.13	16.740	< .001
Environment Order ✻ Shape	3, 1794.20	1.095	0.350
Environment Order ✻ Object Order	15, 1720.13	0.590	0.884
Shape ✻ Object Order	5, 1720.13	13.232	< .001
Environment Order ✻ Shape ✻ Object Order	15, 1720.13	0.669	0.817

ANOVA summary for a mixed-effects model of object serial order in the overhead test (outcome variable: individual object spatial memory error; fixed effects: object order, environment order, Shape; random effect: subject ID).

Supplementary Table 43: Effect of Sense of Direction in First-Person Test

Effect	df	F	p
Environment Order	3, 803.41	1.919	0.125
Shape	1, 799.91	1.740	0.188
Trial	2, 799.71	22.240	< .001
SOD	1, 75.05	15.210	< .001
Environment Order ✻ Shape	3, 871.94	1.527	0.206
Environment Order ✻ Trial	6, 799.71	0.621	0.714
Shape ✻ Trial	2, 799.71	0.305	0.737
Environment Order ✻ SOD	3, 803.82	1.134	0.335
Shape ✻ SOD	1, 800.17	2.205	0.138
Trial ✻ SOD	2, 799.71	3.798	0.023
Environment Order ✻ Shape ✻ Trial	6, 799.71	0.587	0.741
Environment Order ✻ Shape ✻ SOD	3, 872.22	1.839	0.139
Environment Order ✻ Trial ✻ SOD	6, 799.71	0.574	0.751
Shape ✻ Trial ✻ SOD	2, 799.71	0.119	0.888
Environment Order ✻ Shape ✻ Trial ✻ SOD	6, 799.71	0.450	0.845

ANOVA summary for a mixed-effects model of sense of direction (SOD) in the first-person test (outcome variable: mean spatial memory error within a trial; fixed effects: environment order, Shape, trial, SOD; random effect: subject ID).

Supplementary Table 44: Effect of Sense of Direction in Overhead Test

Effect	df	F	p
Environment Order	3, 218.59	1.192	0.314
Shape	1, 217.20	0.127	0.722
SOD	1, 76.14	4.078	0.047
Environment Order ✻ Shape	3, 263.78	0.235	0.872
Environment Order ✻ SOD	3, 218.73	1.052	0.370
Shape ✻ SOD	1, 217.28	0.204	0.652
Environment Order ✻ Shape ✻ SOD	3, 263.97	0.193	0.901

ANOVA summary for a mixed-effects model of sense of direction (SOD) in the overhead test (outcome variable: mean spatial memory error within a trial; fixed effects: environment order, Shape, trial, SOD; random effect: subject ID).

Supplementary Table 45: Effect of Trapezoid Subregion in First-Person Test

Effect	df	F	p
Environment Order	3, 2433.43	4.082	0.007
Trial	2, 2653.67	109.761	< .001
Narrow vs. Wide	1, 2653.67	46.648	< .001
Environment Order ✻ Trial	6, 2653.67	0.591	0.738
Environment Order ✻ Narrow vs. Wide	3, 2653.67	1.870	0.132
Trial ✻ Narrow vs. Wide	2, 2653.67	2.232	0.107
Environment Order ✻ Trial ✻ Narrow vs. Wide	6, 2653.67	0.794	0.575

ANOVA summary for a mixed-effects model of trapezoid subregion in the first-person test (outcome variable: individual object spatial memory error; fixed effects: environment order, trial, narrow vs. wide; random effect: subject ID).

Supplementary Table 46: Effect of Trapezoid Subregion in Overhead Test

Effect	df	F	p
Narrow vs. Wide	1, 832.28	145.173	< .001
Environment Order	3, 886.22	0.744	0.526
Narrow vs. Wide ✻ Environment Order	3, 832.27	0.006	0.999

ANOVA summary for a mixed-effects model of trapezoid subregion in the overhead test (outcome variable: individual object spatial memory error; fixed effects: environment order, trial, narrow vs. wide; random effect: subject ID).

Supplementary Table 47: Effect of Wide Trapezoid Subregion vs. Square in First-Person Test

Effect	df	F	p
Environment Order	3, 2562.61	5.608	< .001
Trial	2, 2554.39	112.944	< .001
Wide vs. Square	1, 2560.09	2.398	0.122
Environment Order ✻ Trial	6, 2554.39	0.991	0.430
Environment Order ✻ Wide vs. Square	3, 2453.68	0.300	0.826
Trial ✻ Wide vs. Square	2, 2554.39	0.096	0.909
Environment Order ✻ Trial ✻ Wide vs. Square	6, 2554.39	0.820	0.554

ANOVA summary for a mixed-effects model of wide trapezoid subregion vs. square in the first-person test (outcome variable: individual object spatial memory error; fixed effects: environment order, trial, wide vs. square; random effect: subject ID). This model only includes data for objects in the wide end of the trapezoid and in the square that share the same object serial orders (1, 3, and 5).

Supplementary Table 48: Effect of Narrow Trapezoid Subregion vs. Square in First-Person Test

Effect	df	F	p
Environment Order	3, 2562.46	3.204	0.022
Trial	2, 2554.16	125.535	< .001
Narrow vs. Square	1, 2559.91	5.059	0.025
Environment Order ✻ Trial	6, 2554.16	0.523	0.791
Environment Order ✻ Narrow vs. Square	3, 2448.62	0.090	0.965
Trial ✻ Narrow vs. Square	2, 2554.16	2.411	0.090
Environment Order ✻ Trial ✻ Narrow vs. Square	6, 2554.16	0.755	0.605

ANOVA summary for a mixed-effects model of narrow trapezoid subregion vs. square in the first-person test (outcome variable: individual object spatial memory error; fixed effects: environment order, trial, narrow vs. square; random effect: subject ID). This model only includes data for objects in the narrow end of the trapezoid and in the square that share the same object serial orders (2, 4, and 6).

Supplementary Table 49: Effect of Wide Trapezoid Subregion vs. Square in Overhead Test

Effect	df	F	p
Wide vs. Square	1, 803.03	45.115	< .001
Environment Order	3, 804.10	0.155	0.927
Wide vs. Square ✻ Environment Order	3, 871.69	0.605	0.612

ANOVA summary for a mixed-effects model of wide trapezoid subregion vs. square in the overhead test (outcome variable: individual object spatial memory error; fixed effects: environment order, wide vs. square; random effect: subject ID). This model only includes data for objects in the wide end of the trapezoid and in the square that share the same object serial orders (1, 3, and 5).

Supplementary Table 50: Effect of Narrow Trapezoid Subregion vs. Square in Overhead Test

Effect	df	F	p
Narrow vs. Square	1, 800.23	0.084	0.771
Environment Order	3, 801.11	0.940	0.421
Narrow vs. Square ✻ Environment Order	3, 872.05	0.689	0.559

ANOVA summary for a mixed-effects model of narrow trapezoid subregion vs. square in the overhead test (outcome variable: individual object spatial memory error; fixed effects: environment order, narrow vs. square; random effect: subject ID). This model only includes data for objects in the narrow end of the trapezoid and in the square that share the same object serial orders (2, 4, and 6).

Supplementary Table 51: Effect of Object Serial Order in First-Person Square Test

Effect	df	F	p
Environment Order	3, 2696.85	2.468	0.060
Trial	2, 2641.01	154.904	< .001
Object Order	5, 2641.01	9.644	< .001
Environment Order ✻ Trial	6, 2641.01	1.097	0.362
Environment Order ✻ Object Order	15, 2641.01	1.253	0.224
Trial ✻ Object Order	10, 2641.01	1.452	0.151
Environment Order ✻ Trial ✻ Object Order	30, 2641.01	0.604	0.956

ANOVA summary for a mixed-effects model of object serial order in the first-person test for square environments only (outcome variable: individual object spatial memory error; fixed effects: object order, environment order, trial; random effect: subject ID).

Supplementary Table 52: Effect of Object Serial Order in First-Person Trapezoid Test

Effect	df	F	p
Environment Order	3, 2422.82	4.227	0.005
Trial	2, 2605.68	113.665	< .001
Object Order	5, 2605.68	24.775	< .001
Environment Order ✻ Trial	6, 2605.68	0.612	0.721
Environment Order ✻ Object Order	15, 2605.68	0.767	0.716
Trial ✻ Object Order	10, 2605.68	4.633	< .001
Environment Order ✻ Trial ✻ Object Order	30, 2605.68	0.800	0.771

ANOVA summary for a mixed-effects model of object serial order in the first-person test for trapezoid environments only (outcome variable: individual object spatial memory error; fixed effects: object order, environment order, trial; random effect: subject ID).

Supplementary Table 53: Effect of Object Serial Order in Overhead Square Test

Effect	df	F	p
Object Order	5, 827.44	11.806	< .001
Environment Order	3, 902.21	1.059	0.366
Object Order ✻ Environment Order	15, 827.44	0.550	0.912

ANOVA summary for a mixed-effects model of object serial order in the overhead test for square environments only (outcome variable: individual object spatial memory error; fixed effects: object order, environment order; random effect: subject ID).

Supplementary Table 54: Effect of Object Serial Order in Overhead Trapezoid Test

Effect	df	F	p
Object Order	5, 816.28	27.562	< .001
Environment Order	3, 869.85	0.749	0.523
Object Order ✻ Environment Order	15, 816.28	1.023	0.429

ANOVA summary for a mixed-effects model of object serial order in the overhead test for trapezoid environments only (outcome variable: individual object spatial memory error; fixed effects: object order, environment order; random effect: subject ID).
