# Supplementary material for: Environment geometry alters sequential route learning and its integration into cognitive maps
Source: Nat Commun. 2026 Jul 7;17:8400. doi: 10.1038/s41467-026-75129-y (PMC13473551; doi:10.1038/s41467-026-75129-y)
Supplement: Supplementary file 2 [file 41467_2026_75129_MOESM2_ESM.pdf]

nature portfolio

Corresponding author(s): Jaida Long

Last updated by author(s): Jan 28, 2026

# Reporting Summary

Nature Portfolio wishes to improve the reproducibility of the work that we publish. This form provides structure for consistency and transparency in reporting. For further information on Nature Portfolio policies, see our Editorial Policies and the Editorial Policy Checklist.

## Statistics

For all statistical analyses, confirm that the following items are present in the figure legend, table legend, main text, or Methods section.

| n/a | Confirmed | |
|---|---|---|
| ☐ | ☒ | The exact sample size (*n*) for each experimental group/condition, given as a discrete number and unit of measurement |
| ☐ | ☒ | A statement on whether measurements were taken from distinct samples or whether the same sample was measured repeatedly |
| ☐ | ☒ | The statistical test(s) used AND whether they are one- or two-sided<br>*Only common tests should be described solely by name; describe more complex techniques in the Methods section.* |
| ☐ | ☒ | A description of all covariates tested |
| ☐ | ☒ | A description of any assumptions or corrections, such as tests of normality and adjustment for multiple comparisons |
| ☐ | ☒ | A full description of the statistical parameters including central tendency (e.g. means) or other basic estimates (e.g. regression coefficient) AND variation (e.g. standard deviation) or associated estimates of uncertainty (e.g. confidence intervals) |
| ☐ | ☒ | For null hypothesis testing, the test statistic (e.g. *F*, *t*, *r*) with confidence intervals, effect sizes, degrees of freedom and *P* value noted<br>*Give P values as exact values whenever suitable.* |
| ☒ | ☐ | For Bayesian analysis, information on the choice of priors and Markov chain Monte Carlo settings |
| ☒ | ☐ | For hierarchical and complex designs, identification of the appropriate level for tests and full reporting of outcomes |
| ☐ | ☒ | Estimates of effect sizes (e.g. Cohen's *d*, Pearson's *r*), indicating how they were calculated |

*Our web collection on statistics for biologists contains articles on many of the points above.*

## Software and code

Policy information about availability of computer code

| | |
|---|---|
| Data collection | github.com/MAPLabgroup/CURCI-G |
| Data analysis | performed in Jasp 0.18.3.0 (see github.com/MAPLabgroup/CURCI-G) |

For manuscripts utilizing custom algorithms or software that are central to the research but not yet described in published literature, software must be made available to editors and reviewers. We strongly encourage code deposition in a community repository (e.g. GitHub). See the Nature Portfolio guidelines for submitting code & software for further information.

## Data

Policy information about availability of data

All manuscripts must include a data availability statement. This statement should provide the following information, where applicable:

- Accession codes, unique identifiers, or web links for publicly available datasets
- A description of any restrictions on data availability
- For clinical datasets or third party data, please ensure that the statement adheres to our policy

All display items presented in the main manuscript and supplementary information can be reproduced from material shared on GitHub (https://doi.org/10.5281/zenodo.1974026763): github.com/MAPLabgroup/CURCI-G. The material in the repository includes all data, including processed and raw data, in addition to any programming scripts used.

## Research involving human participants, their data, or biological material

Policy information about studies with human participants or human data. See also policy information about sex, gender (identity/presentation), and sexual orientation and race, ethnicity and racism.

| | |
|---|---|
| Reporting on sex and gender | Gender information was collected by self-report and the number of female participants (90) is indicated in the main text (female here indicating self-report gender, not biological sex). Disaggregated sex and gender data is not provided in the publicly available data - however it can be done so at request. Consent for sharing of individual-level data was given by some participants which can be provided upon request. No gender based analyses were performed. It is not reasonably expected that our effects of interest are influenced by gender. |
| Reporting on race, ethnicity, or other socially relevant groupings | These variables were not used in the manuscript. Self-report race and ethnicity (American Indian / Alaskan, Asian, Pacific Islander, Black / African American, White, Hispanic / Latino, and More than one race - circle all that apply) data was collected and recorded solely for the purpose of reporting to funding agencies. It was not necessary to control for these attributes as confounding variables in my analysis. |
| Population characteristics | We collected our data from healthy young adults. The mean age and standard deviation for our sample is reported in the main text. However, these were not covariate-relevant population characteristics. |
| Recruitment | Participants were recruited by advertising through the Georgia Tech SONA system and through word of mouth. This is unlikely to impact or bias results. Participants earned course credit or monetary compensation ($15/hr) for their participation, and they completed the task in around 1.5 hours. |
| Ethics oversight | The study protocol (#H23024), ethics, and consent form was approved by the Georgia Institute of Technology Institutional Review Board (IRB). Participants provided informed consent and signed the approved consent form before completing the task. |

Note that full information on the approval of the study protocol must also be provided in the manuscript.

# Field-specific reporting

Please select the one below that is the best fit for your research. If you are not sure, read the appropriate sections before making your selection.

☐ Life sciences ☒ Behavioural & social sciences ☐ Ecological, evolutionary & environmental sciences

For a reference copy of the document with all sections, see nature.com/documents/nr-reporting-summary-flat.pdf

# Behavioural & social sciences study design

All studies must disclose on these points even when the disclosure is negative.

| | |
|---|---|
| Study description | Data are quantitative. |
| Research sample | The research sample consisted of healthy young adults (Georgia Tech undergraduates and others from the surrounding Atlanta area). 77 adults were recruited (38 female) with mean age of 20.540 +- 2.9756 for the main analysis. For the supplemental analysis that we added for the revision, the sample was as follows: healthy young adults (N=82, 52 female, age = 20.049 ± 2.233) from the GT participant pool and surrounding Atlanta area. Our sample is representative of the populations we collected from. We chose our sample because healthy young adults are a population conducive to investigating our effects of interest. |
| Sampling strategy | The sample procedure was convenience. Previous power analyses demonstrate that environment geometry has a modest effect (d = 4) on spatial memory and necessitates a sample size of at least 52 for a statistical power of 80% (alpha = .05, two-tailed test) |
| Data collection | Pen and paper were used to collect consent, demographic data, and self-report measures of general spatial abilities. Computer was used to collect data for the spatial navigation task. |
| Timing | Start: April 2023. Stop: November 2025 |
| Data exclusions | In the primary analysis, two participants were excluded. One participant did not complete any usable data due to motion sickness. One participant did not follow task instructions correctly and provided invalid data. In the supplemental analysis, four participants were excluded due to not following task instructions and providing invalid data. |
| Non-participation | One participant did not complete any usable data due to motion sickness. (see above) |
| Randomization | Participants were not allocated into experimental groups. |

# Reporting for specific materials, systems and methods

We require information from authors about some types of materials, experimental systems and methods used in many studies. Here, indicate whether each material, system or method listed is relevant to your study. If you are not sure if a list item applies to your research, read the appropriate section before selecting a response.

## Materials & experimental systems

| n/a | Involved in the study |
|---|---|
| ☒ | ☐ Antibodies |
| ☒ | ☐ Eukaryotic cell lines |
| ☒ | ☐ Palaeontology and archaeology |
| ☒ | ☐ Animals and other organisms |
| ☒ | ☐ Clinical data |
| ☒ | ☐ Dual use research of concern |
| ☒ | ☐ Plants |

## Methods

| n/a | Involved in the study |
|---|---|
| ☒ | ☐ ChIP-seq |
| ☒ | ☐ Flow cytometry |
| ☒ | ☐ MRI-based neuroimaging |

## Plants

| | |
|---|---|
| Seed stocks | N/A |
| Novel plant genotypes | N/A |
| Authentication | N/A |
