# Supplementary material for: Environment geometry alters sequential route learning and its integration into cognitive maps
Source: Nat Commun. 2026 Jul 7;17:8400. doi: 10.1038/s41467-026-75129-y (PMC13473551; doi:10.1038/s41467-026-75129-y)
Supplement: Supplementary file 3 [file 41467_2026_75129_MOESM3_ESM.pdf]

nature portfolio

# Peer Review File

# Environment Geometry Alters Sequential Route Learning and its Integration into Cognitive Maps

Corresponding Author: Ms Jaida Long

**This file contains all reviewer reports in order by version, followed by all author rebuttals in order by version.**

Version 0:

Reviewer comments:

Reviewer #1

(Remarks to the Author)
The paper reports an experiment which examined spatial memory (SM) for position of 6 objects presented sequentially in either a square or trapezoid arena. The environment was presented on a computer and objects positions where learned in first person view trials and SM was tested in both a first person view and an overhead view. The study was designed to look at three things that could affect SM; shape of arena, order of object presentation and individual differences in sense of direction (SOD). Results showed that 1) participants performance overall was the same in the square as the trapezoid suggesting the irregular environmental geometry on average does not disrupt SM, 2) that SM for objects presented at the end of the trial was worse than at the start supporting previous evidence for increasing errors during path integration and 3) that overall High SOD was associated with better SM but High SOD was associated with worse performance in the narrow end of the trapezoid whether it was presented first or last. Low SOD did not show this pattern. Authors suggested this pattern of results meant that the High SOD participants were more sensitive to the boundary cues and this overshadowed the path integration effect.
The study is well designed, novel and add to the current literature. The conclusions drawn are justified from the results. My only comments are minor regarding clarity of the introduction regarding previous research and the methods used in the study. I have also some questions regarding the results which the authors may consider addressing in a revision.
On page 1 paragraph 1 Line 3 the authors state
In rodents, misalignment between boundaries disrupts neural activity of a theorized biological basis of internal maps, grid cells.1,10,11 Identical boundary distortions also impede human spatial memory (SM) for object locations in a manner commensurate with and predicted by rodent grid distortions.
Given the subsequent frequent referral to grid cell theory predictions, could the authors give a little more detail regarding this association between grid cell disruption by irregular geometry and human SM?
On page 4 paragraph 2 The Santa Barbara Sense of Direction is abbreviated to SBSOD but for the rest of the paper it is abbreviated to SOD. Could this be abbreviated consistently throughout?
On page 4 paragraph 3 the text describes the virtual navigation task. Could this refer to a figure to aid clarity which illustrates the 6 objects from an overhead perspective in both the square and trapezoid arena?
Page 5 paragraph 1 line 2 the authors describe the objects as “environment-unique”. Could the authors clarify what this term means by giving a little more detail and maybe referring to figure 7b where the objects are shown.
Page 6: in the Simple effects of Serial Order analysis they describe the 3 alternative hypotheses and that there is a significant serial order effect but it is only till a later section do they say the Spatial Memory Error (SME) increased along the route suggesting hypothesis 2 was correct.
Page 7 describes the analysis of SME in the Narrow versus Wide end of the trapezoid, then Page 8 describes the SME in Even versus Odd Serial Order. As the odds are in the wide end and Evens in the narrow end I wasn’t sure what having both analyses added. Could the authors just report one?
Discussion section Page 13 Paragraph 1 : I was a little confused why having distal cues in the trapezoid environment would impact SM. I think it would help clarity if the sentence on line 7 read “having multiple redundant cues in this may have also increased cognitive load”.
Last sentence of Paragraph 2 confused me.
“It is plausible that if our measures emphasized self-orientation relative to the environment, as other literatures have done, we’d see facilitation of performance in the trapezoidal space - but our data importantly emphasize that for object location memory, the predominant influence of the environment distortion may be an analogous location memory distortion. “
I presume the authors suggest that asking participants where they were within the arena rather than where objects were the

trapezoid would facilitate performance? If this interpretation is correct could authors explain a bit more why they think this would be the case, given they still have to work out where they are in the arena when indicating the object location in the first person test. If the interpretation is not correct can the authors consider clarifying the sentence.
Paragraph 3, line 3 the authors mention configural knowledge is encoded. Can the authors give example of what configural knowledge?
Page 14 paragraph 2, line 7, I was confused by the sentence "serial order effects in memory were ultimately more consistent with predictions grounded in the noise of neural path integrator functions than competing patterns"
Could this idea be explained in a little more detail?

Page 15 Paragraph 1 Line 1
"greater self-localization information to the navigator in the trapezoid may draw participants to rely more on the boundaries than the distal cues for trying to learn object locations (to their detriment, resulting in larger and more geometrically-aligned memory errors). "
Why would learning the asymmetric geometry of the trapezoid rather than the distal cues lead to poor SM? Earlier it was suggested that the redundant distal cues would increase cognitive load, so wouldn't focussing on boundary cues reduce cognitive load?
Could the difference seen between the first person and overhead test results seen in the High SOD participants be due to seeing all of the boundary cues in the former and only one boundary in the latter. Could the authors include an explanation of why only one boundary was shown in the overhead test?
Typo Paragraph 2 line 7: "We posit that that other.." delete repetition

(Remarks on code availability)

Reviewer #2

(Remarks to the Author)
This paper is a very clear report of a complex study, but it substantially enhances our understanding of spatial memory. I have some suggestions for clarification.
1. Reduce the use of acronyms. In particular, I found the use of SM for spatial memory unnecessary.
2. Structure the discussion with headings, clearly indicating that it begins with a summary, and follows with in-depth discussion of each of several topics. Crucially, add a final paragraph discussing the relevance of the findings for spatial memory "in the wild", where enclosed environments were rare in our environment of adaptation, and suggest a few possibilities for future research.

(Remarks on code availability)

Reviewer #3

(Remarks to the Author)
Understanding the ways in which various mechanisms combine to support spatial memory is a fundamental aim of neurocognitive science. Often times, researchers do their best to experimentally isolate particular mechanisms, leaving unanswered how these combine in less rigidly structured, more naturalistic setttings. Here, Long et al. try to take on these questions, specifically studying the interaction between serial order effects and environmental geometry. To do so, they ask participants to remember and recall object locations along stereotyped paths in large square and trapezoidal environments, utilizing desktop VR. Participants reported both first and third person estimates of locations. From these data, they interpret patterns of absolute error in the context of theoretically-motivated predictions (reasoned, but not modeled): path integration, schema formation, grid cell distortions, misorientation, etc. While I am very sympathetic to the importance of the questions being asked and the theories being contrasted, I have some relatively strong concerns about the strength of the theoretical predictions and the conclusions being drawn from the data as they are.

Major concerns:

(1) The singular measure that the authors rely upon throughout the work is the Euclidean distance between the true and recalled object locations. While this makes sense as a starting point, I believe that this can mask underlying structure to replacement errors which can be informative, and perhaps exaggerates order effects. I would be interested to see whether replacement errors relative to the previously replaced object also show compounding error, and/or interact with geometry in a similar way. Likewise, computing distance after a Procrustes transform might better isolate the distortions the authors expect to observe, as this initial or overall referencing errors could account for overall absolute error differences that cloud underlying structure in replacement errors. Doing so might even tighten results if the authors conclusions are accurate, and might perhaps reveal additional underlying patterns that contribute to the interpretation of the results.

(2) Grid cell distortions are the primary motivation for impoverished spatial memory in the narrow half of the trapezoidal geometry relative to the less-broader half. To my eye it looks like errors in the broader half are actually lower than the errors in the square environment for matched serial positions. Is this true? If so, that seems hard to reconcile with the story. Additionally, with the large scale of the environments I wonder whether the claim of 'grid cells are not distorted in square

environments' actually holds, as large square arenas are reported to include distortions in rodents (Stensola et al., 2015) and the environments being contrasted here are quite large.

(3) While the contrasting of competing theories is in one sense an advantage when it comes to interpretation of the results, the open-ended-ness of possibilities raises (for me) concerns about the generalizability of the results. Because any possible outcome can be interpreted as consistent with some combination of theories, it makes me skeptical of the theoretical conclusions (which are fundamental to the novelty of the work) in the absence of a strong replication. Were you to then show that a similar pattern of results hold across environments of different scales, non-alternating patterns of object replacements, and in a new cohort, I would be significantly more compelled that by the results.

Minor comments:

I think the work would benefit from a graphical presentation of the task and depiction of the views and environments in the main text to better situate the reader to interpret the outcomes, rather than bouncing the reader to the methods at the beginning of the results.

For the correlations with the SBSOD, is there a motivation for the observation of correlations only in the third-person reports of object locations, and for the absence of results in the trapezoid. Were these results corrected for multiple comparisons (two measures x 4 correlations)? Was the contrast between geometries and splits significant?

(Remarks on code availability)

Version 1:

Reviewer comments:

Reviewer #1

(Remarks to the Author)
Thank you for the thoughtful revisions to the manuscript which I feel have addressed my questions. I feel the paper is much clearer following the revisions.

(Remarks on code availability)

Reviewer #2

(Remarks to the Author)
Thanks for a responsive revision. In the new material added under "Conclusions", I do not understand what is meant by the phrase " long-term view of human cognitive development". I think it's the idea of viewing human cognitive development in ecological and evolutionary context. Is that correct?

(Remarks on code availability)

Reviewer #3

(Remarks to the Author)
I appreciate the effort that the authors went to through addressing my concerns. I believe the changes that they made (especially the inclusion of the Procrustes analysis) have addressed my major concerns and that the revision is suitable for publication. Nice work!

Minor comment: It looks like the axes on Page 40 of your supplement are cut off -- I don't need to see the paper again, but these might be fixed prior to publication.

(Remarks on code availability)
I have not attempted to run the code.

**Open Access** This Peer Review File is licensed under a Creative Commons Attribution 4.0 International License, which permits use, sharing, adaptation, distribution and reproduction in any medium or format, as long as you give appropriate credit to the original author(s) and the source, provide a link to the Creative Commons license, and indicate if changes were made.
In cases where reviewers are anonymous, credit should be given to 'Anonymous Referee' and the source.
The images or other third party material in this Peer Review File are included in the article's Creative Commons license, unless indicated otherwise in a credit line to the material. If material is not included in the article's Creative Commons license and your intended use is not permitted by statutory regulation or exceeds the permitted use, you will need to obtain permission directly from the copyright holder.
To view a copy of this license, visit https://creativecommons.org/licenses/by/4.0/

**REVIEWER COMMENTS**

**Reviewer #1** (Remarks to the Author):

The paper reports an experiment which examined spatial memory (SM) for position of 6 objects presented sequentially in either a square or trapezoid arena. The environment was presented on a computer, and objects' positions were learned in first person view trials and SM was tested in both a first person view and an overhead view. The study was designed to look at three things that could affect SM; shape of arena, order of object presentation and individual differences in sense of direction (SOD). Results showed that 1) participants performance overall was the same in the square as the trapezoid suggesting the irregular environmental geometry on average does not disrupt SM, 2) that SM for objects presented at the end of the trial was worse than at the start supporting previous evidence for increasing errors during path integration and 3) that overall High SOD was associated with better SM but High SOD was associated with worse performance in the narrow end of the trapezoid whether it was presented first or last. Low SOD did not show this pattern. Authors suggested this pattern of results meant that the High SOD participants were more sensitive to the boundary cues and this overshadowed the path integration effect.
The study is well designed, novel, and adds to the current literature. The conclusions drawn are justified from the results. My only comments are minor regarding clarity of the introduction regarding previous research and the methods used in the study. I have also some questions regarding the results which the authors may consider addressing in a revision.

**Minor comments**

On page 1 paragraph 1 Line 3 the authors state, "In rodents, misalignment between boundaries disrupts neural activity of a theorized biological basis of internal maps, grid cells.[1,10,11] Identical boundary distortions also impede human spatial memory (SM) for object locations in a manner commensurate with and predicted by rodent grid distortions." Given the subsequent frequent referral to grid cell theory predictions, could the authors give a little more detail regarding this association between grid cell disruption by irregular geometry and human SM?

Thank you for raising this concern. We agree that it is critical to provide detail regarding the association between grid cell disruption, irregular geometry, and human spatial memory. To respond, we have provided details in paragraph 2 in the introduction, where we stated:

"For example, Bellmund et al. 2020 report that spatial memory for single object locations is worse in trapezoids compared to squares in humans.[12] Grid cell recordings from rodents provide a potential mechanism, demonstrating distorted firing fields in trapezoids, particularly the narrow end.[10]"

Furthermore, there are additional details in paragraph 3 of the discussion:

"Many studies have linked the integrity of grid cells to environment boundaries, especially those with varying geometry.[1,11,36–39] In highly polarized enclosures, such as trapezoids, distortions to grid cell firing fields are more severe.[10,12] Computational models demonstrate significantly impaired positional decoding in such enclosures,[12] and trapezoids distort grid patterns in rodents and accuracy of spatial memories in humans, respectively.[10,12] Both effects have been shown to be more pronounced in the narrow end, the most polarized region of the trapezoid."

And finally, we have added an additional detail (introduction, paragraph 2, after sentence 3) based on a recently published paper:

"Furthermore, a recent study not only replicated the distortion of rodent grid cell firing fields in trapezoid environments, but also observed significant correlations between these distortions and spatial memory deficits in those same rodents.[18]"

We hope that these details are sufficient. If they are not, please let us know and we are happy to include additional details.

On page 4 paragraph 2 The Santa Barbara Sense of Direction is abbreviated to SBSOD but for the rest of the paper it is abbreviated to SOD. Could this be abbreviated consistently throughout?

We thank the reviewers for raising this concern, and we understand the potential for confusion between SBSOD and SOD. We would like to clarify that although the two acronyms are similar, they refer to slightly different things in our manuscript: sense of direction (SOD) is intended to mean the general (self-report) ability to know one's location and perform wayfinding. The Santa Barbara Sense of Direction scale (SBSOD), is the method by which we used to assess sense of direction (SOD). To respond to this concern, we have clarified this in page 4 paragraph 2 of our updated manuscript to address this confusion:

"An individual's aptitude for performing wayfinding and knowing one's location, an ability termed sense of direction (SOD)[33], is variable … The Santa Barbara Sense of Direction (SBSOD) scale[34] is a metacognitive measure of SOD that predicts performance in naturalistic wayfinding, virtual, and vicarious navigation.[16,17,35] Critically, we have employed the SBSOD to consistently show that …"

On page 4 paragraph 3 the text describes the virtual navigation task. Could this refer to a figure to aid clarity which illustrates the 6 objects from an overhead perspective in both the square and trapezoid arena?

We appreciate this suggestion and have added the requested figure into page 5 of the updated manuscript:

**1**

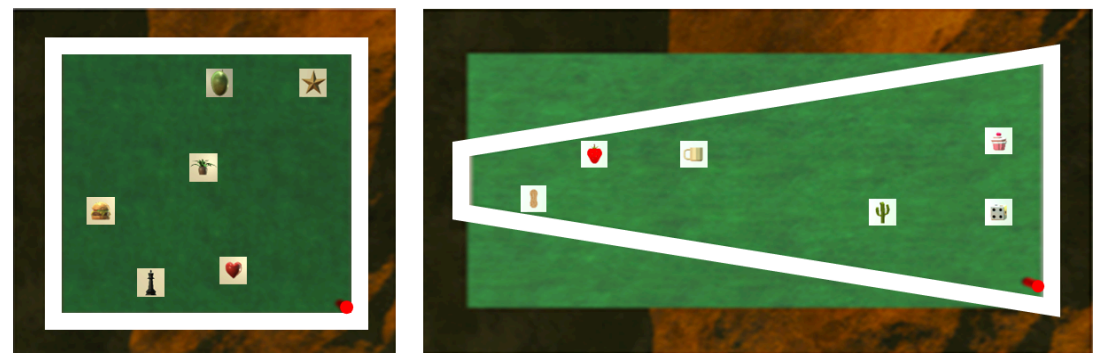

Figure 1. **Overhead View of Task.** Examples of a square and trapezoid environment are displayed, along with icons indicating the identity and location of the objects that were present in each respective environment (for all object identities and routes see Supplement). During the task, participants navigated first-person between the six object locations within environments (two squares and two trapezoids) along a fixed route to learn the objects' locations and sequence. Then, participants' spatial memory for object locations was assessed from first-person and overhead perspectives.

Page 5 paragraph 1 line 2 the authors describe the objects as "environment-unique". Could the authors clarify what this term means by giving a little more detail and maybe referring to figure 7b where the objects are shown.

We appreciate this suggestion, and we have added additional details regarding this into the manuscript, page 6:

"Each environment's set of six objects was distinct from the other three environments (see Supplement)."

Furthermore, we have added information regarding the object identities into the supplementary materials to provide further details:

**"Environment Objects**

Square variant 1 (green floor): heart, star, burger, chesspiece, plant, mango

Square variant 2 (brown floor): plantpot, pear, tree, cherries, pencil, rubixcube

Trapezoid variant 1 (green floor): cupcake, peanut, dice, cup cactus, strawberry

Trapezoid variant 2 (brown floor): orange, flower, arcade, lemon, snowman, boat

Objects are listed in the serial order of the route. Note that the order of the environment variants was counterbalanced and pseudorandomized across participants."

Page 6: in the Simple effects of Serial Order analysis they describe the 3 alternative hypotheses and that there is a significant serial order effect but it is only till a later section do they say the Spatial Memory Error (SME) increased along the route suggesting hypothesis 2 was correct.

We appreciate this suggestion, and we have added a brief interpretation of this result in the paragraph (now on page 7) these hypotheses are stated:

"simple effects of serial order are best explained by theories of path integration error accumulation."

Page 7 describes the analysis of SME in the Narrow versus Wide end of the trapezoid, then Page 8 describes the SME in Even versus Odd Serial Order. As the odds are in the wide end and Evens in the narrow end I wasn't sure what having both analyses added. Could the authors just report one?

Thank you for your feedback. Upon reviewing this section, we agree that the clarity of the novelty/importance of this analysis could be improved, and the additional terms/labels of Even and Odd were unnecessary. Therefore, we reworded the section (now pages 9-10, text not reproduced here for conciseness) to add clarity, and to also emphasize the important additional contribution of the analyses: these analyses demonstrate shape effects comparing trapezoid subregions to the square *separately* (narrow vs. square, wide vs. square) - and critically, these analyses controlled for object serial orders. This gives additional insight into the behavioral data because the other analyses compared the narrow end to the wide end, or the square to the whole trapezoid (narrow and wide).

Discussion section Page 13 Paragraph 1 : I was a little confused why having distal cues in the trapezoid environment would impact SM. I think it would help clarity if the sentence on line 7 read "having multiple redundant cues in this may have also increased cognitive load".

Thank you for raising these concerns. We have updated the manuscript to improve the clarity of these sentences (now page 15, paragraph 1):

"For example, the reliable orientation information provided by distal cues (which is constant regardless of location within the local environment) may have been in competition/conflict with the distorted depth and orientation information provided by the proximal environment boundaries in the trapezoids (which depends on the navigator's current perspective)."

Last sentence of Paragraph 2 confused me.
"It is plausible that if our measures emphasized self-orientation relative to the environment, as other literatures have done, we'd see facilitation of performance in the trapezoidal space - but our data importantly emphasize that for object location memory, the predominant influence of the environment distortion may be an analogous location memory distortion. "
I presume the authors suggest that asking participants where they were within the arena rather than where objects were the trapezoid would facilitate performance? If this interpretation is correct could authors explain a bit more why they think this would be the case, given they still

have to work out where they are in the arena when indicating the object location in the first person test. If the interpretation is not correct can the authors consider clarifying the sentence.

We apologize for the confusion. Our intended meaning was asking participants about the direction of an object location relative to their location rather than identifying the precise spatial location of an object. To respond, we have clarified the sentence (now end of page 15):

"It is plausible that if we assessed self-orientation relative to the objects (as other literatures have done[21]) rather than the object locations themselves, we would observe better performance in the trapezoid - but our data importantly emphasize that distorting environment boundaries (trapezoid) correspondingly distorts object location memories, rather than improving them."

Paragraph 3, line 3 the authors mention configural knowledge is encoded. Can the authors give an example of what configural knowledge is?

Thank you for your suggestion. We have expanded upon the term for clarity (end of page 15):

"where configural knowledge, the knowledge of spatial relationships between objects and their features, ..."

Page 14 paragraph 2, line 7, I was confused by the sentence "serial order effects in memory were ultimately more consistent with predictions grounded in the noise of neural path integrator functions than competing patterns"
Could this idea be explained in a little more detail?

Thank you for your suggestion. We have rewritten the sentence for clarity (end of page 16):

"the serial order effects that we observed were more consistent with predictions grounded in path integration research, where grid cells are also believed to play a critical role."

Page 15 Paragraph 1 Line 1
"greater self-localization information to the navigator in the trapezoid may draw participants to rely more on the boundaries than the distal cues for trying to learn object locations (to their detriment, resulting in larger and more geometrically-aligned memory errors). "
Why would learning the asymmetric geometry of the trapezoid rather than the distal cues lead to poor SM? Earlier it was suggested that the redundant distal cues would increase cognitive load, so wouldn't focusing on boundary cues reduce cognitive load?

Thank you for raising this concern. We agree that the wording of the sentence was unclear and have now updated the sentence (now page 17):

"We speculate that because they are not symmetrical, the trapezoid boundaries' appearance of offering greater information for self-localization (true to an extent because of the

polarity) may draw participants to rely more on the boundaries in the trapezoid than they would in the square (where the symmetry dramatically reduces how informative the walls are about current heading or location). However, from both the grid-cell-based mechanistic framework and a cue-competition perspective, such reliance on both cue sources in the trapezoid instead of predominantly the distal ones would be to their detriment, resulting in larger and more geometrically-aligned memory errors. Here, cue competition especially arises from the grid-cell based framework: the precision/signaling of heading and location does not fully agree between cue sources if one source [geometry] distorts spatial perception)."

Could the difference seen between the first person and overhead test results seen in the High SOD participants be due to seeing all of the boundary cues in the former and only one boundary in the latter. Could the authors include an explanation of why only one boundary was shown in the overhead test?

Thank you for raising this concern. To respond to this concern, we have clarified in the main text, (page 22, Overhead test):

"Only one boundary was present in the overhead test because we did not want to reveal the shape of the environments to participants while they completed the study, and we did not want to potentially constrain inter-object placement in a way that could mask how accurate/inaccurate the relative angles and distances between the objects were mapped in memory."

Given our prior evidence that high SOD are more sensitive to additional spatial cues, we believe that hiding the boundaries in the overhead test, and the larger overall range for possible errors, increased our sensitivity to disruptive boundary effects in long-term memory for trapezoids that come with higher SOD, which we posit might be overcome to an extent in the first-person test thanks to online error-correction that can be done by orienting and reorienting over the course of active navigation.

Typo Paragraph 2 line 7: "We posit that that other.." delete repetition
We appreciate you notifying us of this typo; it has been addressed.

**Reviewer #2** (Remarks to the Author):

This paper is a very clear report of a complex study, but it substantially enhances our understanding of spatial memory. I have some suggestions for clarification.

**Comments**

1. Reduce the use of acronyms. In particular, I found the use of SM for spatial memory unnecessary.

This is a fantastic suggestion–we would be happy to do it, but unfortunately it is against the formatting guidelines for the article, so we did not add new subheadings.

2. Structure the discussion with headings, clearly indicating that it begins with a summary, and follows with in-depth discussion of each of several topics. Crucially, add a final paragraph discussing the relevance of the findings for spatial memory "in the wild", where enclosed environments were rare in our environment of adaptation, and suggest a few possibilities for future research.

Thank you for your suggestions regarding our discussion. To respond to this concern, we have added subheadings and strengthened our final paragraphs (now on page 18):

"Collectively, our results show that the precision of spatial memories is influenced by path integration along a route, individual capacities for SOD, and the structural regularity of the environment in which the route memory is situated. In complex real-world environments, the regularity of environment geometry varies dramatically. This variation is highly relevant for human navigation, given that urban and human-made environments are increasingly ubiquitous and complex. From an evolutionary perspective, distinctive geometric patterns and complexities of environment barriers would have been relatively rare "in the wild" for much of our species' development. It is plausible that this long-term view of human cognitive development contributes to participants' inaccuracies perceiving and remembering depths, distances, and angles in the face of our manipulation.

Our study lays the foundation for future studies to further expand upon. Future research on the influence of environment scales, as well as the influence of varying geometries and geometric distinctiveness (e.g. from major to minor uncertainty levels: circular, rectangular, trapezoidal with varying wall lengths, and kite-shaped environments), will be essential. Additionally, from an ecological perspective, it will be of interest to explore how well-defined and contiguous boundaries influence our memory system (e.g., row of trees vs. brick wall; straight line of road vs. irregular edges of riverbank). Finally, there is a clear opportunity to bridge our work more closely with studies of how upbringings in more rural vs. urban settings influence spatial memory performance in different environments.[62] Taken together, by establishing how key factors of path integration, individual capacities, and environment geometries interact to modulate and predict spatial memory in naturalistic navigation, our findings provide a critical foundation for future navigation research."

**Reviewer #3** (Remarks to the Author):

Understanding the ways in which various mechanisms combine to support spatial memory is a fundamental aim of neurocognitive science. Oftentimes, researchers do their best to

experimentally isolate particular mechanisms, leaving unanswered how these combine in less rigidly structured, more naturalistic settings. Here, Long et al. try to take on these questions, specifically studying the interaction between serial order effects and environmental geometry. To do so, they ask participants to remember and recall object locations along stereotyped paths in large square and trapezoidal environments, utilizing desktop VR. Participants reported both first and third person estimates of locations. From these data, they interpret patterns of absolute error in the context of theoretically-motivated predictions (reasoned, but not modeled): path integration, schema formation, grid cell distortions, misorientation, etc. While I am very sympathetic to the importance of the questions being asked and the theories being contrasted, I have some relatively strong concerns about the strength of the theoretical predictions and the conclusions being drawn from the data as they are.

**Major concerns**

(1) The singular measure that the authors rely upon throughout the work is the Euclidean distance between the true and recalled object locations. While this makes sense as a starting point, I believe that this can mask underlying structure to replacement errors which can be informative, and perhaps exaggerates order effects. I would be interested to see whether replacement errors relative to the previously replaced object also show compounding error, and/or interact with geometry in a similar way. Likewise, computing distance after a Procrustes transform might better isolate the distortions the authors expect to observe, as this initial or overall referencing errors could account for overall absolute error differences that cloud underlying structure in replacement errors. Doing so might even tighten results if the authors conclusions are accurate, and might perhaps reveal additional underlying patterns that contribute to the interpretation of the results.

Thank you, we truly appreciate this suggestion. We agree that computing Euclidean distance after a Procrustes transformation adds critical insight to our results. To address this comment fully, we replicated all of our analyses with Procrustes-transformed data (see page 13). This revealed that, overall, the pattern of results we initially reported was the same, with a few minor differences (see below). Overall, we believe this multidimensional scaling technique firmly strengthens our results.

In main text:

**"Procrustes Analysis**

Our primary analyses measured spatial memory performance by the Euclidean distance between true object locations and participants' responses. To precisely account for initial or overall reference errors (e.g. scaling, translation, rotation, reflection), possibly clouding the underlying structure of spatial memory error, we performed a secondary set of analyses to determine the patterns of spatial memory error accounting for such overall referencing errors. To

do this, the raw data for these analyses were Procrustes transformed in MATLAB, which controlled for the influence of scaling, translation, rotation, and reflection on the overall response profile of our measured Euclidean errors. The analyses of Procrustes-transformed data revealed, overall, effects that were consistent with the analyses of the raw data. Qualitatively, which effects were statistically significant or non-significant remained the same across our error measures of interest with the Procrustes transforms (see Supplement)."

Added to Supplement:

"Qualitatively, which effects were statistically significant or non-significant remained the same across our error measures of interest with the Procrustes transforms, including: effects of trapezoid subregion on spatial memory error, object serial order, object serial order * Shape, SOD, SOD*Shape, and matched-serial order objects in the square vs. the narrow end of the trapezoid. Exceptions to note were: 1. main effect of Shape across all participants (raw data: non-significant; vs. Procrustes data: marginally significant), suggesting that accounting for the referencing errors marginally increased sensitivity to an overall difference between squares and trapezoids, and 2. SOD*Shape interactions for individuals with high SOD in the overhead test (raw data: significant; vs. Procrustes data: marginally significant). Finally, when comparing matched-serial order objects in the square vs. the wide end of the trapezoid, the difference was non-significant for the Procrustes data, vs. in the raw data, spatial memory error in the wide end of the trapezoid error was significantly lower than the square. Given that most of our effects remained qualitatively the same, these analyses collectively suggest that our observed results are, largely, due to more focal errors in object-location memory and not due to overall referencing errors (see below for detailed statistics of the Procrustes control analyses).

With the Procrustes transform as a control, the interaction between object serial order and Shape remained significant (first-person: $p = .006$, $F = 3.265$, $df = 5, 5140.14$; overhead: $p < .001$, $F = 5.899$, $df = 5, 1584.56$), as well as the overall effect of object serial order (first-person: $p < .001$, $F = 15.065$, $df = 5, 5140.14$; overhead: $p < .001$, $F = 5.550$, $df = 5, 1584.54$). Spatial memory error was still significantly higher in the narrow end of the trapezoid than the wide end (first-person: $p < .001$, $F = 28.149$, $df = 1, 2578.17$; overhead: $p < .001$, $F = 29.388$, $df = 1, 794.49$). Comparing objects with matched object serial orders, spatial memory error was still significantly higher for objects in the narrow end of the trapezoid than the square (first-person: $p < .001$, $F = 18.023$, $df = 1, 2470.22$; overhead: $p < .001$, $F = 27.947$, $df = 1, 722.12$).

The overall influence of Shape (collapsing across the route), while not significant for the raw data, was marginally significant for the Procrustes-transformed data (first-person: $p = .053$, $F = 3.752$, $df = 1, 796.12$; overhead: $p = .051$, $F = 3.855$, $df = 1, 213.29$). Moreover, when comparing matched-serial-order objects in the square to those in the wide end of the trapezoid, the Procrustes-transformed data yielded a non-significant effect (first-person: $p = .637$, $F = .223$, $df = 1, 2469.86$; overhead: $p = .882$, $F = .022$, $df = 1,761.79$), while the raw data indicated significantly lower spatial memory error in the wide end of the trapezoid. Analysis of this same

effect on Procrustes-transformed data that does not permit scaling revealed a non-significant effect as well (first-person: $p = .113$, $F = 2.516$, $df = 1, 2470.54$; overhead: $p = .703$, $F = .145$, $df = 1, 762.22$), indicating that the difference was not due to scaling reference errors.

Finally, the main effect of SOD remained significant (first-person: $p = .019$, $F = 5.779$, $df = 1, 73.38$; overhead: $p = .033$, $F = 4.721$, $df = 1, 73.94$), as well as the SOD*Shape interaction in the overhead test ($p = .033$, $F = 4.621$, $df = 1, 207.87$). The SOD*Shape interaction in the first-person test remained non-significant ($p = .335$, $F = .932$, $df = 1, 773.23$). With the raw data, the SOD*Shape interaction in the overhead test was significant for high SOD individuals. This effect was marginal for the Procrustes-transformed data ($p = .055$, $F = 3.766$, $df = 1, 103.08$). For low SOD individuals, the SOD*Shape interaction remained null (see below)."

(2) Grid cell distortions are the primary motivation for impoverished spatial memory in the narrow half of the trapezoidal geometry relative to the less-broader half. To my eye it looks like errors in the broader half are actually lower than the errors in the square environment for matched serial positions. Is this true? If so, that seems hard to reconcile with the story. (page 16)

The reviewer is correct that the errors in the broader half of the trapezoid are lower than the errors in the square environment for matched serial positions. In our discussion section we theorized that this may be due to the competing underlying mechanisms we tested with our design - namely, an interplay between geometric distinctiveness and environment geometry influences on grid cell distortion effects. To respond to this comment we have revised and clarified this Discussion point further (page 15):

"Our study sheds light on these competing perspectives. In our design, we demonstrate a clear effect of significantly worse spatial memory in the narrow end of trapezoid compared to the square. This is consistent with the predictions from existing grid cell studies that suggest that trapezoidal boundaries distort grid cell firing fields, and, subsequently, spatial memory. However, these studies also predict that spatial memory in the wide end of the trapezoid would be worse than in the square. Not only was this effect absent in our results, but we also demonstrate evidence for the opposite: that spatial memory was worse in the square than the wide end of the trapezoid. We posit that other factors, such as geometric distinctiveness facilitating orientation memory in the trapezoid relative to the square, may interact with grid cell-based mechanisms for the ultimate outcomes of geometry on SM: namely, in the wide end of the trapezoid, underlying grid cell distortions may be relatively minimal (as others have shown); at the same time, the distinctive polarity of the trapezoid boundaries facilitates orientation information - but such a benefit can be offset by metric distortions in memory from grid cell disruptions when moving into the narrower end of a trapezoid. With this interaction in place, we theorize that when grid cell firing patterns are minimally distorted, as we know is the case in the wide end of a trapezoid, impairments may be minimal or facilitation effects may even be observed. It is plausible that if we assessed self-orientation relative to the objects (as other literatures have done[21]) rather than

the object locations themselves, we would observe better performance in the trapezoid - but our data importantly emphasize that distorting environment boundaries (trapezoid) correspondingly distorts object location memories, rather than improving them. Supporting this, our supplemental Procrustes analysis of this result revealed that the benefit in spatial memory in the wide end of the trapezoid was accounted for by rotational and translational referencing but not scaling (other factors the analysis tested for)."

Additionally, with the large scale of the environments I wonder whether the claim of 'grid cells are not distorted in square environments' actually holds, as large square arenas are reported to include distortions in rodents (Stensola et al., 2015) and the environments being contrasted here are quite large.

Thank you for raising this concern. Our wording of this sentence was not accurate and we have updated it (page 4, last paragraph):

> "Importantly, square environments do not distort grid cell firing fields as much as trapezoids".

We would also like to clarify that the scale of our environment is not particularly large - the low walls (allowing the participant to perceive the distal cues) may give an illusion of the environment being larger than it is. Rather, our environments were built to carefully match the scale and parameters of Bellmund et al., 2020 and approximate the size relative to the navigator (based on the user's virtual "body size" from their "head height") that was experienced in some of the landmark rodent work inspiring our study (Krupic et al., 2015). Please see the "Environment Design" section on page 22.

(3) While the contrasting of competing theories is in one sense an advantage when it comes to interpretation of the results, the open-ended-ness of possibilities raises (for me) concerns about the generalizability of the results. Because any possible outcome can be interpreted as consistent with some combination of theories, it makes me skeptical of the theoretical conclusions (which are fundamental to the novelty of the work) in the absence of a strong replication. Were you to then show that a similar pattern of results hold across environments of different scales, non-alternating patterns of object replacements, and in a new cohort, I would be significantly more compelled by the results.

We really appreciate this point - we certainly feel the strength of our approach to lend support to A vs. B vs. C outweighs the cons, especially to the extent that it connects with broader memory frameworks outside the navigation field alone, but the concern certainly resonates with us as well, and it is indeed an aspect that made introducing and discussing the project more

challenging to write. In particular, we agree that it is important to know if the results are a) reproducible and b) generalize to different scenarios.

First, we want to clarify that our scale was moderate in size and intended to extend prior work establishing evidence of grid metric distortion (see response to Comment 2). With this in mind, and given the importance of cue availability and distortion to our competing theoretical views, we decided to respond to this concern with a reproducibility and generalization dataset not with a scale manipulation, but one where we ran the study again in a new cohort and manipulated the cue makeup. While we appreciate that this leaves the question of scale sensitivity in the results open for future work (see Conclusion), we hope the reviewer finds this responsive and agrees this new dataset addresses the spirit of their concern while tying in more closely with the frameworks we set out to test in the article.

To briefly summarize the results of this new dataset: they suggest that our results and interpretations hold true across conditions where local geometry is paired with distal cues (the original dataset) and where distal cues are removed and both the geometric and orienting signals are provided by the local intramaze cues (removing competition between sources and equating whether all of the necessary cues for orientation are proximal [intramaze] across environment shapes as factors). This was especially true for the first-person test, but there were some notable differences for the overhead test.

We reported the changes to the design in this second dataset and the results' differences as concisely as possible (page 13), to not distract from the key findings or to overwhelm/overcomplicate the results section of this paper, reproduced here in the response letter:

" Several types of environment cues were available to participants during the task, including distal cues (mountains, clouds, and sun) and proximal or local cues (environment walls with the same color and texture). Since environment cues and their proximity can significantly influence spatial memory, we ran a third set of analyses to determine the generalizability of our results to environments with different cue availability (i.e., local instead of distal landmark information). We collected a new cohort of healthy young adults (N = 82, 52 female, age = 20.049 ± 2.233) from Georgia Tech and the surrounding Atlanta area called the Non-Global cohort, who performed the task identically to the previous cohort, called the Global cohort. We presented similar square and trapezoid environments again with the same 6-object routes in a counterbalanced structure. The only difference for this new cohort was the environment cues themselves: distal cues were removed, and the environment walls were changed to have distinct colors and textures (local cues) in both environments, thus, replacing distal with proximal landmark information (see Supplement for extended methodology). The same analyses were replicated for this Non-Global condition (see Supplement for details and the specific statistical

results from this reproducibility and generalization test). Importantly, in qualitative terms the majority of the results we report from the Global condition maintained the same pattern of statistical significance in this Non-Global condition."

Added to the Supplement (see Supplement for full methods, figures, tables):

" Importantly, in qualitative terms, the majority of the results we report from the Global condition maintained the same pattern of statistical significance in this Non-Global condition for the first-person tests: Shape, trapezoid subregion, object serial order, object serial order * Shape, SOD, and comparing matched-serial order objects in the square vs. the narrow end of the trapezoid. Only one effect changed in significance: matched-serial order objects in the square vs. the wide end of the trapezoid, which showed significantly better performance in the trapezoid in our Global condition, did not differ between environment geometries in the Non-Global. This means that a facilitative effect of the wide end of the trapezoid compared to the square was present in the Global condition but not in the Non-Global condition (see below for details). This would be consistent with our environmental distinctiveness framework; since the square walls had different textures, they no longer offered impoverished orientation information compared to the wide end of the trapezoid. Taken together, our first-person navigation results generalized to conditions where all distal cues were removed and local environment boundary cues were modified to provide orientation information.

Similarly, for the overhead test, the pattern of statistical significance of the following effects remained the same as in our Global condition: trapezoid subregion, object serial order, object serial order * Shape, SOD, and comparing matched-serial order objects in the square vs. the wide end of the trapezoid. However: 1) The effect of Shape became significant in the Non-Global condition (reflecting better spatial memory overall in the trapezoid). 2) Shape*SOD was no longer significant in the Non-Global condition, and 3) There was no significant difference between the narrow end of the trapezoid and the square when comparing matched-serial order objects. Given the broad agreement between cohorts and environment designs for the first person test, but these different significance patterns in the overhead tests, we suggest the nature of the memory targeted in the overhead probes may be more sensitive to cue availability than active navigation / first person probes. Overall, this preliminary data strongly motivates future studies designed to test the difference between the probe reference frame and the sensitivity of memory distortions to environment cues more deeply.

**Statistics Summary:**

For the following effects, the pattern of statistical significance remained the same in the Non-Global condition, for both first-person and overhead tests.The interaction between object serial order and Shape remained significant in the Non-Global condition (first-person: $p < .001$, $F = 4.640$, $df = 5, 5323.08$; overhead: $p < .001$, $F = 13.232$, $df = 5, 1720.13$), as well as the overall effect of object serial order (first-person: $p < .001$, $F = 30.851$, $df = 5, 5323.08$; overhead: p <

.001, F = 16.740, df = 5, 1720.13). Spatial memory error was still significantly higher in the narrow end of the trapezoid than the wide end (first-person: p < .001, F = 46.648, df = 1, 2653.67; overhead: p < .001, F = 145.173, df = 1, 832.28).

In the first-person test, the significance of the following additional effects remained the same in the Non-Global condition: Shape (p = .618, F = .248, df = 1, 824.52), SOD (p < .001, F = 15.210, df = 1, 75.05), and comparing matched-serial order objects in the square vs. the narrow end of the trapezoid (p = .025, F = 5.059, df = 1, 2559.91). However, when comparing matched-serial order objects in the square vs. the wide end of the trapezoid, the result was nonsignificant in the Non-Global condition (p = .122, F = 2.398, df = 1, 2560.09), while it was significant in the Global condition.

In the overhead test, Shape was significant in the Non-Global condition (p < .001, F = 11.256, df = 1, 224.13), while it was not in the Global condition. Furthermore, SOD*Shape was no longer significant in the Non-Global condition (p = .652, F = .204, df = 1, 217.28), nor was the difference between the narrow end of the trapezoid and the square when comparing objects with matched serial orders (p = .771, F = .084, df = 1, 800.23)."

**Minor comments**

I think the work would benefit from a graphical presentation of the task and depiction of the views and environments in the main text to better situate the reader to interpret the outcomes, rather than bouncing the reader to the methods at the beginning of the results.

Thank you for your suggestion. We have added a figure in the main text to provide more context for the task. (now page 5)

For the correlations with the SBSOD, is there a motivation for the observation of correlations only in the third-person reports of object locations, and for the absence of results in the trapezoid. Were these results corrected for multiple comparisons (two measures x 4 correlations)? Was the contrast between geometries and splits significant?

We apologize for the confusion: we used the same omnibus model to test for the effect of SOD on SME, along with its interaction with other factors (including Shape), in both the first-person and overhead tests (see pages 11-12). The Shape*SOD interaction was only significant in the overhead test, not the first-person test. The analyses regarding the correlations in the overhead tests (Figure 5) were all post-hoc models to understand what factors were driving the interaction in the overhead test.

On page 11, end of paragraph 2 through beginning of paragraph 3:

"The Shape*SOD interaction was not significant in the first-person test (p = .611, F = .258, df = 1, 773.28), but was clear in the overhead test (p = .010, F = 6.766, df = 1, 204.48) (Supp. 3) … To formally test if this interaction was driven by increasing sensitivity to Shape in

high SOD individuals, we median-split high (SOD ≥ 4.6) and low SOD (SOD < 4.6) individuals' overhead test data and re-computed the parameters."

We did not correct for multiple comparisons since these analyses were targeted to understand the effects observed in the larger omnibus model, and we have added an acknowledgment of that to the revised text to be transparent to the reader about this on page 12:

"It should be noted we did not correct these analyses for multiple comparisons because they were targeted post hoc tests specifically to identify which relationships drove the significant result in the omnibus model."

**REVIEWERS' COMMENTS**

Reviewer #1 (Remarks to the Author):

Thank you for the thoughtful revisions to the manuscript which I feel have addressed my questions. I feel the paper is much clearer following the revisions.

Thank you, reviewer 1!

Reviewer #2 (Remarks to the Author):

Thanks for a responsive revision. In the new material added under "Conclusions", I do not understand what is meant by the phrase " long-term view of human cognitive development". I think it's the idea of viewing human cognitive development in ecological and evolutionary context. Is that correct?

Yes! We have clarified this sentence to address your point: "It is plausible that this long-term view of human cognitive development in an ecological/evolutionary context contributes to participants' inaccuracies perceiving and remembering depths, distances, and angles in the face of our manipulation."

Reviewer #3 (Remarks to the Author):

I appreciate the effort that the authors went to through addressing my concerns. I believe the changes that they made (especially the inclusion of the Procrustes analysis) have addressed my major concerns and that the revision is suitable for publication. Nice work!

Minor comment: It looks like the axes on Page 40 of your supplement are cut off -- I don't need to see the paper again, but these might be fixed prior to publication.

Reviewer #3 (Remarks on code availability):

I have not attempted to run the code.

Thank you, reviewer 3! We have corrected the formatting of the indicated figures.
